# The genetic basis of multiple sclerosis: a model for MS susceptibility

**DOI:** 10.1186/1471-2377-10-101

**Published:** 2010-10-28

**Authors:** Douglas S Goodin

**Affiliations:** 1 Department of Neurology, University of California, San Francisco, CA, USA

## Abstract

**Abstact:**

**Background**

MS-pathogenesis is known to involve both multiple environmental events, and several independent genetic risk-factors.

**Methods**

A model of susceptibility is developed and a mathematical analysis undertaken to elucidate the nature of genetic susceptibility to MS and to understand the constraints that are placed on the genetic basis of MS, both by the known epidemiological facts of this disease and by the known frequency of the HLA DRB1*1501 allele in the general populations of northern Europe and North America.

**Results**

For the large majority of cases (possibly all), MS develops, in part, because an individual is genetically susceptible. Nevertheless, 2.2% or less of the general population is genetically susceptible. Moreover, from the model, the number of susceptibility-loci that need to be in a "susceptible allelic state" to produce MS-susceptibility is small (11-18), whereas the total number of such susceptibility-loci is large (50-200), and their "frequency of susceptibility" is low (i.e., ≤ 0.12). The optimal solution to the model equations (which occurs when 80% of the loci are recessive) predicts the epidemiological data quite closely.

**Conclusions**

The model suggests that combinations of only a small number of genetic loci in a "susceptible allelic state" produce MS-susceptibility. Nevertheless, genome-wide associations studies with hundreds of thousands of SNPs, are plagued by both false-positive and false-negative identifications and, consequently, emphasis has been rightly placed on the replicability of findings. Nevertheless, because genome-wide screens don't distinguish between true susceptibility-loci and disease-modifying-loci, and because only true susceptibility-loci are constrained by the model, unraveling the two will not be possible using this approach.

The model also suggests that HLA DRB1 may not be as uniquely important for MS-susceptibility as currently believed. Thus, this allele is only one among a hundred or more loci involved in MS susceptibility. Even though the "frequency of susceptibility" at the HLA DRB1 locus is four-fold that of other loci, the penetrance of those susceptible genotypes that include this allele is no different from those that don't. Also, almost 50% of genetically-susceptible individuals, lack this allele. Moreover, of those who have it, only a small fraction (≤ 5.2%) are even susceptible to getting MS.

## Background

Chronic human diseases such as multiple sclerosis (MS) often have complex etiological bases [1] and, in general, both the genetic makeup of an individual and their environmental experience are critical components of disease pathogenesis. For example, an individual from northern Europe or Canada has a life-time MS-risk [P(MS)] of about 0.1 - 0.2% [2]. The risk in individuals with an affected family member increases roughly in proportion to the amount of shared genetic information between affected relative and the individual [2-9]. Thus, first degree relatives (50% genetic similarity) such as siblings, parents, and children of an MS proband have a risk of approximately a 2-5%, second degree relatives (25% genetic similarity) such as aunts and uncles have a risk of about 1-2% risk, and third degree relatives (with 12.5% genetic similarity) such as first cousins, have a risk less than a 1%. By contrast, monozygotic-twins of an MS proband (~100% genetic similarity) have a risk of about 25% [2-9]. This proband-wise monozygotic-twin concordance rate (CR_MZ_) provides an estimate of the penetrance of MS under similar environmental circumstances. The fact that this penetrance is only 25%, clearly implicates environmental factors in disease pathogenesis and, in fact, there seem to be multiple factors involved, each acting at different periods in a person's life [10]. Nevertheless, as indicated by these recurrence risks in family members of MS probands, genetic factors that render certain individuals susceptible to getting MS also form a critical part of the causal pathway leading to MS.

To date, the best-established genetic association with susceptibility to MS is mapped to the chromosomal region 6p21.3. Within this region, the HLA DRB1 locus (and especially the haplotype that includes the DRB1*1501 allele) has the strongest and most consistent association with MS in both northern European and North American populations [11-15]. Nevertheless, it is striking that, even for individuals who carry the HLA DRB1*1501 allele, only a very small fraction ever develop MS. This observation seems to implicate the critical presence other susceptibility alleles at different locations and, indeed, it seems very likely that genetic susceptibility to MS is determined by the involvement of multiple genetic loci scattered throughout the genome.

The present paper explores, through a mathematical Model and analysis, the nature of this genetic susceptibility to MS. This is not to downplay the importance of environmental factors in disease pathogenesis, which were considered in greater detail previously [10]. Neither is it is an exploration of the genes themselves. Rather, it is an exploration to understand the nature of susceptibility and to delineate the constraints that are placed on the genetic basis of MS both by the known epidemiological facts of this disease and by the known frequency of the HLA DRB1*1501 allele in the general populations of northern Europe and North America and in the MS patients who live in these geographical areas.

## Methods

### The Nature of the Genetic Model

The definitions for the terms used in the model are presented in Table 1. The basic epidemiological data used in model development are presented in Table 2.

**Table 1:** Model Definitions and Abriviations

a_h_	=	allelic frequency of the HLA DRB1*1501 susceptibility allele in the general population (only one copy needed for susceptibility)
a_hm_	=	allelic frequency of the HLA DRB1*1501 susceptibility allele in the MS population (a_hm _= 0.328 in UCSF database)

a_1_, a_2_, a_3_	=	expected allelic frequency of dominant (a_1_), recessive (a_2_), and mixed (a_3_) alleles at the non-HLA DRB1 loci in the general population

a_1m_, a_2m_, a_3m_	=	allelic frequency of dominant (a_1m_), recessive (a_2m_), and mixed (a_3m_) alleles at the non-HLA DRB1 loci in an MS population

F_i_, F	=	unknown "frequency of susceptibility" (see text for definition) at the non-HLA loci in the general population (i = 1, 2,...x). [E(F_i_) = F = h/r)]

F_m_	=	"frequency of susceptibility" at a non-HLA locus in an MS population

h	=	known "frequency of susceptibility" at the HLA DRB1 locus in the general population (equal to the probability of having at least 1 copy of this allele) [h = 2a_h _- (a_h_)^2 ^= 0.24]

h_m_	=	known "frequency of susceptibility" at the HLA DRB1 locus in the MS population (equal to the probability of having at least 1 copy of this allele) [in the UCSF dataset; h_m _= 0.55]

P_a1_, P_a2_, P_a3_	=	probability that a person in the general population has a "susceptible allelic state" (see text for definition) at dominant (P_a1_), recessive (P_a2_), and mixed (P_a3_) non-HLA DRB1 loci. (P_a1 _= P_a2 _= P_a3 _= F = h/r)

P_h1_	=	probability that person with an HLA-negative sibling (not an identical- twin) has at least one copy of the HLA DRB1*1501 allele

P_H_	=	probability that an individual with an affected HLA DRB1*1501 positive sibling has at least one copy this gene

P_A1_, P_A2_, P_A3_	=	probability that an individual will inherit a "susceptible allelic state" given that their sibling is known to be in this state (see text for definition) at dominant (P_a1_), recessive (P_a2_), and mixed (P_a3_) non-HLA DRB1 loci.

**x **(x_1_, x_2_, x_3_)	=	number of non-HLA DRB1susceptibility genetic loci involved in MS (dominant loci = x_1_; recessive loci = x_2_; mixed loci = x_3_). [x_1 _+ x_2 _+ x_3 _= **x**]

P_HM_	=	Probability that an individual (from the general population) is both susceptible to getting MS and carries the HLA DRB1*1501 allele. (if Pt_1 _= Pt_0_; then P_HM _= h_m_)

P_AM_	=	Probability that an individual (from the general population) who is both susceptible to getting MS and is in a susceptible state at a specific non-HLA DRB1 locus. (if Pt_1 _= Pt_0_; then P_AM _= F_m_)

r	=	ratio of the "frequency of susceptibility" at the HLA DRB1 locus to the average "frequency of susceptibility" at other non-HLA DRB1 loci. [r = h/F]

**n **(n_1_, n_2_, n_3_)	=	number of loci in "susceptible allelic states" required for MS to develop (dominant loci = n_1_; recessive loci = n_2_; mixed loci = n_3_). [n_1 _+ n_2 _+ n_3 _= **n**]

P[**n**]	=	probability of an individual in the general population possessing at least **n **loci in a "susceptible allelic state"

C	=	proportion of patients, susceptible to MS, who do not have any copies of the HLA DRB1*1501 allele

P[S]	=	probability that an individual in the general population is susceptible to MS This probability is the same as P(G).

Pt	=	average penetrance of MS phenotype in susceptible patients. Also equal to the proband-wise monozygotic-twin concordance rate (CR_MZ_).

Pt*	=	average penetrance of MS phenotype in susceptible patients, adjusted for the shared intra-uterine and childhood environment of twins. [Pt* = (Pt) (2.9/5.4) = CR_IG_] (See text)

Pt_1_	=	average penetrance of MS phenotype in susceptible patients with at least one copy of the HLA DRB1*1501 allele. Also equal to the proband-wise monozygotic-twin concordance rate (Z_H+_) for this genotype.

Pt_0_	=	average penetrance of MS phenotype in susceptible patients without any copies of the HLA DRB1*1501 allele. Also equal to the proband-wise monozygotic-twin concordance rate (Z_H-_) for this genotype

P(MS_H+_)	=	Probability of recurrence (i.e., the recurrence rate) in a family member of an MS proband who has at least one copy of the HLA DRB1*1501 allele.

P(MS_H-_)	=	Probability of recurrence (i.e., the recurrence rate) in a family member of an MS proband who lacks the HLA DRB1*1501 allele.

P(MS)	=	prevalence of the MS phenotype in the general population (equated to the life-time probability of getting MS)

P(MZ)	=	Probability of the 1^st ^twin of an MZ twin-pair getting MS: It is assumed that: P(MZ) = P(MS)

P(MS | MZ)	=	Conditional probability of getting MS given that your MZ-twin has MS

P(MS | DZ)	=	Conditional probability of getting MS given that your DZ-twin has MS

P(MS | S)	=	Conditional probability of getting MS given that your sibling has MS

P(G)	=	Probability of having any genotype capable of getting MS in response to some environmental exposure

P(E)	=	Probability of receiving any environmental exposure (all factors) sufficient to cause MS in some susceptible individual

CR_MZ_	=	proband-wise monozygotic-twin concordance rate for MS.

CR_IG_	=	proband-wise monozygotic-twin concordance rate for MS adjusted for impact of a shared intrauterine environment. [CR_IG _= (CR_MZ_) (2.9/5.4)] This variable is the identical to (Pt*) but is used for clarity of the text.

**b'**	=	CR_IG_/P(G | IG) = P(MS, G, E | IG)/P(G | IG)

CR_DZ_	=	proband-wise dizygotic-twin concordance rate for MS.

Z_H+_	=	proband-wise monozygotic-twin concordance rate for MS when the proband possesses at least one copy of the HLA DRB1*1501 allele.

Z_H-_	=	proband-wise monozygotic-twin concordance rate for MS when the proband does not possess a copy of the HLA DRB1*1501 allele.

CR_S_	=	concordance rate for the MS phenotype in a non-twin sibling (1^st ^degree)

CR_PC_	=	concordance rate for the MS phenotype in a Parent or Child (1^st ^degree)

CR_AU_	=	concordance rate for the MS phenotype in an Aunt or Uncle (2^nd ^degree)

CR_FC_	=	concordance rate for the MS phenotype in a First Cousin (3^rd ^degree)

**Table 2:** Epidemiological Data Used in the Model^‡^

	Population	Men	Women
Prevalence of MS [P(MS)] *	150	71.4	228.6

MZ twin Concordance (CR_MZ_) *	25%	6.5%	34.0%

Raw% Susceptible [P(MS)]/CR_MZ_)*	0.6%	1.1%	0.7%

Corrected% Susceptible**	1.1%	2.0%	1.3%

% HLA DRB1*1501 (General Population) *	24%		

% HLA DRB1*1501 (MS Population) *	55%		

Homozygous DRB1*1501 (General Population) ^†^	1.6%		

Homozygous DRB1*1501 (MS Population)^†^	10.0%		

‡ For estimated recurrence risks in 1^st^, 2^nd^, and 3^rd ^degree relatives; see Table 12

For estimated recurrence risk in HLA DRB1*1501 positive and negative patients; see Table 3.

* From Canadian Data [11], based on prevalence of 150/100,000 population [16] and split into men and women according to [17]. HLA data: D Sadovnick (personal communication)

** Percent of the population genetically susceptible to susceptible to MS [P(MS)]/CR_MZ_] corrected (see text) for the reported [11] difference in concordance risk for DZ twins (5.4%) and non-twin siblings (2.9%)

† UCSF Database: J Oksenberg (personal communication)

For the purposes of the model, it is supposed that there are (**x**) independent non-HLA DRB1 susceptibility locations (i.e., genetic loci that harbor susceptibility alleles) in addition to the HLA DRB1 location. Each location may contain more than a single gene (i.e. it may be an extended haplotype). Thus, in total, there are (**x **+ 1) susceptibility loci. Moreover, it is supposed that an individual will be genetically susceptible to MS if they have an appropriate combination of some number of these loci in a "susceptible allelic state". If they possess such a combination, however, having more loci in "susceptible allelic states" will not affect the resulting susceptibility despite the fact that this may well affect the penetrance of MS of specific genetic combinations in equivalent environmental circumstances. A "susceptible allelic state" at a specific genetic locus will be said to exist when the genetic configuration at that locus (i.e., its genotype) is such that, either alone or in combination with "susceptible allelic states" at other locations, the configuration increases the likelihood that an individual will develop MS. In order for any specific genetic locus to be considered a susceptibility locus, it must harbor at least one such genotype that is determinative for MS-susceptibility (see below). At any genetic locus, it is possible that more than one "susceptible" genotype might exist. In this case the term "susceptible allelic state" will refer to the presence of any of these genotypes and the term "frequency of susceptibility" at a particular locus will refer to the combined (sum) frequency of these genotypes at this locus in the general population. Any combination of "susceptible allelic states" at the different loci, which results in MS-susceptibility, will be referred to as a "susceptible genetic combination". Any such combination that does not result in MS-susceptibility will be referred to as a "non-susceptible combination".

Naturally, MS does not all have to be genetically-determined. Thus, it may be that certain sets of environmental events are capable of producing MS regardless of the individual's genetic make-up. Moreover, it is also possible that the development of disease may, in some circumstances, be a stochastic process and that the disease is due to only random mechanisms (e.g., unprovoked developmental errors), again independent of an individual's genetic make-up. Although these non-genetic mechanisms seem to account for only a small fraction of the MS cases in the population [10], they may occur occasionally. Nevertheless, the present analysis, however, is focused on the genetic underpinnings of MS.

In this conceptualization, susceptibility is understood to be binary. That is, an individual is either genetically susceptible or they are not. Nevertheless, within the group of susceptible individuals, there may be a wide variation in the likelihood that MS will develop. Such a binary structure is a direct consequence of the notion that there exist "susceptibility alleles", which (either alone or in combination with other alleles) predispose an individual to getting MS. Indeed, the HLA DRB1*1501 allele has been established as just such a susceptibility allele so that the total number of these alleles must be at least one. Presumably, there are many others but, in any case, the total number of susceptibility alleles must also have an upper bound (i.e., certainly not every allele in the human genome can be a susceptibility allele). Therefore, the group of individuals who possess genetic configurations least likely to result in MS (possibly those without any of these alleles), by definition, would be classified as being genetically non-susceptible. Any combination of susceptibility alleles that did not increase the likelihood of MS beyond the rate in this group would also be classified as genetically non-susceptible. By contrast, all genetic combinations that did increase this likelihood would be classified as susceptible combinations. Therefore, the structure is, of necessity, binary.

#### 1. Defining and Representing Genetic Susceptibility

For simplicity of the terminology in this section, we will consider only the situation in which the person is known to be HLA DRB1*1501-negative (i.e., where [F] is the expected probability of an individual having a "susceptible allelic state" at any particular susceptibility locus). The results of this analysis, however, are easily generalized to circumstances in which the HLA DRB1 locus is included (Additional File 1; Appendix S1; Section 4). Defining the combined "frequency of susceptibility" at each of the (**x**) non-HLA DRB1 susceptibility loci as (F_i_) with (i = 1,2,...x), the expected probability that an individual will have any specific combination of **n **independent loci in a susceptible state is:

(1) $$\text{E}[\prod_{\text{i}=1}^{\text{n}}({\text{F}}_{\text{i}})]=\;{\left[\text{E}({\text{F}}_{\text{i}})\right]}^{\text{n}}={\left[\text{F}\right]}^{\text{n}}$$

where E(F_i_) = F for (i = 1,2,...n). Therefore, the use of the average allelic frequency in calculating the probabilities of different specific combinations of the (**x**) loci provides an appropriate representation of the different probabilities.

Because the different genotypes at each of the (**n**) loci can combine separately with any of those at other loci to produce susceptibility, it might appear, at first glance, preferable to consider distinct "susceptibility genotypes" rather than using their combined frequency at each locus (F_i_) as undertaken above. However, if the i^th ^locus has (k_i_) distinct (i.e., mutually exclusive) "susceptibility genotypes" and if we define the population frequency of the j^th ^genotype at the i^th ^locus as (f_ji_), then:

$$\sum_{\text{j}=1}^{{\text{k}}_{\text{i}}}({\text{f}}_{\text{ji}})={\text{F}}_{\text{i}}\;$$

Moreover, considering each genotype individually, the combined probability of the different genotype combinations at the (**n**) loci is:

$$\begin{array}{l} \text{E}[\sum_{{\text{j}}_{1}=1}^{{\text{k}}_{1}}\sum_{{\text{j}}_{2}=1}^{{\text{k}}_{2}}\cdots \sum_{{\text{j}}_{\text{n}}=1}^{{\text{k}}_{\text{n}}}\left({\text{f}}_{{\text{j}}_{1}})({\text{f}}_{{\text{j}}_{2}})\cdots ({\text{f}}_{{\text{j}}_{\text{n}}})\right]\\ \;\;=\text{E}[\sum_{{\text{j}}_{1}=1}^{{\text{k}}_{1}}\left({\text{f}}_{{\text{j}}_{1}}\right)][\sum_{{\text{j}}_{2}=1}^{{\text{k}}_{2}}\left({\text{f}}_{{\text{j}}_{2}}\right)]\cdots [\sum_{{\text{j}}_{\text{n}}=1}^{{\text{k}}_{\text{n}}}\left({\text{f}}_{{\text{j}}_{\text{n}}}\right)]]\\ \;\;=\text{E(}\prod_{\text{i}=1}^{\text{n}}[\sum_{\text{j}=1}^{{\text{k}}_{\text{i}}}\left({\text{f}}_{\text{ji}}\right)])=\text{E}[\prod_{\text{i}=1}^{\text{n}}\left({\text{F}}_{\text{i}}\right)] \end{array}$$

Obviously, this last expression is the same as Equation (1) and, thus, using the collective frequency (F_i_) at each locus to calculate the expected probability of an individual being in a "susceptible allelic state" at each of the **n **loci is equivalent to making the same calculation but considering each distinct genotype separately.

Furthermore, we will let (S) be the set of all genetic combinations at the **x **non-HLA DRB1 susceptibility loci that lead to MS susceptibility. The probability that a person in the general population is a member of this set is defined as either P[S] or P(G) depending upon which terminology is simpler in a given situation. We can partition (S) into disjoint subsets (s_i_), where every combination that is a member of the subset has, within its collection of genotypes at the different loci, at least one group of (s_i_) loci with "susceptibility genotypes" that, by themselves, would result in susceptibility to MS. In addition, no member of the subset can have a group of fewer than (s_i_) loci with "susceptibility genotypes" that, by themselves, would lead to MS susceptibility. The term "by themselves" is used to indicate that a person having this particular combination of "susceptibility genotypes" at the (s_i_) loci is susceptible to getting MS, regardless of the "allelic state" at any other genetic locus.

It is nevertheless still possible (and, indeed, likely) that certain "genotypes" at other genetic loci (either susceptibility or non-susceptibility loci) will influence the penetrance of MS of any such group of (s_i_) genotypes in equivalent environmental circumstances. However, if the "allelic state" at another locus (or other loci) is determinative for the combination of (s_i_) genotypes to result in susceptibility, then this collection of genotypes would belong either to the (s_(i+1)_) subgroup or to an even higher-order subgroup depending upon how many genes were involved and their exact relationship to susceptibility. For example, if a particular genotype at another locus completely nullified the effect of a certain combination of (s_i_) genotypes, then the presence of a "susceptibility genotype" at this other locus (i.e., any genotype other than the one that has this impact) would also be required for the (s_i_) combination to result in susceptibility. In this example, then, the combination of (s_i_) genotypes being considered would actually belong to the (s_(i+1)_) subgroup. It is also possible that a certain "genotypes" or combinations of "genotypes" could both alter the penetrance of some specific (s_i_) combinations and, yet also, be determinative for other combinations. In this case the group of (s_i_) "susceptibility genotypes" would still be in the (s_i_) subgroup as long as the penetrance of their combination with these other "allelic states" remained greater than the penetrance of a non-susceptible combination. Genetic loci with alleles or combinations of alleles at several loci that only modified the penetrance of other combinations (but were never determinative for any combination) would not be included among the **x **non-HLA DRB1 susceptibility loci and, therefore, would not be constrained in the Model. At any specific susceptibility locus, it is possible either that a particular susceptibility gene has more than one susceptibility allele, or that the locus harbors more than one susceptibility gene, or both. Moreover, it is also possible that some of these susceptibility alleles or genes (when they occur in combination with genes or alleles at other loci) will belong to different (s_i_) subgroups. Regardless of the complexity of these interactions, however, every combination of "susceptibility genotypes" at the **x **non-HLA DRB1 susceptibility loci will be uniquely classifiable either into one of the different (s_i_) subgroups or into the group of genetic combinations that do not result in susceptibility to MS.

We will let (y_i_) be the subset of all possible genetic combinations with at least (i) of the (**x**) loci being in a "susceptible allelic state". The probability that an individual genotype is a member of the (y_i_) subset is:

(2) $$\text{P}[{\text{y}}_{\text{i}}]=\sum_{\text{k}=\text{i}}^{\text{x}}[(\text{x})!/(\text{x-k})!(\text{k})!]{\left[\text{F}\right]}^{\text{k}}{\left[1\text{-F}\right]}^{\text{x-k}}$$

If we define (P_i_) as the probability that any member of this (y_i_) subset also belongs to the (s_i_) subset, then the probability of an individual genotype being a member of the (s_i_) subset is:

(3) $$\text{P}[{\text{s}}_{\text{i}}]={\text{P}}_{\text{i}}\cdot \text{P}[{\text{y}}_{\text{i}}]$$

If we define the k^th ^summand of the (y_i_) subset to be (P_k_), and we define (P_ki_) to be the probability that a genetic combination within this k^th ^summand belongs to the (s_i_) subset, then:

$${\text{P}}_{\text{k}}=[(\text{x})!/(\text{x-k})!(\text{k})!]{\left[\text{F}\right]}^{\text{k}}{\left[1\text{-F}\right]}^{\text{x-k}}$$

And, Equation (3) can be re-written as:

(4) P[si]=Pi⋅∑k=ix[(x)!/(x-k)!(k)!][F]k[1-F]x-k=∑k=ix[(Pki)⋅(Pk)]

Although one possibility is that (P_ki _= P_i_) for all values of (k) in Equation (4), this need not be the case. Nevertheless, because, for each (k), the value of (P_k_) is a constant for defined values of (x) and (F), therefore:

$$\sum_{\text{k}=\text{i}}^{\text{x}}\text{E}([{\text{P}}_{\text{ki}}][{\text{P}}_{\text{k}}])=(\sum_{\text{k}=\text{i}}^{\text{x}}\left[{\text{P}}_{\text{k}}])\cdot (\text{E}[{\text{P}}_{\text{ki}}]\right)$$

and, thus, also, that:

(5) E(P[si])=∑k=ixE([Pki][Pk])=E([Pki])⋅(∑k=ix[Pk])=(P[yi])⋅E([Pki])

Consequently, from Equations (3) and (5), it follows that:

$$\text{E}([{\text{P}}_{\text{ki}}]={\text{P}}_{\text{i}}\;;\;\text{for each value of }(\text{k})\text{ and }(\text{i})$$

We will let (d) represent the smallest number of loci (s_(d) _≥ s_1_) that, when combined together in "susceptible allelic states", result in genetic susceptibility to MS (i.e., every individual with fewer than this number of involved loci will not be genetically susceptible). Furthermore, we will define (N) such that (d + N ≤ x) represents the smallest number of loci that, when combined together in "susceptible allelic states" (s_(d+N) _≤ s_x_), will always result in genetic susceptibility (i.e., every individual with this number of loci or more will be genetically susceptible). Thus, (P_i _= 0) for all (s_i _< s_(d)_) and for all (s_i _> s_(d+N)_). In this circumstance, the expected number of loci required to be in a "susceptible allelic state" (E[s_i_]) will be:

$$\text{E}({\text{s}}_{\text{i}})=\sum_{\text{i}=\text{d}}^{\text{d}+\text{N}}\left(\text{i})\cdot (\text{P}\{{\text{s}}_{\text{i}}\}\right)=\sum_{\text{i}=\text{d}}^{\text{d}+\text{N}}\left(\text{i})\cdot {\text{P}}_{\text{i}}\cdot \text{P}[{\text{y}}_{\text{i}}\right]$$

and where:

$$\text{E}(\text{P}[{\text{y}}_{\text{i}}])=\sum_{\text{i}=\text{d}}^{\text{d}+\text{N}}\left(\text{P}[{\text{y}}_{\text{i}}]/\text{N}\right)$$

Naturally, because E(P[y_i_]) is a weighted function using different integer values of [y_i_], it may not correspond to any integer value. Therefore, the value of the integer (**n**) will be assigned such that the condition E(P[y_i_]) ≈ P[n] is most closely approximated. Thus:

$$\text{E}(\text{P}[{\text{y}}_{\text{i}}])\approx \text{P}[\text{n}]=\text{P}[{\text{y}}_{\text{n}}]$$

In this case, one of two conditions will hold. Thus, either:

$$\left(\text{P}[\text{n}+\text{1}]>\text{E}(\text{P}[{\text{y}}_{\text{i}}])>\text{P}[\text{n}]\;or\;(\text{P}[\text{n-1}]<\text{E}(\text{P}[{\text{y}}_{\text{i}}])<\text{P}[\text{n}]\right)$$

depending upon the value of (E(P[y_i_]). Because the large majority of individuals are not susceptible to MS [10], it must be the case that {E(s_i_) > x · F}, where [x · F] is the average number of loci in a "susceptible allelic state" possessed by a random individual in the general population.

In this circumstance, then, it is also the case that:

$$\left(\text{d}\leq \text{E}({\text{s}}_{\text{i}})\leq \text{n}\right)$$

with the exact relationship depending upon the spread (N) of the different genetic configurations that result in MS susceptibility. Therefore, using **n **in the estimating equations will tend to over-estimate the expected number of susceptibility alleles required for susceptibility and will provide an upper bound on the average number involved. In addition, it is noteworthy that:

$$\sum_{\text{i}=0}^{\text{x}}\text{E}({\text{P}}_{\text{i}})=\sum_{\text{i}=\text{d}}^{\text{d}+\text{N}}\text{E}({\text{P}}_{\text{i}})=1$$

This follows because, for every (i < m), the expectation is that (P_i_) of the (y_m_) possible combinations will belong to the (s_i_) subgroup. At (m = d + N), all genetic combinations, including those belonging to (s_(d+N)_) sub-group, will result in susceptibility. Therefore, at this point:

$$\text{E}({\text{P}}_{\left(\text{d}\right)})+\text{E}({\text{P}}_{\left(\text{d}+1\right)})+\cdots +\text{E}({\text{P}}_{\left(\text{d}+\text{N}\right)})=1$$

Also:

$$\text{E}({\text{P}}_{\text{i}})=(\sum_{\text{i}=\text{d}}^{\text{d}+\text{N}}\text{E}({\text{P}}_{\text{i}})/\text{N ; and therefore: N}\cdot {\text{E(P}}_{\text{i}})=1$$

The total probability of an individual being susceptible (P[S]) will be:

$$\text{P}[\text{S}]=\sum_{\text{i}=\text{d}}^{\text{d}+\text{N}}\text{P}\{{\text{s}}_{\text{i}}\}=\text{N}\cdot \text{E}(\text{P}\{{\text{s}}_{\text{i}}\})=\text{N}\cdot \text{E}({\text{P}}_{\text{i}}\cdot \text{P}[{\text{y}}_{\text{i}}])$$

If (P[y_i_]) and (P_i_) are independent (as seems reasonable because no distribution assumptions have been made), then it follows that:

$$\text{E}(\text{P}\{{\text{s}}_{\text{i}}\})=\text{E}({\text{P}}_{\text{i}}\cdot \text{P}[{\text{y}}_{\text{i}}])=\text{E}({\text{P}}_{\text{i}})\cdot \text{E}(\text{P}[{\text{y}}_{\text{i}}])$$

and:

(6) $$\text{P}[\text{S]}=\text{N}\cdot \text{E}({\text{P}}_{\text{i}})\cdot \text{E(P}[{\text{y}}_{\text{i}}])=\text{E}({\text{P[y}}_{\text{i}}])\approx \text{P}[\text{n}]$$

As before, one of two conditions will hold. Thus, either:

(P[n]≥(P[S])>P[n+1]) or (P[n-1]>(P[S])≥P[n])

must be true depending upon the value of (E(P[y_i_]).

Therefore, in these circumstances, assuming that exactly (**n**) loci are involved and that each of (y_n_) genotypes confers susceptibility provides an appropriate representation for the entire distribution of susceptibility genotypes. However, in contrast to (**n**), which tends to overestimate (E[s_i_]), the value of (**x**) derived from the exclusive use of P[**n**] in the calculations will accurately estimate the total number of loci involved in MS susceptibility because P[**n**] is an accurate representation of the total probability of susceptibility, even in circumstances where the susceptibility structure is quite complicated.

The circumstances considered above (i.e., where N · E[P_i_] = 1) are clearly applicable if each locus harbors only a single susceptibility allele. However, in the circumstances where some loci have more than one susceptibility allele for a single susceptibility gene, or where some loci contain more than one susceptibility gene, it is possible that some specific genetic combinations will not result in susceptibility even when all (**x**) loci are in a "susceptible state". In this case, (N · E[P_i_] < 1), and we would need to define an apparent expectation {E'(P[y_i_])]} as:

$$\text{E'}(\text{P}[{\text{y}}_{\text{i}}])=[\text{E}(\text{P}[{\text{y}}_{\text{i}}])]/[\text{N}\cdot \text{E}({\text{P}}_{\text{i}})]$$

so that Equation (6) would become:

$$\text{P}[\text{S}]=\text{N}\cdot \text{E}({\text{P}}_{\text{i}})\cdot \text{E'}(\text{P}[{\text{y}}_{\text{i}}])=\text{E}(\text{P}[{\text{Y}}_{\text{i}}])]\approx \text{P}[\text{n}]$$

Thus, the only impact of this circumstance would be to decrease the estimate of (**n**).

#### 2. Estimating the Proportion of "Genetic" MS in the Population

It is envisioned that the development of MS might occur via different pathogenetic mechanisms, with the life-time probability of developing the MS being defined as P(MS) and with this probability being equated with the prevalence of disease. In the general population, it should be possible to divide MS cases into two broad categories - those cases that developed MS through "genetic" pathways and those that developed MS through "non-genetic" pathways. In this context, the term "genetic" MS is used to indicate that the development of MS has occurred through a pathway requiring both a susceptible genotype (G) and specific environmental events (E). Although it is possible that some cases of MS are purely genetic (i.e., don't require an environmental trigger) this seems likely to be rare. The main reasons for this are that the incidence of MS seems to be increasing in many places around the world, especially in women [10,16,17] and that MS was either unheard of or extremely rare before the 19^th ^century [2,18]. Because purely genetic MS should have remained relatively constant in prevalence over such a short time-period (i.e., human genetics wouldn't be expected to change this quickly), these observations suggest that purely genetic MS is not a major contributor to MS at present.

Rather, the "genetic" form of the disease is envisioned to develop because a genetically susceptible individual (in the sense defined above) experiences a set of environmental events that are sufficient to cause MS given their particular genotype. The probability that an MS case in the general population has developed their MS through this route will be defined as P(MS, G, E), the probability that an individual in the general population is genetically susceptible will be defined as P(G), and the probability that an individual in the general population will experience a sufficient set of environmental events to cause MS in a susceptible person will be defined as P(E). In this formulation, the terms P(G) and P(E) are conceptualized very broadly. Thus, P(G) refers to the probability of possessing *any*genotype that could possibly develop MS through the "genetic route" under *some*set of environmental exposures. Similarly, P(E) refers to the probability of experiencing *any*environmental exposure that could possibly produce MS under *some*selected set of genetic preconditions. As discussed earlier, P(G) is the same as P[S]. Second, the disease may develop because an individual experiences either a special set of environmental events (E**) that are sufficient to cause MS in anyone (i.e., independent of their genetic make-up) or through a set of purely stochastic events (e.g., random, unprovoked, errors during development). The life-time probability that an individual will develop MS by the "genetic" route is termed P(MS, G, E), whereas the corresponding probability for all combined forms of "non-genetic" MS is termed P(MSE).

Thus, the starting equation is:

(7) P(MS)=P(MS,G,E)+ P(MSE)−P(MS,G,E)*P(MSE)=0.0015

where (0.0015) is chosen because it is the mid-point of the estimated prevalence range for MS in Canada (i.e., 0.1-0.2%). Because both P(MSE) and P(MS, G, E) must be (≤ 0.0015), the cross-product term (the probability of getting MS through both routes) is negligible compared to the other two terms and can be ignored. Similarly, as discussed above, any contribution from purely genetic MS (i.e., not requiring an environmental trigger) will also be ignored.

In this circumstance, if we can define the proportion (p) as:

P(MS,G,E)=(p)*P(MS)

so that Equation (7) can be rewritten as:

(8) $$\begin{array}{c} \text{P}\left(\text{MS}\right)=\text{P}\left(\text{MS},\text{G},\text{E}\right)+\text{P}\left(\text{MSE}\right)\\ =\left(\text{p}\right)*\text{P}\left(\text{MS}\right)+\left(\text{1}-\text{p}\right)*\text{P}\left(\text{MS}\right)=0.00\text{15} \end{array}$$

The value of (p) can be estimated from two independent epidemiological observations. However, before considering the implications of these observations, one fundamental assumption is required. If the term P(MZ) is used to represent the life-time probability that the first twin of a monozygotic (MZ) twin-pair will get MS, then the necessary assumption is that this probability is the same as that for any other member of the general population. Stated explicitly, this assumption is that:

P(MZ) =P(MS)

Intuitively, this assumption seems reasonable, especially given the facts that there is no genetic propensity to having MZ twins, that twins not over-represented in MS populations and, finally, that MS is not over-represented in twin populations [11]. These observations are especially compelling considering the fact that almost all cases of concordant MS, whether in MZ twins, in dizygotic (DZ) twins, or in siblings, represent individuals who have developed MS through the genetic route. To demonstrate this, it is clear from above that:

P(MSE)<0.0015

Moreover, because, by definition, P(MSE) is independent of the genetics, then:

P(MSE|S)=P(MSE)

Therefore, the conditional probability that a sibling (S) of an MS proband will get MS can be expressed as:

$$\begin{array}{l} \text{P}\left(\text{MS},\text{E|S}\right)\;=\;0.0\text{29}\\ \;\;\;\;=\text{P}\left(\text{MS},\text{G},\text{E|S}\right)+\text{P}\left(\text{MSE|S}\right)\\ \;\;\;\;<\text{P}(\text{MS},\text{G},\text{E|S})+0.0015 \end{array}$$

and, thus, that:

(9) P(MS,G,E|S) > (0.95)*P(MS,E|S)

This percentage increases to much more than 95% when a more realistic estimate for P(MSE) is used. The same conclusion also applies to concordant MZ twins, in whom the observed concordance rate is considerably higher (0.25; see Table 2). Using this information, together with the fact that the penetrance of MS is essentially the same in patients regardless of weather they carry the HLA DRB1*1501 allele (Table 3), leads to the estimate that (p = 0.92) or that 92% of MS patients have developed their disease through the genetic route [10].

**Table 3:** MS Concordance rates in Monozygotic Twins of HLA DRB1*1501-positive (Z_H+_) and HLA DRB1*1501-negative (Z_H-_) Probands *.

	**Monozygotic Twins of MS Probands**		
	HLA DRB1*1501 Positive	HLA DRB1*1501 Negative	**Totals**
	
**Concordant for MS (C)**	9	11	20
		
**Discordant for MS (D)**	31	42	73
	
**Totals**	40	53	93
		
**Pair-wise Concordance**^**†**^	Z_H+ _= (9/40) = 23%	Z_H- _= (11/53) = 21%	
		
**Proband-wise Concordance**^**††**^	Z_H+ _= 31%	Z_H- _= 29%	
		
**Proband-wise Concordance (Adjusted) **^**†††**^	Z_H+ _= 17%	Z_H- _= 16%	

* Data derived from: Willer et al., 2003 [11]

† Pair-wise rates calculated as (Z = C/(C + D).

**†† **Proband-wise concordance rates calculated as (Z = 2C/(2C + D) adjusted [21] for the overall probability of doubly ascertaining concordant twin-pairs (54%) in the Willer, et al., 2003 [11] study.

††† See Text, Equation (20)

The second observation suggesting that the large majority of MS has developed through the genetic route comes from a population based cohort study in Finland [19]. This study includes 3,083 monozygotic twin-pairs born prior to 1957. These authors reported that in 21 of these pairs, at least one twin had MS and, of these, 10 pairs (3 concordant for MS) agreed to participate in the study. This observation leads to the estimate of:

P(MS,G,E)>(3)(21/10)(1/3,083)=204 per 100,000 population

which, as pointed out previously [10], greatly exceeds the reported prevalence of MS in the general population of Finland [20].

Consequently, both of these observations suggest that the large majority of MS (perhaps all) occurs by the route of genetic susceptibility together with an appropriate environmental exposure and, therefore, suggest that the assumption (p ≈ 1) is a reasonable approximation. In this circumstance:

(10) $$\begin{array}{c} \text{P}\left(\text{MS},\text{E}\right)\approx \text{P}\left(\text{MS},\text{G},\text{E}\right)\\ =\text{P}\left(\text{G}\right)*\text{P}\left(\text{E|G}\right)(\text{P}\left(\text{MS|G},\text{E}\right) \end{array}$$

and:

(11) P(G)=P(MS,G,E)/P(MS,E|G)

or:

(12) P(G)≈P(MS,E)/P(MS,E|G)

each of which follows directly from the definitions of different conditional probabilities. Moreover, even in the circumstance where (p < 1), the estimate for P(MS, G, E) will become smaller so that the estimated proportion of the population who are genetically susceptible will also become smaller.

The conditions leading to Equation (9) are even more applicable to the circumstances of a monozygotic twin of an MS proband.

Therefore:

(13) $${\text{CR}}_{\text{MZ}}=\text{P}\left(\text{MS},\text{E|MZ}\right)\approx \text{P}\left(\text{MS},\text{G},\text{E|MZ}\right)$$

This estimate is referred to as the proband-wise (or case-wise) concordance rate [21]. The relationship between P(MS, E | MZ) and P(MS, E | G) needs to be determined from existing data regarding the impact of a shared intrauterine or early postnatal environment on the likelihood of MS. Fortunately, this environmental impact can be estimated from existing data. Thus, as noted earlier [10,11], the 5.4% recurrence risk for MS in dizygotic twins (CR_DZ_) of an MS proband is substantially higher than the 2.9% recurrence risk in non-twin siblings (CR_S_). Consequently:

(14) $$\begin{array}{c} {\text{CR}}_{\text{DZ}}=\left({\text{CR}}_{\text{DZ}}/{\text{CR}}_{\text{S}}\right)\left({\text{CR}}_{\text{S}}\right)\\ =\left(\text{5}.\text{4}/\text{2}.\text{9}\right)\left({\text{CR}}_{\text{S}}\right)\;=\;\text{1}.\text{86}\left({\text{CR}}_{\text{S}}\right) \end{array}$$

Because several experimental studies have failed to identify any differences in MS risk among adopted individuals, conjugal couples, brothers and sisters of different birth order, and in siblings and half-siblings raised together or apart [22-27], this difference between dizygotic twins and siblings presumably reflects only the impact of a shared intra-uterine or early post-natal environment on MS risk [10]. Therefore, because the P(G) term is the same for both twin and non-twin siblings, then:

(15) $$\begin{array}{c} {\text{CR}}_{\text{DZ}}\approx \text{P}\left(\text{MS},\text{E},\text{G|DZ}\right)\;=\text{P}\left(\text{G|DZ}\right)*\text{P}\left(\text{MS},\text{E|DZ},\text{G}\right)\\ =\;\left(\text{1}.\text{86}\right)*\text{P}\left(\text{G|S}\right)*\text{P}\left(\text{MS},\text{E|S},\text{G}\right) \end{array}$$

Where the different conditional probabilities are defined for the dizygotic (DZ) and sibling (S) cases in the same manner as terms of Equation (13) were defined for the MZ case. Moreover, as discussed above, there seems to be no change in the risk of environmental exposure due to siblings sharing their childhood environment with the MS proband compared to the same risk in siblings growing up in an environment experienced by unrelated individuals in the general population [22-27]. Similarly, there seems to be no change in the risk of environmental exposure due to an unrelated individual sharing their childhood environment with an MS proband compared to their risk growing up in the general population. Thus, it seems that the observed difference in MS risk between non-twin siblings and members of the general population is related to their genetic make-up [i.e., the P(G | S) or P(G | DZ) term] and not their environmental exposure terms. Because:

(16) P(G|S)=P(G|DZ)

Then, from Equation (15):

(17) P(MS,G|DZ,G)=(1.86)*P(MS,E|S,G)

Using this same estimate to adjust for the impact of a shared intra-uterine and early post-natal environment on the identical genotypes (IG) shared by monozygotic twins, using Equation (13), yields:

$$\text{P}\left(\text{MS},\text{G},\text{E|MZ}\right)\;\approx {\text{CR}}_{\text{MZ}}$$

so that:

(18) $${\text{CR}}_{\text{MZ}}\approx \left(\text{1}.\text{86}\right)*\text{P}\left(\text{MS},\text{G},\text{E|IG}\right)$$

Using Equation (18), we can define an adjusted mono-zygotic concordance rate (CR_IG_), removing the intrauterine and early postnatal environmental effects as:

(19) $$\begin{array}{c} {\text{CR}}_{\text{IG}}=\text{P}\left(\text{MS},\text{G},\text{E|IG}\right)\\ ={\text{CR}}_{\text{MZ}}/\text{1}.\text{86}=0.\text{25}/\text{1}.\text{86}=0.\text{134} \end{array}$$

Finally, we note that:

P(MS,G,E|IG)=P(G|IG)*P(MS,E|IG,G)

and we define (**b'**) as:

(20) $$\begin{array}{c} \text{b}’\;={\text{CR}}_{\text{IG}}/\text{P}\left(\text{G|IG}\right)\;=\\ =\text{P}\left(\text{MS},\text{E|IG},\text{G}\right)/\text{P}\left(\text{G|IG}\right) \end{array}$$

However, because we are disregarding any contribution from purely genetic MS (see above), and we are assuming that (p ≈ 1) then:

(21) $$\begin{array}{ccc} \text{P}\left(\text{E|MS}\right)\approx \text{1 }; & \text{and:} & \text{P}\left(\text{G|IG}\right)\;\approx \text{1} \end{array}$$

so that:

P(MS,E)≈P(MS)

In this circumstance:

(22) b’=P(MS|IG,G)

Moreover, because:

P(G)=P(MS,G)/P(MS|G)

so that:

(23) P(G)≤P(MS)/P(MS|G)

If:

$$\begin{array}{ccc} \text{p}\approx \text{1 }; & \text{and},\text{ if also:} & \text{b}’\;=\text{P}\left(\text{MS|IG},\text{G}\right)\approx \text{P}\left(\text{MS|G}\right) \end{array}$$

Then, combining Equations (12), (18), (19), (22), and (23), in this circumstance, yields:

(24) $$\begin{array}{c} \text{P}\left(\text{G}\right)\approx \text{P}\left(\text{MS},\text{E}\right)/\text{b}’\\ \approx \text{P}\left(\text{MS}\right)/\text{b}’=0.00\text{15}/0.\text{134}=\text{1}.\text{1}\% \end{array}$$

#### 3. Estimating the Prevalence of Genetic Susceptibility to MS

Initially, we will divide the various susceptibility genotypes are grouped into two mutually exclusive subsets based on their penetrance. The first group (G1) will be defined as those genotypes with an expected penetrance as high or higher than average, whereas the second group (G2) will be defined as those with an expected penetrance as low or lower than average. [Genotypes with an average penetrance are divided evenly between G1 and G2.] Furthermore, we will define P(G1) and P(G2) as the probabilities of these two different classes of genotype in the general population so that:

(25) P(G)=P(G1)+P(G2)

We will define the (i^th^) susceptibility genotype in (G1) as (G_i_) so that:

$$\begin{array}{c} \text{E}\left\{\text{P}\left(\text{MS},\text{E},{\text{G}}_{\text{i},}\right)\right\}=\text{P}\left(\text{MS},\text{E},\text{G1}\right)\\ =\text{P}\left(\text{G}\right)*\text{P}\left(\text{MS},\text{E|G1}\right) \end{array}$$

Therefore, the average penetrance of the (G1) subset is:

P(MS,E|G1)

Or in circumstances of Equation (21):

P(MS,E|G1)=P(MS|G1)

By the definition of the G1 and G2 subsets, and from Equation (16), it must be the case that:

(26) P(MS|G1)≥P(MS|G2)

Moreover, in order for these two quantities to be equal requires that the variance in the quantity P(MS | G, E) to be zero. However, if the variance is not zero, then, in moving from the susceptible population to the MS population (or to the MZ twin population where one twin is known to have MS), there must be an enrichment of the more penetrant (G1) genotypes in comparison to the less penetrant (G2) genotypes. Therefore, it must be the case that:

(27) b’=P(MS|IG,G)≥P(MS|G)

Consequently, from Equations (23) and (27), it must be the case that:

(28) P(G)≥P(MS,G)/b’

When:

$$\begin{array}{ccc} \text{p}=\text{1} & \text{then}: & \text{P}\left(\text{G1}\right)=\text{P}\left(\text{G}\right) \end{array}$$

all genotypes have the same penetrance and, therefore:

P(G)=P(MS,G)/b’

so that:

(29) b’ = P(MS,G)/ P(G) = P(MS|G) = P(MS|IG,G)

If we define (p') as:

(30) (p')*P(G) = P(G1)

so that:

(31) (1–p')*P(G) = P(G2)

We note that:

(32) $$\begin{array}{c} \text{P}\left(\text{MS},\text{G}\right)/\text{P}\left(\text{G1}\right)\;=\text{P}\left(\text{MS},\text{G}\right)/\left(\text{p'}\right)\text{P}\left(\text{G}\right)\;\\ \;=\text{P}\left(\text{MS|G}\right)\;/\text{p'} \end{array}$$

Because, from Equation (28), at (p' = 1):

P(MS,G)/P(G1) = b’

It is apparent from Equation (31) that, as (p') decreases, the value of {P(MS, G)/P(G1)} increases.

Therefore:

(33) P(MS,G)/P(G1) ≥ b’

and:

P(G1) = (p’)*P(G) ≤ P(MS,G)/b’

or:

(34) P(G)  ≤  {P(MS,G)/b’}/(p’)

A similar analysis for the (G2) subset leads to the conclusion that:

(35) P(G) ≤ {P(MS,G)/b’}/(1–p’)

This imposes two simultaneous constraints of the possible value that P(G) can take. Moreover, it must be the case that one of the following three statements is true:

$$\begin{array}{cccc} \text{p}’\;=\;0.\text{5}; & \text{p}’>0.\text{5};\; & \text{or}:\; & \left(\text{1}-\text{p}’\right)\;>\;0.\text{5} \end{array}$$

Therefore, these two constraints require that:

(36) $$\begin{array}{c} \text{P}\left(\text{G}\right)\;\leq \;\left(\text{2}\right)*\left\{\text{P}\left(\text{MS},\text{G}\right)/\text{b}’\right\}\;\\ \leq \;\left(\text{2}\right)*\left\{\text{P}\left(\text{MS}\right)/\text{b}’\right\}\;=\;\text{2}.\text{2}\% \end{array}$$

Consequently, based on the epidemiological evidence from Canada, at most, only 2.2% of the population is genetically susceptible to getting MS under any circumstance.

#### 4. Total Number of Susceptibility Loci and Number Necessary

As discussed and developed in Section 1, for the purpose calculating the expected occurrence of MS in different circumstances, the complex genetic susceptibility structure envisioned can be appropriately represented using the expected "frequency of susceptibility" and, also, by considering that any combination of a fixed number (**n**) of susceptibility loci (in a "susceptible allelic state") leads to susceptibility. The estimated prevalence of HLA DRB1*1501-positivity (i.e., the likelihood of an individual possessing at least one copy of this allele) in the general population of Canada is approximately 24% whereas, in the MS population, this number is increased to approximately 55% (Table 3). These numbers are quite similar to other reports from other North American and northern European populations [2,11-14] and are very similar to the large sample collected at UCSF (J Oksenberg, personal communication). The HLA DRB1*1501 allele (like many other alleles) exists on an extended haplotype in linkage disequilibrium in these populations so that distinction of one gene from another on the haplotype has proven difficult [28-30]. Using the Hardy-Weinberg equilibrium equation for the general population, the probability (h) that any individual carries at least 1 copy of the HLA DRB1*1501 extended haplotype is:

(37) $$\;\text{h}\;=\;{\text{2a}}_{\text{h}}\left(\text{1}-{\text{a}}_{\text{h}}\right)+{\text{a}}_{\text{h}}{}^{\text{2}}\;=\;{\text{2a}}_{\text{h}}–{\text{a}}_{\text{h}}{}^{\text{2}}\;=\;0.\text{24}$$

From this expression, the allelic frequency (a_h_) can be calculated and is (a_h _= 0.128).

At each genetic locus, the "frequency of susceptibility" represents the probability that a random individual in the general population will be in a "susceptible allelic state" at a particular genetic locus. Definitions for the different types of susceptibility loci (dominant, recessive, or mixed) are presented in the Additional File 1 (Appendix S1; Section 1). In the case of a "dominant" susceptibility allele (or alleles), the "frequency of susceptibility" is the probability that an individual in the general population has at least one copy of this allele (or these alleles). For simplicity, at the HLA DRB1 location, the "frequency of susceptibility" (as indicated above) will be approximated by the known value of (h). This, of course, ignores contributions to susceptibility from other HLA DRB1 alleles [12]. However, as a rough approximation, this simplification seems reasonable. In the case of a "recessive" susceptibility allele (or alleles), by contrast, the "frequency of susceptibility" is the probability that an individual in the general population has two copies of this allele. If there are two or more different recessive alleles, the heterozygous state (i.e., the possession of two different "recessive" alleles) may or may not confer susceptibility (see Additional File 1; Appendix S1; section 5). In the case of "mixed dominance" alleles, the "frequency of susceptibility" will be the sum of these "dominant" and "recessive" frequencies. We will let (F_i_) be the "frequency of susceptibility" at the i^th ^non-HLA DRB1 location and we will define (**r**) as an unknown positive constant such that the mathematical expectation (E) of (F_i_) is:

(38) $$\text{E}\left({\text{F}}_{\text{i}}\right)\;=\;\text{F}\;=\;\text{h}\left(\text{1}/\text{r}\right)\;$$

Thus, the constant (**r**) relates the expected (but unknown) "frequency of susceptibility" at these non-HLA DRB1 locations to the known "frequency of susceptibility" at the HLA DRB1 locus and (F) reflects the average of these individual "frequencies of susceptibility" for the different susceptibility loci. The constant (**r**) is of no importance in itself. Rather, it is used as a convenience to permit the expected "frequency of susceptibility" to be varied over the entire range of possible values (i.e., from near 0 to near 1) and expressed in terms of the known value of (h). Naturally, because each locus (haplotype) will typically consist of several genes, it is possible that there may be more than one susceptibility gene at any one locus and it is also possible that there may be more than one susceptibility allele for any single gene within the locus. The impact of both these circumstances is considered in the Additional File 1 (Appendix S1; Section 5). For developing the model initially, however, in both the "dominant' and "recessive" cases, the only the circumstance that will be considered is that in which each genetic locus harbors only a single susceptibility gene and that gene has only a single susceptibility allele. In the "mixed dominance" case, each locus will be presumed to have just two susceptibility alleles. As shown in the Additional File 1 (Appendix S1; Section 5), the derived model is broadly applicable and, importantly, because the "frequency of susceptibility" is completely independent of these complexities, the predictions and conclusions derived from the model regarding the expected prevalence of MS in the general population are not altered by the specific genetic configuration of each locus.

In the case of a "dominant" allele at any particular locus, the probability that a random individual in the population will be in a "susceptible allelic state" (P_a1_) at this location will be:

(39) $${\text{P}}_{\text{a1}}\;=\;\text{2}\left({\text{a}}_{\text{1}}\right)-{\left({\text{a}}_{\text{1}}\right)}^{\text{2}}\;=\;\left(\text{h}/\text{r}\right)$$

Therefore, the expected allelic frequency, in this circumstance, will be [a_1 _= 1 - (1 - h/r)^1/2^].

By contrast, in the case of a "recessive" allelic trait at any particular locus, the probability that a random individual in the population will be in a "susceptible allelic state" (P_a2_) at this location will be:

(40) $${\text{P}}_{\text{a2}}\;=\;{\left({\text{a}}_{\text{2}}\right)}^{\text{2}}\;=\;\left(\text{h}/\text{r}\right)$$

Therefore, the expected allelic frequency, in this circumstance, will be [a_2 _= (h/r)^1/2^].

For a "mixed dominance" allelic trait at a particular locus, the probability that a random individual in the population will be in a susceptible allelic state (P_a3_) at this location will be:

(41) $$\begin{array}{c} {\text{P}}_{\text{a3}}={\left({\text{a}}_{\text{3}}\right)}^{\text{2}}+\text{2}\left(\text{1}-{\text{2a}}_{\text{3}}\right)\left({\text{a}}_{\text{3}}\right)+\text{2}\left({\text{a}}_{\text{3}}\right)\left({\text{a}}_{\text{3}}\right)+{\left({\text{a}}_{\text{3}}\right)}^{\text{2}}\\ =\left[\text{2}\left({\text{a}}_{\text{3}}\right)-{\left({\text{a}}_{\text{3}}\right)}^{\text{2}}\right]+\left[{\left({\text{a}}_{\text{3}}\right)}^{\text{2}}\right]={\text{2a}}_{\text{3}}=\left(\text{h}/\text{r}\right) \end{array}$$

and the apparent (mixed) allelic frequency is: [a_3 _= (h/r)/2]. In the limit, as (r→∞), (a_1 _= a_3_).

### Estimating the Number of MS Susceptibility Loci

There are several epidemiological observations that are of relevance to the possible genetic arrangements that might produce MS susceptibility. First, as noted earlier, 45% of MS patients in Canada do not carry the HLA DRB1*1501 allele and, therefore, do not have HLA DRB1*1501-associated MS. Second, the proband-wise concordance rate for the MS phenotype in identical-twin siblings (CR_MZ_) in Canada is approximately 25% [11], which provides an estimate of the average penetrance (Pt) of the MS phenotype (with a shared intra-uterine environment) for all susceptibility genotypes. The penetrance of the HLA DRB1*1501 genotype (Pt_1_) relative to the expected (i.e., average) penetrance of the other genotypes (Pt_0_) is not firmly established, although an estimate can be made from the published data out of Canada [11] and, in this dataset, Pt_1 _and Pt_0 _seem to be very similar (Table 3). Third, the average concordance rate in Canada for non-twin siblings of an MS proband is approximately 2.9% [11]. By contrast, this same rate for dizygotic twins in Canada is 5.4% [11], suggesting that the intrauterine environment is important to MS pathogenesis [10]. As discussed earlier, this requires that the expected penetrance for MS in non-twin siblings needs to be down-weighted compared to the estimate taken from identical-twin siblings (i.e., CR _MZ(S) _= Pt* = [Pt][2.9/5.4] = 0.134). Fourth, the prevalence of MS in the general populations of Canada is approximately 0.1-0.2% [2,11,16]. A similar prevalence estimate pertains to northern European and other North American populations [2,11-15].

Each of these epidemiological observations places constraints on the genetic possibilities. For example, based only on the facts that 55% of MS patients have at least one copy of the HLA DRB1*1501 allele and a proband-wise identical-twin concordance rate of 25%, it is clear that there must be susceptibility alleles at 3 or more different genetic loci and, moreover, that a "susceptible allelic state" must be present at 2 or more (but not all) of these locations in order to produce susceptibility to MS. Thus, if there were only one susceptibility locus (or if there were two loci and both were necessary), then every MS patient would be HLA DRB1*1501-positive (or they would possess one or more of the minor susceptibility alleles at this location). Furthermore, if there were two (or more) loci and a "susceptible allelic state" at only one locus were necessary, then it would be difficult to explain a 25% penetrance for MS in identical-twins in circumstances where only about 1 in 300 HLA DRB1*1501-positive individuals in the general population will ever develop MS and where only about 2.6% of these individuals are even susceptible in the first place (Additional File 1; Appendix S1; Section 4).

Further constraints are also imposed by these epidemiological conditions. Thus, consider the circumstance in which susceptibility to MS is conferred when an individual has a "susceptible allelic state" at any 2 (or more) of the (**x **+ 1) susceptibility loci (haplotypes). For an individual who is known to be susceptible to MS, assuming independence of the haplotypes and using the average "frequency of susceptibility" (F = h/r) at the non-HLA DRB1 loci, the probability that this person will be HLA DRB1*1501-negative can be calculated. Thus, the probability of randomly picking the first of these two loci (which must be in "susceptible allelic states") to be at a non-HLA DRB1 locus is:

(xh/r)/[(xh/r)+(h)] = [x/(x+r)]

The probability of picking the second locus also to be a non-HLA DRB1 locus is:

[(x–1)/(x+r–1)]

The probability that the individual is also HLA DRB1*1501-negative is (1 - h = 0.76). Therefore, the probability that all three of these conditions hold simultaneously is:

[x/(x+r)][(x–1)/(x+r–1)][0.76] = C 

where (C = 1 - h_m_) represents the actual proportion of susceptible individuals who do not carry at least one copy of the HLA DRB1*1501 allele. In the circumstance where (Pt_1 _= Pt_0_), the observed proportion of HLA DRB1*1501-negative MS patients (C_obs _= 0.45) will be equal to the true value of C (see Additional File 1; Appendix S1; Section 2). In this case, then:

(42) [x/(x+r)][(x–1)/(x+r–1 )]  = C*

where:

C* = C/(0.76)=(0.45)/(0.76) = 0.59

In general (for **n **susceptibility loci) the probability of being HLA DRB1*1501-negative is:

[x/(r+x)][(x–1)/(x+r–1)]...[(x–n+1)/(x+r–n+1)] = C*

or, equivalently:

(43) [(x)(x–1)...(x–n+1)]/[(x+r)(x+r−1)...(x+r–n+1)] = C*

As shown in Additional File 1 (Appendix S1; Section 2), there is a relationship between the values that **n**, **r**, and **x **can take in Equation (43). Thus, by solving the different **n**-degree polynomials (in **x**) for various values of **r**, **n**, and (Pt_1_/Pt_0_), these relationships permit the calculation of the number of non-HLA DRB1 susceptibility loci that are required to support a specific number of involved loci (i.e., that harbor susceptibility alleles) as being necessary for MS to develop under specific conditions.

For example, consider the circumstances, in which (**r **= 2), (**n **= 4) and (Pt_1_/Pt_0 _= 1). In this case, from Equation (43), we get:

[x/(x+2)][(x−1)/(x+1)][(x−2)/x][(x−3)/(x−1)]  = 0.59

or:

$$\begin{array}{ccc} {\text{x}}^{\text{2}}–\text{5x}+\text{6}\;=\;0.\text{59}\left({\text{x}}^{\text{2}}+\text{3x}+\text{2}\right) & \text{so that}: & \text{x}\;=\;\text{15}.\text{9} \end{array}$$

Thus, for this particular genetic configuration, there must be 15 or 16 non-HLA DRB1 susceptibility loci (haplotypes). The solutions to some of these equations at specific values of (**n**), (**r**), and (Pt_1_/Pt_0 _= 1) are presented in Tables 4 and 5.

**Table 4:** (4a) The Estimated total number of non-HLA susceptibility genes (**x**) based on the number of such genes necessary for MS to develop (**n**), and the frequency of susceptibility at the non-HLA susceptibility loci in the population (Pt_0 _= Pt_1_).

	Number of Susceptibility Genes Required (n)						
	**5**	**10**	**11**	**12**	**13**	**14**	**15**

**Frequency of Susceptibility (r)**	**Estimated Total Number of non-HLA Susceptibility Genes (x)**						

r = 0.25	5	11	12	13	14-15	15-16	16-17

r = 0.33	6	12	13-14	14-15	16	17-18	18-19

r = 0.5	7	14-15	16-17	17-19	19-20	21-22	22-23

r = 1	11-12	23-25	25-27	28-30	30-32	33-35	**35-37**

r = 2	18-22	40-44	45-48	49-53	**53-57**	**58-61**	**62-66**

r = 4	35-42	75-83	**83-91**	**92-99**	**100-107**	**108-116**	**116-124**

r = 8	67-82	146-161	**162-177**	**177-193**	**193-208**	**209-224**	**225-240**

r = 16	131-162	287-317	**318-348**	**349-379**	**380-410**	**411-441**	**442-472**

**Frequency of Susceptibility (r)**	**Estimated Prevalence (Target = 0.1 - 0.2%)**						

r = 0.25	11.5%	12.7%	12.7%	12.4%	12.3 - 13.2%	12.1 - 13.2%	12.0 - 13.1%

r = 0.33	7.5%	5.2%	4.4 - 6.7%	3.7 - 5.9%	5.1%	4.4 - 6.4%	3.8 - 5.7%

r = 0.5	3.5%	1.2 - 2.0%	1.4 - 2.1%	0.9 - 2.2%	1.1 - 1.6%	1.2 - 1.7%	0.8 - 1.2%

r = 1	1.8 - 2.4%	0.6 - 1.0%	0.4 - 0.7%	0.4 - 0.7%	0.28 - 0.48%	0.27 - 0.45%	**0.19 - 0.33%**

r = 2	1.1 - 2.0%	0.3 - 0.6%	0.28 - 0.43%	0.21 - 0.37%	**0.16 - 0.28%**	**0.14 - 0.21%**	**0.10 - 0.18%**

r = 4	1.0 - 1.9%	0.26 - 0.47%	**0.20 - 0.36%**	**0.16 - 0.27%**	**0.12 - 0.21%**	**0.10 - 0.17%**	**0.07 - 0.13%**

r = 8	1.0 - 1.8%	0.24 - 0.42%	**0.18 - 0.32%**	**0.14 - 0.25%**	**0.11 - 0.19%**	**0.08 - 0.14%**	**0.06 - 0.11%**

r = 16	0.9 - 1.7%	0.23 - 0.40%	**0.17 - 0.30%**	**0.13 - 0.23%**	**0.09 - 0.18%**	**0.08 - 0.13%**	**0.06 - 0.10%**

**limit**	**1.27%**	**0.29%**	**0.22%**	**0.17%**	**0.13%**	**0.10%**	**0.07%**

Optimal solutions underlined.

**Table 5:** (4b) The total number of non-HLA susceptibility genes (**x**) based on the number of such genes necessary for MS to develop (**n**), and the frequency of susceptibility at the non-HLA susceptibility loci in the population (Pt_0 _= Pt_1_).

	Total Number Susceptibility Genes Required (n)						
	**16**	**17**	**18**	**30**	**40**	**50**	**60**

**Frequency of Susceptibility (r)**	**Estimated Total Number of non-HLA Susceptibility Genes (x)**						

r = 0.25	18	19	20 - 21	33-35	44-46	56-58	67-71

r = 0.33	19-20	21	22 - 23	36-38	49-51	61-64	74-80

r = 0.5	24-25	25 - 26	27 - 28	**44-47**	**60-62**	75-78	**91-101**

r = 1	**37-39**	**40-42**	**42 - 44**	71-75	95-100	120-124	144-166

r = 2	**66-70**	**71-74**	75 - 79	125-133	168-176	212-220	255-300

r = 4	**124-132**	132-140	140 - 148	234-250	316-331	397-413	478-569

r = 8	240-256	256 - 271	272 - 287	453-484	611-642	769-800	926-1109

r = 16	473-503	504 - 535	535 - 566	892-953	1202-1264	1512-1574	1823-2000

**Frequency of Susceptibility (r)**	**Estimated Prevalence (Target = 0.1 - 0.2%)**						

r = 0.25	13.1%	13.1%	13.0 - 13.3%	13.0 - 13.4%	13.1 - 13.4%	13.4%	13.4%

r = 0.33	3.2 - 5.0%	4.4%	3.8 - 5.6%	1.7 - 3.5%	1.6 -3.4%	1.0 - 3.1%	1.0 - 5.4%

r = 0.5	0.9 - 1.3%	0.6 - 1.0%	0.7 - 1.0%	**0.10 - 0.35%**	**0.05 - 0.11%**	0.01 - 0.05%	**0.00 - 0.20%**

r = 1	**0.14 - 0.24%**	**0.14 - 0.23%**	**0.10 - 0.17%**	0.00 - 0.03%	0.00 - 0.00%	0.00 - 0.00%	0.00 - 0.00%

r = 2	**0.07 - 0.14%**	**0.07 - 0.10%**	0.05 - 0.09%	0.00 - 0.00%	0.00 - 0.00%	0.00 - 0.00%	0.00 - 0.00%

r = 4	**0.06 - 0.10%**	0.04 - 0.08%	0.03 - 0.06%	0.00 - 0.00%	0.00 - 0.00%	0.00 - 0.00%	0.00 - 0.00%

r = 8	0.05 - 0.09%	0.04 - 0.07%	0.03 - 0.05%	0.00 - 0.00%	0.00 - 0.00%	0.00 - 0.00%	0.00 - 0.00%

r = 16	0.05 - 0.08%	0.03 - 0.06%	0.03 - 0.05%	0.00 - 0.00%	0.00 - 0.00%	0.00 - 0.00%	0.00 - 0.00%

**limit**	**0.06%**	**0.04%**	**0.03%**	**0.00%**	**0.00%**	**0.00%**	**0.00%**

### Estimating the Concordance in a Non-twin Sibling (CR_S_)

There are also other constraints on the system. First, the expected concordance for MS in non-twin siblings of an MS proband is:

$$\text{E}\left({\text{CR}}_{\text{S}}\right)\;=\;\text{E}\left[\left(\text{1}-\text{C}\right)\text{*}\text{P}\left({\text{MS}}_{\text{H}+}\right)\;+\left(\text{C}\right)\text{*}\text{P}\left({\text{MS}}_{\text{H}-}\right)\right]\;=\;0.0\text{29}–0.0\text{38}$$

where P(MS_H+_) and P(MS_H-_) are the respective probabilities that a non-twin sibling of an HLA DRB1*1501-positive or HLA DRB1*1501-negative proband is concordant for MS. Second, the average penetrance for the different genotypes that confer MS susceptibility is estimated by the proband-wise concordance rate for identical-twin siblings of an MS proband and (in northern North America and northern Europe) is equal to:

(44) $$\text{Pt}\;=\;\text{E}\left[\left(\text{1}-\text{C}\right){\text{Pt}}_{\text{1}}\;+\;\left(\text{C}\right){\text{Pt}}_{0}\right]\;=\;0.\text{25}$$

As discussed in Additional File 1 (Appendix S1; Section 2), this implies that:

$$\left({\text{Pt}}_{\text{1}}\leq \text{1}.{\text{8 Pt}}_{0}\right),\;\left({\text{Pt}}_{\text{1}}\leq \;0.\text{32}\right),\text{ and }\left({\text{Pt}}_{0}\geq 0.\text{18}\right)$$

However, these estimated penetrance values (Pt_0 _and Pt_1_) include both the actual penetrance of the genotype under normal circumstances plus the environmental impact of sharing the same intrauterine environment. As noted earlier [11], because the estimated fraternal twin concordance rate (5.4%) is greater that the corresponding rate for non-twin siblings (2.9%), the estimated penetrance for an identical nuclear genome, outside of a shared intrauterine environment, (Pt_0_* and Pt_1_*) needs to be modified such that Equation (44) becomes:

$$\;\text{Pt}\text{*}\;=\;\text{E}\left[\left(\text{1}-\text{C}\right){\text{Pt}}_{\text{1}}\text{*}+\;\left(\text{C}\right){\text{Pt}}_{0}\text{*}\right]\;=\;\left(0.\text{25}\right)\left(0.0\text{29}/0.0\text{54}\right)\;=\;0.\text{134}$$

Third, the prevalence of MS in the general population is estimated to be:

P(MS) = 0.1–0.2%

From these constraints, after assigning specific values for (**n**, **x**, and **r**), P(MS) can be calculated as:

$$\begin{array}{l} \text{P}\left(\text{MS}\right)=(\sum_{\text{i}=0}^{1}\left[({\text{Pt}}_{\text{i}}*)][\left(\text{1}\right)!/\left(\text{i}\right)!\left(\text{1}-\text{i}\right)!][{\left(\text{h}\right)}^{\text{i}}{\left(\text{1}-\text{h}\right)}^{\text{1}-\text{i}}]\right)*\\ \;[\text{1}\;-(\sum_{\text{j}=0}^{\text{n}-\text{i}-1}\left[\left({\text{x}}_{\text{1}}\right)!/\left({\text{x}}_{\text{1}}-\text{j}\right)!\left(\text{j}\right)!\right]\left[{\left({\text{P}}_{\text{a1}}\right)}^{\text{j}}{\left(\text{1}-{\text{P}}_{\text{a1}}\right)}^{\text{x1}-\text{j}}\right])*\\ \;\;(\sum_{\text{k}=0}^{\text{n}-\text{i}-\text{j}-1}\left[\left({\text{x}}_{\text{2}}\right)!/\left({\text{x}}_{\text{2}}-\text{k}\right)!\left(\text{k}\right)!\right]\left[{\left({\text{P}}_{\text{a2}}\right)}^{\text{k}}{\left(\text{1}-{\text{P}}_{\text{a2}}\right)}^{\text{x2}-\text{k}}\right])*\\ \;\;\;(\sum_{\text{m}=0}^{\text{n}-\text{i}-\text{j}-\text{k}-1}\;\;\left[\left({\text{x}}_{\text{3}}\right)!/\left({\text{x}}_{\text{3}}-\text{m}\right)!\left(\text{m}\right)!\right]\left[{\left({\text{P}}_{\text{a3}}\right)}^{\text{m}}{\left(\text{1}-{\text{P}}_{\text{a2}}\right)}^{\text{x3}-\text{m}}\right]))] \end{array}$$

If (n - i - 1 > x_1_), (n - i - j - 1 > x_2_), or (n - i - j - k - 1 > x_3_), then all further entries of the summations involving (j > x_1_), (k > x_2_), or (m > x_3_) are set to [0].

Because binomial distributions [B(n, p)] have the property that, if x_1 _and x_2 _are independent binary variables with distributions B(n_1_, p) and B(n_2_, p) respectively, then (x_1 _+ x_2_) is also binomial with the distribution B(n_1 _+ n_2_, p).

Thus, because, by the conditions of the model, (P_a1 _= P_a2 _= P_a3 _= h/r), and letting (x = x_1 _+ x_2 _+ x_3_), this equation can be rewritten as:

(45) $$\begin{array}{l} \text{P}\left(\text{MS}\right)=(\sum_{\text{i}=0}^{1}\left[(\text{Pt}\text{*})][\left(\text{1}\right)!/\left(\text{i}\right)!\left(\text{1}-\text{i}\right)!][{\left(\text{h}\right)}^{\text{i}}{\left(\text{1}-\text{h}\right)}^{\text{1}-\text{i}}]\right)*\\ \;[\text{1}\;-\;(\sum_{\text{j}}^{\text{n}-\text{i}-1}\;\;\left[\left(\text{x}\right)!/\left(\text{x}-\text{j}\right)!\left(\text{j}\right)!\right]\left[{\left(\text{h}/\text{r}\right)}^{\text{j}}{\left(\text{1}-\text{h}/\text{r}\right)}^{\text{x}-\text{j}}\right])]\; \end{array}$$

In a similar manner, it is also possible to estimate the concordance in non-twin siblings of an MS proband. Thus, because the proband is known to have MS, one or the other of the biological parents (taken together) must possess at least the requisite number and combination of loci (haplotypes) that are in a "susceptible allelic state" for MS to develop under proper environmental circumstances. Assuming independence of the loci (haplotypes), the probability that the other sibling will inherit the "susceptible allelic state" at any specific locus from the parent who has it can be calculated. For example, the sibling of an HLA DRB1*1501-positive proband, has a 50% chance of inheriting the HLA DRB1*1501 allele from the parent who has it. This sibling also has a 50% chance of receiving the other allele from this same parent (which is of unknown status). This second allele has the population probability of being HLA DRB1*1501 (i.e. a_h _= 0.128), so that the total probability of getting an HLA DRB1*1501 allele from this parent is [0.5 + (0.5)a_h_] or 56.4%. In addition their second allele (from the other parent) at this locus (also of unknown status) will have a 12.8% chance of being HLA DRB1*1501. The sum of these probabilities (less the probability of being homozygous for HLA DRB1*1501) will be the chance that the second sibling will have at least one copy of the HLA DRB1*1501 allele, given that the proband sibling is HLA DRB1*1501-positive. Thus, this probability (P_H_) is:

$$\begin{array}{c} {\text{P}}_{\text{H}}\;=\;\left(0.\text{5}\right)\left(\text{1}\;+\;{\text{a}}_{\text{h}}\right)\;+\;\left({\text{a}}_{\text{h}}\right)\;–\;\left(0.\text{5}\right)\left(\text{1}\;+\;{\text{a}}_{\text{h}}\right)\left({\text{ a}}_{\text{h}}\right)\\ =\;\left(0.\text{5}\right)\left(\text{1}+{\text{2a}}_{\text{h}}–{\text{a}}_{\text{h}}{}^{\text{2}}\right)\;=\;\text{62}.0\%\; \end{array}$$

This is the same for all "dominant" true-susceptibility alleles except that the non-HLA DRB1*1501 allelic frequency $\left[a_{1}=1-{\left(1-h/r\right)}^{1/2}\right]$ is substituted for (a_h_). Thus, this probability (P_A1_) will be:

$${\text{P}}_{\text{A1}}\;=\;\left[0.\text{5}\right]\left[\text{1}+{\text{2a}}_{\text{1}}–{\text{a}}_{\text{1}}{}^{\text{2}}\right]$$

In the case of either a "recessive" true-susceptibility alleles or an absent "dominant" protective allele, each parent must possess at least one copy of the "susceptibility" allele so that the probability (P_A2_) of inheriting two such alleles, based on the allelic frequency [a_2 _= (h/r)^1/2^], would be:

$${\text{P}}_{\text{A2}}\;=\;{\left[\left(0.\text{5}\right)\left(\text{1}+{\text{a}}_{\text{2}}\right)\right]}^{\text{2}}\;=\;\left[0.\text{25}\right]{\left[\text{1}+{\text{a}}_{\text{2}}\right]}^{\text{2}}$$

In the case of "mixed dominance" alleles, the "known" allelic state in the proband sibling could be either the "dominant" or the "recessive" state so that the probability (P_A3_) of inheriting this same state will be a mixture of these two cases. Thus, the probability that the state will be "recessive" is:

$$\left({\text{a}}_{\text{3}}{}^{\text{2}}\right)/\left[\left({\text{a}}_{\text{3}}{}^{\text{2}}\right)+\left({\text{2a}}_{\text{3}}–{\text{a}}_{\text{3}}{}^{\text{2}}\right)\right]\;=\;\left({\text{a}}_{\text{3}}\right)/\text{2}$$

and, therefore, the probability of the state being "dominant" is [1-(a_3_)/2]. In the case this probability (P_A3_) would be:

$$\begin{array}{c} {\text{P}}_{\text{A3}}\;=\;\left[\text{1}–\left({\text{a}}_{\text{3}}\right)/\text{2}\right]\left[{\text{P}}_{\text{A1}}\right]\;+\;\left[\left({\text{a}}_{\text{3}}\right)/\text{2}\right]\left[{\text{P}}_{\text{A2}}\right]\\ =\;\left[\text{1}–\left({\text{a}}_{\text{3}}\right)/\text{2}\right]\left[0.\text{5}\right]\left[\text{1}+\text{2}\left({\text{a}}_{\text{3}}\right)-{\left({\text{a}}_{\text{3}}\right)}^{\text{2}}\right]+\left[\left({\text{a}}_{\text{3}}\right)/\text{2}\right]\left[0.\text{25}\right]{\left[\text{1}+{\text{a}}_{\text{3}}\right]}^{\text{2}}\\ =\;\left[0.\text{5}\right)]\left[\text{1}+\text{2}\left({\text{a}}_{\text{3}}\right)–\text{2}{\left({\text{a}}_{\text{3}}\right)}^{\text{2}}+{\left({\text{a}}_{\text{3}}\right)}^{\text{3}}\right] \end{array}$$

Where the allelic frequency is [a_3 _= (h/r)/2].

Because the proband (affected) sibling is only required to be in a "susceptible allelic state" at (**n**) of the (**x **+ 1) susceptibility loci, the allelic status at the other (**x **+ 1 - **n**) loci is not constrained by having an affected sibling and, therefore, the probability that these other loci are in a "susceptible state" will reflect their population "frequency of susceptibility". By contrast, if the proband is HLA DRB1*1501-negative, because it is known that 2 of the 4 parental alleles can not be HLA DRB1*1501, the probability that a sibling (not an identical-twin) is HLA DRB*1501-positive (P_h1_) will be about once (not twice) the population allelic frequency. Thus:

$$\begin{array}{c} {\text{P}}_{\text{h1}}\;=\;\left(0.\text{5}\right)\left({\text{a}}_{\text{h}}\right)+\left(0.\text{5}\right)\left({\text{a}}_{\text{h}}\right)–{\left[\left(0.\text{5}\right)\left({\text{a}}_{\text{h}}\right)\right]}^{\text{2}}\\ \;=\;\left[{\text{a}}_{\text{h}}–0.\text{25 }{\left({\text{a}}_{\text{h}}\right)}^{\text{2}}\right]\; \end{array}$$

Using (P_H_), (P_A1_), (P_A2_), and (P_A3_) for alleles necessarily present in the parents (described above) and using (P_h1_), (P_a1_), (P_a2_), and (P_a3_) for alleles not necessarily present, the equation for the probability of concordance in a non-twin sibling of an HLA DRB1*1501-positive proband can be calculated.

Thus, setting (P_a1 _= P_a2 _= P_a3 _= h/r) and (x = x_1 _+ x_2 _+ x_3_) and letting n_1_, n_2 _and n_3 _be the respective number of dominant, recessive and mixed dominant loci among the loci necessarily present in the parents, this expression is:

(46) $$\begin{array}{l} \;\text{P}\left({\text{MS}}_{\text{H}+}\right)\;\;=\;(\sum_{\text{i}=0}^{1}\;[(\text{Pt}\text{*})][\left(\text{1}\right)!/\left(\text{i}\right)!\left(\text{1}-\text{i}\right)!][{\left({\text{P}}_{\text{H}}\right)}^{\text{i}}{\left(\text{1}-{\text{P}}_{\text{H}}\right)}^{\text{1}-\text{i}}])*\\ \;[(\text{1}\;-\sum_{\text{j}=0}^{\text{n}-\text{i}-1}\left[\left({\text{n}}_{\text{1}}\right)!/\left({\text{n}}_{\text{1}}-\text{j}\right)!\left(\text{j}\right)!][{\left({\text{P}}_{\text{A1}}\right)}^{\text{j}}{\left(\text{1}-{\text{P}}_{\text{A1}}\right)}^{\text{n1}-\text{j}}]\right)*\\ \;\;(\sum_{\text{k}=0}^{\text{n}-\text{i}-\text{j}-1}\left[\left({\text{n}}_{\text{2}}\right)!/\left({\text{n}}_{\text{2}}-\text{k}\right)!\left(\text{k}\right)!][{\left({\text{P}}_{\text{A2}}\right)}^{\text{k}}{\left(\text{1}-{\text{P}}_{\text{A2}}\right)}^{\text{n2}-\text{k}}]\right)*\\ \;\;\;(\sum_{\text{m}=0}^{\text{n}-\text{i}-\text{j}-\text{k}-\text{1}}\left[\left({\text{n}}_{\text{3}}\right)!/\left({\text{n}}_{\text{3}}-\text{m}\right)!\left(\text{k}\right)!][{\left({\text{P}}_{\text{A3}}\right)}^{\text{m}}{\left(\text{1}-{\text{P}}_{\text{A3}}\right)}^{\text{n3}-\text{m}}]\right)*\;\\ \;\;\;\;(\sum_{\text{p}=0}^{\;\text{n}-\text{i}-\text{j}-\text{k}-\text{m}-\text{1}}\left[\left(\text{x}\right)!/\left(\text{x}-\text{p}\right)!\;\left(\text{p}\right)!\right]\left[{\left(\text{h}/\text{r}\right)}^{\text{p}}\right]\left[{\left(\text{1}-\text{h}/\text{r}\right)}^{\text{x}-\text{p}}\right])] \end{array}$$

If (n - i -1 > n_1_), (n - i - j -1 > n_2_), (n - i - j - k -1 > n_3_), or (n - i - j - k - m -1 > x), then all further entries of the summations involving (j > n_1_), (k > n_2_), (m > n_3_), or (p > x) are set equal to [0]. NB: In this circumstance (n_1 _+ n_2 _+ n_3 _= n - 1).

For a non-twin sibling of an HLA DRB1*1501-negative proband, this becomes:

(47) $$\begin{array}{l} \;\text{P}\left({\text{MS}}_{\text{H}-}\right)\;=\;(\sum_{\text{i}=0}^{1}\left[(\text{Pt}\text{*})][\left(\text{1}\right)!/\left(\text{i}\right)!\left(\text{1}-\text{i}\right)!][{\left({\text{P}}_{\text{h1}}\right)}^{\text{i}}{\left(\text{1}-{\text{P}}_{\text{h1}}\right)}^{\text{1}-\text{i}}]\right)*\\ \;[(\text{1}\;-\sum_{\text{j}=0}^{\text{n}-\text{i}-\text{1}}\left[\left({\text{n}}_{\text{1}}\right)!/\left({\text{n}}_{\text{1}}-\text{j}\right)!\left(\text{j}\right)!][{\left({\text{P}}_{\text{A1}}\right)}^{\text{j}}{\left(\text{1}-{\text{P}}_{\text{A1}}\right)}^{\text{n1}-\text{j}}]\right)*\\ \;\;(\sum_{\text{k}=0}^{\;\text{n}-\text{i}-\text{j}-\text{1}}\left[\left({\text{n}}_{\text{2}}\right)!/\left({\text{n}}_{\text{2}}-\text{k}\right)!\left(\text{k}\right)!][{\left({\text{P}}_{\text{A2}}\right)}^{\text{k}}{\left(\text{1}-{\text{P}}_{\text{A2}}\right)}^{\text{n2}-\text{k}}]\right)*\\ \;\;\;(\sum_{\text{m}=0}^{\text{n}-\text{i}-\text{j}-\text{k}-\text{1}}\left[\left({\text{n}}_{\text{3}}\right)!/\left({\text{n}}_{\text{3}}-\text{m}\right)!\left(\text{k}\right)!][{\left({\text{P}}_{\text{A3}}\right)}^{\text{m}}{\left(\text{1}-{\text{P}}_{\text{A3}}\right)}^{\text{n3}-\text{m}}]\right)*\\ \;\;\;\;(\sum_{\text{p}=0}^{\;\text{n}-\text{i}-\text{j}-\text{k}-\text{m}-\text{1}}\;\left[\left(\text{x}\right)!/\left(\text{x}-\text{p}\right)!\;\left(\text{p}\right)!\right]\left[{\left(\text{h}/\text{r}\right)}^{\text{p}}\right]\left[{\left(\text{1}-\text{h}/\text{r}\right)}^{\text{x}-\text{p}}\right])] \end{array}$$

If (n - i -1 > n_1_), (n - i - j -1 > n_2_), (n - i - j - k -1 > n_3_), or, (n - i - j - k - m -1 > x), then all further entries of the summations involving (j > n_1_), (k > n_2_), (m > n_3_), or (p > x) are set equal to [0]. NB: In this circumstance (n_1 _+ n_2 _+ n_3 _= n).

As noted earlier, the expected concordance rate would then be the weighted average of these two rates (based on the population prevalence HLA DRB1*1501-positive probands) or:

$$\text{E}\left({\text{CR}}_{\text{S}}\right)\;=\;\left(\text{1}-\text{C}\right)\text{*}\text{P}\left({\text{MS}}_{\text{H}+}\right)\;+\;\left(\text{C}\right)\text{*}\text{P}\left({\text{MS}}_{\text{H}-}\right)$$

### Estimating Concordance in Other Relatives

The observed recurrence risks in parents and children (CR_PC_), in aunts and uncles (CR_AU_), and in first cousins (CR_FC_) place further constraints on the system (5, 6). To predict these risks, however, requires that Equations (46) and (47) to be modified to include modified estimates for (P_A1_, P_A2_, P_A3_, P_H_, and P_h1_). In the case of a dominant allele, for either the biological parent or the child of an MS proband (first degree relatives with 50% genetic sharing), these expressions would be the same as those for a sibling. For the recessive case, however, both parent and child necessarily possess one susceptibility allele, so that their chance of being concordant for MS is:

$${\text{P}}_{\text{A2}}\;=\;\left(0.\text{5}\right)\left({\text{a}}_{\text{2}}\right)\;+\left(0.\text{5}\right)\left({\text{a}}_{\text{2}}\right)\;=\;{\text{a}}_{\text{2}}$$

In the case of an Aunt or Uncle (second degree relatives with 25% genetic sharing), however, the dominant and recessive formulas become:

$$\begin{array}{l} {\text{P}}_{\text{A1}}\;=\left(0.\text{25}\right)\left[\text{1}+{\text{6a}}_{\text{1}}–\text{3}{\left({\text{a}}_{\text{1}}\right)}^{\text{2}}\right]\\ {\text{P}}_{\text{A2}}\;=\;\left(0.0\text{625}\right){\left[\text{1}+{\text{3a}}_{\text{2}}\right]}^{\text{2}}\\ {\text{P}}_{\text{A3}\;}=\;\left[\text{1}–\left({\text{a}}_{\text{3}}\right)/\text{2}\right]\left[{\text{P}}_{\text{A1}}\right]+\left[\left({\text{a}}_{\text{3}}\right)/\text{2}\right]\left[{\text{P}}_{\text{A2}}\right]\\ {\text{P}}_{\text{H}}\;=\;\left(0.\text{25}\right)\left(\text{1}+{\text{6a}}_{\text{h}}–{\text{3a}}_{\text{h}}{}^{\text{2}}\right)\\ {\text{P}}_{\text{h1}}\;=\;\left[\text{1}.\text{5}\left({\text{a}}_{\text{h}}\right)–0.\text{56}{\left({\text{a}}_{\text{h}}\right)}^{\text{2}}\right] \end{array}$$

Whereas for First Cousins (third degree relatives with 12.5% genetic sharing), they become:

$$\begin{array}{l} {\text{P}}_{\text{A1}}\;=\;\left(0.\text{125}\right)\left[\text{1}+{\text{14a}}_{\text{1}}–\text{7}{\left({\text{a}}_{\text{1}}\right)}^{\text{2}}\right]\\ {\text{P}}_{\text{A2}}\;=\;\left(0.0\text{15625}\right){\left[\text{1}+{\text{7a}}_{\text{2}}\right]}^{\text{2}}\\ {\text{P}}_{\text{A3}\;}=\;\left[\text{1}–\left({\text{a}}_{\text{3}}\right)/\text{2}\right]\left[{\text{P}}_{\text{A1}}\right]\;+\left[\left({\text{a}}_{\text{3}}\right)/\text{2}\right]\left[{\text{P}}_{\text{A2}}\right]\\ {\text{P}}_{\text{H}}\;=\;\left(0.\text{125}\right)\left(\text{1}+{\text{14a}}_{\text{h}}–{\text{7a}}_{\text{h}}{}^{\text{2}}\right)\\ {\text{P}}_{\text{h1}}=\;\left[\text{1}.\text{75}\left({\text{a}}_{\text{h}}\right)–0.\text{77 }{\left({\text{a}}_{\text{h}}\right)}^{\text{2}}\right] \end{array}$$

For a child of two parents who each have MS, the probability of either parent having being susceptible at any specific susceptibility locus is (n/x) so that the probability of both parents being in a "susceptible allelic state" at any particular locus is (n/x)^2^. In this case, therefore:

$$\begin{array}{l} {\text{P}}_{\text{A1}}\;=\;\left[\text{1}–{\left(\text{n}/\text{x}\right)}^{\text{2}}\right]\left(0.\text{5}\right)\left[\text{1}+{\text{2a}}_{\text{1}}–{\left({\text{a}}_{\text{1}}\right)}^{\text{2}}\right]\;\\ \;+\;{\left(\text{n}/\text{x}\right)}^{\text{2}}\left[0.\text{75}+\left(0.\text{5}\right){\text{a}}_{\text{1}}–\left(0.\text{25}\right){\left({\text{a}}_{\text{1}}\right)}^{\text{2}}\right]\\ {\text{P}}_{\text{A2}}\;=\;\left[\text{1}–{\left(\text{n}/\text{x}\right)}^{\text{2}}\right]\left(0.\text{25}\right){\left[\text{1}+{\text{a}}_{\text{2}}\right]}^{\text{2}}\;+\;{\left(\text{n}/\text{x}\right)}^{\text{2}}\left(\text{1}\right)\\ {\text{P}}_{\text{A3}}\;=\;\left[\text{1}–\left({\text{a}}_{\text{3}}\right)/\text{2}\right]\left[{\text{P}}_{\text{A1}}\right]\;+\;\left[\left({\text{a}}_{\text{3}}\right)/\text{2}\right]\left[{\text{P}}_{\text{A1}}\right] \end{array}$$

And (if one parent was known to carry the HLA DRB1*1501 allele):

$${\text{P}}_{\text{H}}=\left(0.\text{5}\right)\left[\text{1}+{\text{2a}}_{\text{h}}+{\left({\text{a}}_{\text{h}}\right)}^{\text{2}}\right]$$

And if neither parent carries this allele:

$${\text{P}}_{\text{h1}}=\left[{\text{a}}_{\text{h}}–0.\text{25 }{\left({\text{a}}_{\text{h}}\right)}^{\text{2}}\right]$$

For a recessive allele and both parents affected, the relationship is:

$$\begin{array}{l} {\text{P}}_{\text{A2}}\;=\;{\text{a}}_{\text{2}}+{\left(\text{n}/\text{x}\right)}^{\text{2}}\left(\text{1}–{\text{a}}_{\text{2}}\right)\\ {\text{P}}_{\text{A3}}\;=\text{1}–\left({\text{a}}_{\text{3}}\right)/{\text{2][P}}_{\text{A1}}]\;+\left[\left({\text{a}}_{\text{3}}\right)/\text{2}\right]\left[{\text{P}}_{\text{A2}}\right] \end{array}$$

### Computational Methods

A computer program was written in using the visual basic language on an Excel^® ^(Microsoft Corp.) platform in order to calculate the expected prevalence of MS in the general population and the expected recurrence rates for MS in 1^st^, 2^nd^, and 3^rd ^degree relatives of an MS proband at different parameter values of (**n**) and (**r**). This program substituted the different combinations of (**r **= 0.25, 0.33, 0.5, 1, 2, 4, 8, and 16), (**n **= 5 to 60), and (**x **= 4 to 2000) into Equation (43) in a systematic fashion and then, for each combination of (**r**) and (**n**), determined the values of (**x**) that provided a solution to Equation (43) that fell within the range of:

(C*± 0.05).

The output of this process gave a range of possible values for (**x**) at each combination of (**r**) and (**n**). The high and low ends of this range were taken as the high and low estimates of (**x**) for the specific combination. These values of (**x**) were then used in solving Equation (44) to estimate the prevalence of MS. To calculate the recurrence risks in 1^st^, 2^nd^, and 3^rd ^degree relatives of MS probands, the values of (h, P_h1_, P_H_, P_a1_, P_A1_, p_a2_, P_A2_, P_a3_, and P_A3_) were substituted into Equations (46) and (47) as discussed above, using the values of (**x**) derived from the first step in the process. This program was spot-checked by hand for accuracy of the calculated probability at several different combinations of parameter values for (**n**, **x**, and **r**). In addition it was validated substituting identical probabilities for (h, h/r, P_h1_, P_H_, P_a1_, P_A1_, p_a2_, P_A2_, P_a3_, and P_A3_) and comparing the calculations output by the program to the actual binomial distribution for numerous combinations of (n1, n2, n3, and **x**). This remarked visual basic program is available upon request.

The "Closeness of Fit" (CoF) measure was calculated as the squared percent deviations from published epidemiological (E) data [2,5,6,16] of the calculated the high (H) and low (L) estimates (from the model equations) for the prevalence of MS in the general population and for the concordance rates of MS (from the model equations) in non-twin siblings, parents/children, offspring of conjugal MS couples, and second and third degree relatives (see Table 12). Thus, the equation for this measure was:

$$\text{CoF}=\;{\left[\left(\text{H}-\text{E}\right)/\text{E}+\left(\text{L}-\text{E}\right)/\text{E}\right]}^{\text{2}}$$

These five squared deviations were then summed and the minimum value of the sum determined for the entire matrix extending from (**r **= 0.25 to 16) and from (**n **= 5 to 60). This metric is similar to a chi square calculation for the average deviation of the high and low model predictions from the published epidemiological data for each unique combination of values for (**x**), (**n**), and (**r**). Because each combination was used to generate the entire set of estimates, there was only a single degree of freedom for each set. A chi-square distribution with 1 degree of freedom has a critical value of 3.84. Therefore, any set of estimates with a closeness of fit less the 4 was considered to be reasonably close.

## Results

Tables 4 and 5 show the total number of non-HLA DRB1 genes required (**x**) for different parameter values of (**n**) and (**r**) as well as the estimated disease prevalence at these different combinations. Tables 6, 7, 8, 9, 10, and 11 show the predicted concordance rates for first, second, and third degree relatives of MS probands under the same conditions. These tables are also re-presented for illustrative purposes (with color highlighting for clarity) in the Appendix S1 (see Additional File 1; Section 6). For example, in the case where (**r **= 1), (**n **= 5), and (Pt_1_* = Pt_0_* = 0.134), there must be a total of (**x **+1 = 12-13) susceptibility genes, as indicated in Table 5. Also, from Tables 4, 5, 6, 7, 8, and 9, substituting these values into the appropriate equations yield the estimates of:

$$\begin{array}{lll} \text{E}\left({\text{CR}}_{\text{S}}\right) & = & \text{6}.\text{4}–\text{8}.\text{2}\%\\ \text{E}\left\{\text{P}\left(\text{MS}\right)\right\} & = & \text{1}.\text{8}–\text{2}.\text{4 }\% \end{array}$$

**Table 6:** (5a) Predicted concordance rates of MS in siblings of MS probands assuming (Pt_0 _= Pt_1_) and either 100% Dominant genes or 100% Recessive genes.

	Number of Susceptibility Genes Required (n)						
	**5**	**10**	**11**	**12**	**13**	**14**	**15**

**Frequency of Susceptibility (r)**	**Predicted Concordance in Non-twin Siblings (100% of Genes Dominant)**						

r = 0.25	12.6%	13.2%	13.2%	13.2%	13.1 - 13.4%	13.1 - 13.4%	13.0 - 13.4%

r = 0.33	10.9%	10.7%	9.8 - 11.4%	10.2%	10.7%	10.3 - 11.6%	9.9 - 11.3%

r = 0.5	8.5%	7.9 - 9.1%	7.7 - 8.9%	6.9 - 9.3%	7.5 - 8.6%	8.0 - 9.0%	7.3 - 8.4%

r = 1	7.4 - 8.2%	6.4 - 7.6%	6.3 - 7.4%	6.6 - 7.6%	6.3 - 7.3%	6.5 - 7.5%	6.2 - 7.2%

r = 2	6.6 - 8.0%	6.4 - 7.5%	6.4 - 7.1%	6.2 - 7.2%	6.1 - 7.0%	6.2 - 6.8%	6.0 - 6.9%

r = 4	6.7 - 7.9%	6.3 - 7.3%	6.1 - 7.1%	6.2 - 7.0%	6.1 - 6.9%	6.0 - 6.9%	6.0 - 6.8%

r = 8	6.6 - 7.9%	6.3 - 7.2%	6.2 - 7.1%	6.1 - 7.0%	6.0 - 6.9%	6.0 - 6.8%	6.0 - 6.7%

r = 16	6.6 - 7.9%	6.3 - 7.2%	6.1 - 7.0%	6.1 - 6.9%	6.0 - 6.8%	6.0 - 6.8%	5.9 - 6.7%

**Frequency of Susceptibility (r)**	**Predicted Concordance in Non-twin Siblings (100% of Genes Recessive)**						

r = 0.25	12.6%	13.2%	13.2%	13.1%	13.1- 13.4%	13.1 - 13.4%	13.2 - 13.4%

r = 0.33	10.8%	10.1%	9.6 - 11.3%	9.1 - 10.9%	10.5%	10.1 - 11.5%	9.7 - 11.2%

r = 0.5	8.1%	6.4 - 7.8%	7.0 - 8.2%	6.2 - 8.6%	6.7 - 7.9%	7.2 - 8.3%	6.5 - 7.6%

r = 1	6.4 - 7.2%	5.2 - 6.3%	4.8 - 5.9%	5.0 - 6.0%	4.6 - 5.6%	4.8 - 5.7%	4.5 - 5.4%

r = 2	5.0 - 6.5%	4.1 - 5.1%	4.1 - 4.8%	3.9 - 4.8%	**3.7 - 4.5%**	**3.7 - 4.3%**	**3.5 - 4.3%**

r = 4	4.8 - 6.0%	**3.5 - 4.4%**	**3.3 - 4.2%**	**3.3 - 4.0%**	**3.1 - 3.8%**	**3.0 - 3.7%**	**2.8 - 3.5%**

r = 8	4.3 - 5.7%	**3.1 - 4.0%**	**3.0 - 3.7%**	**2.8 - 3.5%**	**2.6 - 3.3%**	**2.5 - 3.1%**	**2.4 - 3.0%**

r = 16	4.0 - 5.4%	**2.8 - 3.6%**	**2.7 -3.4%**	**2.5 - 3.2%**	**2.3 - 3.0%**	2.2 - 2.8%	2.1 - 2.6%

(Target = 2.9 - 3.8% is in bold; optimal solution underlined).

**Table 7:** (5b) Predicted concordance rates of MS in siblings of MS probands assuming (Pt_0 _= Pt_1_) and either 100% Dominant genes or 100% Recessive genes.

	Number of Susceptibility Genes Required (n)						
	**16**	**17**	**18**	**30**	**40**	**50**	**60**

**Frequency of Susceptibility (r)**	**Predicted Concordance in Non-twin Siblings (100% of Genes Dominant)**						

r = 0.25	13.4%	13.4%	13.3 - 13.4%	13.4%	13.4%	13.4%	13.4%

r = 0.33	9.5 - 11.0%	10.7%	10.4 - 11.6%	9.4 - 11.6%	10.4 - 12.0%	10.2 - 12.3%	10.9 - 13.2%

r = 0.5	7.8 - 8.8%	7.2 - 8.2%	7.7 - 8.6%	6.2 - 8.6%	6.7 - 8.1%	6.4 - 8.3%	6.8 - 11.4%

r = 1	6.0 - 6.9%	6.2 - 7.1%	6.0 - 6.8%	5.5 - 6.8%	5.1 - 6.5%	5.1 - 6.1%	4.8 - 9.6%

r = 2	6.0 - 6.8%	6.0 - 6.6%	5.9 - 6.7%	5.2 - 6.4%	4.9 - 6.0%	4.8 - 5.7%	4.6 - 9.2%

r = 4	5.9 - 6.7%	5.8 - 6.6%	5.8 - 6.5%	5.1 - 6.3%	5.0 - 5.9%	4.8 - 5.6%	4.6 - 9.0%

r = 8	5.9 - 6.7%	5.9 - 6.6%	5.8 - 6.5%	5.1 - 6.2%	4.9 - 5.9%	4.8 - 5.6%	4.6 - 9.0%

r = 16	5.9 - 6.6%	5.8 - 6.6%	5.8 - 6.5%	5.2 - 6.2%	5.0 - 5.9%	4.8 - 5.6%	4.6 - 6.8%

**Frequency of Susceptibility (r)**	**Predicted Concordance in Non-twin Siblings (100% of Genes Recessive)**						

r = 0.25	13.4%	13.4%	13.3 - 13.4%	13.4%	13.4%	13.4%	13.4%

r = 0.33	9.2 - 10.8%	10.5%	10.1 - 11.4%	9.1 - 11.4%	10.5 - 11.8%	9.8 - 12.1%	10.5 - 13.1%

r = 0.5	7.0 - 8.0%	6.3 - 7.4%	6.8 - 7.8%	5.1 - 7.5%	5.2 - 6.7%	4.9 - 6.8%	5.1 - 10.3%

r = 1	4.2 - 5.0%	4.3 - 5.2%	4.1 - 4.9%	**3.2 - 4.3%**	**2.6 - 3.7%**	**2.3 - 3.1%**	**1.9 - 6.2%**

r = 2	**3.3 - 4.1%**	**3.3 - 3.9%**	**3.2 - 3.9%**	2.1 - 2.9%	1.6 - 2.2%	1.3 - 1.7%	**1.0 - 4.0%**

r = 4	**2.7 - 3.3%**	**2.6 - 3.2%**	**2.5 - 3.0%**	1.4 - 2.1%	1.0 - 1.5%	0.7 - 1.1%	0.5 - 2.7%

r = 8	2.3 - 2.8%	2.2 - 2.7%	2.1 - 2.5%	1.1 - 1.6%	0.7 - 1.0%	0.5 - 0.7%	0.3 - 1.9%

r = 16	2.0 - 2.5%	1.9 - 2.3%	1.8 - 2.2%	0.8 - 1.3%	0.5 - 0.8%	0.3 - 0.5%	0.2 - 0.6%

(Target = 2.9 - 3.8% is in bold).

**Table 8:** (6a) Predicted concordance rates of MS in first degree relatives of MS probands assuming (Pt_0 _= Pt_1_), 20% Dominant genes and 80% Recessive genes.

	Number of Susceptibility Genes Required (n)						
	**5**	**10**	**11**	**12**	**13**	**14**	**15**

**Frequency of Susceptibility (r)**	**Predicted Concordance in Non-twin Siblings (Target = 2.9 - 3.8%)**						

r = 0.25	12.6%	13.2%	13.2%	13.1%	13.1 - 13.4%	13.1 - 13.4%	13.0 - 13.4%

r = 0.33	10.8%	10.1%	9.6 - 11.3%	9.1 - 10.9%	10.5%	10.1 - 11.5%	9.7 - 11.2%

r = 0.5	8.1%	6.5 - 7.9%	7.1 - 8.3%	6.3 - 8.7%	6.8 - 8.0%	7.3 - 8.4%	6.6 - 7.8%

r = 1	6.5 - 7.3%	5.4 - 6.6%	5.1 - 6.2%	5.2 - 6.3%	4.9 - 5.9%	5.0 - 6.0%	4.8 - 5.7%

r = 2	5.2 - 6.7%	4.4 - 5.4%	4.5 - 5.2%	4.3 - 5.2%	4.0 - 4.9%	4.0 - 4.6%	3.9 - 4.7%

r = 4	5.0 - 6.2%	3.9 - 4.8%	3.8 - 4.7%	3.7 - 4.5%	**3.5 - 4.2%**	**3.4 - 4.1%**	**3.3 - 4.0%**

r = 8	4.6 - 5.9%	**3.5 - 4.4%**	**3.5 - 4.3%**	**3.3 - 4.0%**	**3.1 - 3.8%**	**2.9 - 3.6%**	**2.9 - 3.5%**

r = 16	4.3 - 5.6%	**3.3 - 4.1%**	**3.2 - 4.0%**	**3.0 - 3.7%**	**2.8 - 3.5%**	**2.6 - 3.3%**	**2.6 - 3.2%**

**Frequency of Susceptibility (r)**	**Predicted Concordance in Parents/Children (Target = 1.8 - 2.1%)**						

r = 0.25	12.6%	13.2%	13.2%	13.1%	13.1 - 13.4%	13.1 - 13.4%	13.0 - 13.4%

r = 0.33	10.7%	9.9%	9.4 - 11.2%	8.9 - 10.8%	10.4%	9.9 - 11.4%	9.5 - 11.0%

r = 0.5	7.7%	5.9 - 7.3%	6.5 - 7.8%	5.7 - 8.2%	6.2 - 7.4%	6.6 - 7.8%	6.0 - 7.1%

r = 1	5.6 - 6.4%	4.3 - 5.4%	3.9 - 5.0%	4.0 - 5.0%	3.6 - 4.5%	3.7 - 4.6%	3.4 - 4.3%

r = 2	3.7 - 5.3%	2.8 - 3.7%	2.9 - 3.6%	2.6 - 3.4%	2.4 - 3.1%	2.3 - 2.8%	2.2 - 2.8%

r = 4	3.3 - 4.5%	**2.1 - 2.8%**	**2.0 - 2.7%**	**1.8 - 2.4%**	**1.6 - 2.1%**	**1.5 - 2.0%**	**1.4 - 1.9%**

r = 8	2.7 - 3.9%	**1.6 - 2.2%**	**1.5 - 2.1%**	**1.3 - 1.8%**	1.1 - 1.6%	1.0 - 1.4%	1.0 - 1.3%

r = 16	2.3 - 3.5%	1.2 - 1.7%	1.2 - 1.7%	1.0 - 1.4%	0.8 - 1.2%	0.7 - 1.0%	0.7 - 0.9%

(Targets are in bold; optimal solution underlined)

**Table 9:** (6b) Predicted concordance rates of MS in first degree relatives of MS probands assuming (Pt_0 _= Pt_1_), 20% Dominant genes and 80% Recessive genes.

	Number of Susceptibility Genes Required (n)						
	**16**	**17**	**18**	**30**	**40**	**50**	**60**

**Frequency of Susceptibility (r)**	**Predicted Concordance in Non-twin Siblings (Target = 2.9 - 3.8%)**						

r = 0.25	13.4%	13.4%	13.3 - 13.4%	13.4%	13.4%	13.4%	13.4%

r = 0.33	9.3 - 10.9%	10.5%	10.2 - 11.4%	9.9 - 11.0%	10.1 - 11.9%	9.9 - 12.1%	10.6 - 13.1%

r = 0.5	7.1 - 8.2%	6.5 - 7.5%	6.9 - 7.9%	5.3 - 7.7%	5.6 - 7.0%	5.2 - 7.1%	5.5 - 10.5%

r = 1	4.5 - 5.4%	4.6 - 5.5%	4.4 - 5.2%	**3.5 - 4.7%**	**3.0 - 4.2%**	**2.8 - 3.6%**	**2.4 - 6.9%**

r = 2	3.8 - 4.5%	**3.8 - 4.3%**	**3.6 - 4.3%**	**2.5 - 3.5%**	2.0 - 2.7%	1.7 - 2.3%	**1.4 - 4.9%**

r = 4	**3.2 - 3.9%**	**3.1 - 3.7%**	**2.9 - 3.6%**	1.9 - 2.7%	1.5 - 2.0%	1.2 - 1.6%	**0.9 - 3.7%**

r = 8	**2.8 - 3.5%**	**2.7 - 3.3%**	**2.6 - 3.1%**	1.5 - 2.2%	1.1 - 1.6%	0.8 - 1.2%	**0.6 - 2.9%**

r = 16	**2.5 - 3.1%**	**2.4 - 2.9%**	2.3 - 2.8%	1.3 - 1.9%	0.9 - 1.3%	0.6 - 0.9%	0.5 - 1.1%

**Frequency of Susceptibility (r)**	**Predicted Concordance in Parents/Children (Target = 1.8 - 2.1%)**						

r = 0.25	13.4%	13.4%	13.3 - 13.4%	13.4%	13.4%	13.4%	13.4%

r = 0.33	9.1 - 10.7%	10.4%	10.0 - 11.3%	8.8 - 11.2%	9.3 - 11.3%	9.6 - 11.9%	10.3 - 13.0%

r = 0.5	6.4 - 7.5%	5.8 - 6.8%	6.2 - 7.2%	4.4 - 6.7%	4.6 - 5.9%	4.1 - 5.9%	4.2 - 9.5%

r = 1	3.2 - 4.0%	3.3 - 4.0%	3.0 - 3.7%	**2.0 - 3.0%**	**1.5 - 2.3%**	1.2 - 1.7%	**0.9 - 4.1%**

r = 2	**2.1 - 2.9%**	**2.0 - 2.4%**	**1.8 - 2.4%**	0.9 - 1.4%	0.6 - 0.9%	0.4 - 0.6%	0.2 - 1.7%

r = 4	**1.4 - 1.8%**	1.2 - 1.6%	1.1 - 1.5%	0.4 - 0.7%	0.2 - 0.4%	0.1 - 0.2%	0.1 - 0.7%

r = 8	0.9 - 1.3%	0.8 - 1.1%	0.7 - 1.0%	0.2 - 0.4%	0.1 - 0.2%	0.0 - 0.1%	0.0 - 0.3%

r = 16	0.7 - 0.9%	0.6 - 0.8%	0.5 - 0.7%	0.1 - 0.2%	0.0 - 0.1%	0.0%	0.0%

(Targets are in bold)

**Table 10:** (7a) Predicted concordance rates of MS in second and third degree relatives of MS probands assuming (Pt_0 _= Pt_1_), 20% Dominant genes and 80% Recessive genes.

	Number of Susceptibility Genes Required (n)						
	**5**	**10**	**11**	**12**	**13**	**14**	**15**

**Frequency of Susceptibility (r)**	**Predicted Concordance in Second Degree Relatives (Target = 0.9 - 1.6%)**						

r = 0.25	12.1%	13.0%	12.9%	12.8%	12.8 - 13.3%	12.7-13.3%	12.6 - 13.3%

r = 0.33	9.2%	7.6%	6.8 - 9.1%	6.1 - 8.4%	7.8%	7.1 - 9.1%	6.5 - 8.5%

r = 0.5	5.6%	3.2 - 4.4%	3.5 - 4.7%	2.8 - 5.0%	3.1 - 4.1%	3.4 - 4.4%	2.7 - 3.7%

r = 1	3.7 - 4.5%	2.1 - 2.9%	1.8 - 2.5%	1.8 - 2.4%	**1.5 - 2.1%**	**1.5 - 2.0%**	**1.3 - 1.8%**

r = 2	2.5 - 3.9%	**1.4 - 2.0%**	**1.4 - 1.8%**	**1.2 - 1.7%**	**1.0 - 1.4%**	**0.9 - 1.2%**	**0.8 - 1.2%**

r = 4	2.4 - 3.5%	**1.1 - 1.7%**	**1.0 - 1.5%**	**0.9 - 1.3%**	**0.8 - 1.1%**	**0.7 - 1.0%**	**0.6 - 0.9%**

r = 8	2.1 - 3.2%	**1.0 - 1.4%**	**0.9 - 1.3%**	**0.7 - 1.1%**	0.6 - 0.9%	0.5 - 0.8%	0.5 - 0.7%

r = 16	1.9 - 3.0%	**0.9 - 1.3%**	**0.8 - 1.1%**	**0.6 - 0.9%**	0.5 - 0.8%	0.5 - 0.7%	0.4 - 0.6%

**Frequency of Susceptibility (r)**	**Predicted Concordance in Third Degree Relatives (Target = 0.9%)**						

r = 0.25	11.8%	12.9%	12.8%	12.7%	12.5 - 13.3%	12.4 - 13.2%	12.3 - 13.2%

r = 0.33	8.3%	6.3%	5.5 - 7.9%	4.8 - 7.1%	6.4%	5.7 - 7.8%	5.0 - 7.1%

r = 0.5	4.4%	2.0 - 3.0%	2.3 - 3.3%	1.7 - 3.4%	1.9 - 2.7%	2.1 - 2.9%	1.6 - 2.2%

r = 1	2.7 - 3.3%	1.1 - 1.7%	**0.9 - 1.4%**	**0.9 - 1.3%**	**0.7 - 1.1%**	**0.7 - 1.0%**	0.5 - 0.8%

r = 2	1.7 - 2.8%	**0.7 - 1.1%**	**0.6 - 0.9%**	0.5 - 0.8%	0.4 - 0.7%	0.4 - 0.5%	0.3 - 0.5%

r = 4	1.6 - 2.5%	**0.5 - 0.9%**	0.5 - 0.7%	0.4 - 0.6%	0.3 - 0.5%	0.3 - 0.4%	0.2 - 0.3%

r = 8	1.4 - 2.4%	0.5 - 0.8%	0.4 - 0.6%	0.3 - 0.5%	0.3 - 0.4%	0.2 - 0.3%	0.2 - 0.3%

r = 16	1.3 - 2.2%	0.4 - 0.7%	0.3 - 0.5%	0.3 - 0.5%	0.2 - 0.4%	0.2 - 0.3%	0.1 - 0.2%

(Targets are in bold; optimal solution is underlined)

**Table 11:** (7b) Predicted concordance rates of MS in second and third degree relatives of MS probands assuming (Pt_0 _= Pt_1_), 20% Dominant genes and 80% Recessive genes.

	Number of Susceptibility Genes Required (n)						
	**16**	**17**	**18**	**30**	**40**	**50**	**60**

**Frequency of Susceptibility (r)**	**Predicted Concordance in Second Degree Relatives (Target = 0.9 - 1.6%)**						

r = 0.25	13.3%	13.2%	13.2 - 13.4%	13.2 - 13.4%	13.3 - 13.4%	13.4%	13.4%

r = 0.33	5.9 - 8.0%	7.4%	6.8 - 8.6%	4.6 - 7.6%	5.0 - 7.7%	4.3 - 7.8%	4.6 - 10.5%

r = 0.5	3.0 - 3.9%	2.4 - 3.3%	2.7 - 3.5%	**1.1 - 2.3%**	**0.9 - 1.4%**	**0.5 - 1.1%**	**0.4 - 3.0%**

r = 1	**1.1 - 1.5%**	**1.1 - 1.5%**	**0.9 - 1.3%**	0.3 - 0.6%	0.1 - 0.3%	0.1%	0.0 - 0.1%

r = 2	**0.7 - 1.0%**	**0.7 - 0.9%**	0.6 - 0.8%	0.1 - 0.3%	0.1%	0.0%	0.0%

r = 4	0.5 - 0.8%	0.5 - 0.7%	0.4 - 0.6%	0.1 - 0.2%	0.0 - 0.1%	0.0%	0.0%

r = 8	0.4 - 0.6%	0.4 - 0.5%	0.3 - 0.5%	0.1%	0.0%	0.0%	0.0%

r = 16	0.4 - 0.5%	0.3 - 0.5%	0.3 - 0.4%	0.0 - 0.1%	0.0%	0.0%	0.0%

**Frequency of Susceptibility (r)**	**Predicted Concordance in Third Degree Relatives (Target = 0.9%)**						

r = 0.25	13.2%	13.1%	13.1 - 13.4%	13.1 - 13.4%	13.2 - 13.4%	13.4%	13.4%

r = 0.33	4.5 - 6.4%	5.8%	5.2 - 7.1%	2.9 - 5.6%	3.1 - 5.4%	2.2 - 5.2%	2.4 - 8.0%

r = 0.5	1.7 - 2.4%	1.3 - 1.9%	1.4 - 2.0%	**0.4 - 1.0%**	0.2 - 0.4%	0.1 - 0.2%	**0.1 - 1.0%**

r = 1	0.4 - 0.7%	0.4 - 0.6%	0.3 - 0.5%	0.1%	0.0%	0.0%	0.0%

r = 2	0.3 - 0.4%	0.2 - 0.3%	0.2 - 0.3%	0.0 - 0.1%	0.0%	0.0%	0.0%

r = 4	0.2 - 0.3%	0.1 - 0.2%	0.1 - 0.2%	0.0%	0.0%	0.0%	0.0%

r = 8	0.1 - 0.2%	0.1 - 0.2%	0.1 - 0.2%	0.0%	0.0%	0.0%	0.0%

r = 16	0.1 - 0.2%	0.1 - 0.2%	0.1%	0.0%	0.0%	0.0%	0.0%

(Targets are in bold)

Neither of these is in line with epidemiological observations. Both the predicted MS concordance in siblings and the predicted disease prevalence from this specific genetic arrangement greatly exceed the observed rates [2,5,6,11,20]. Moreover, as also indicated in the Table 4, regardless of the value assigned to (**r**), when (**n **= 5), the predicted value of P(MS) can never be less than 1.27% (see Additional File 1; Appendix S1; Section 2), which is far too high. Consequently, there must be more than five loci in a susceptible state in order to produce MS susceptibility in an individual.

In fact, perusal of Tables 4, 5, 6, 7, 8, 9, 10, 11, and 12, leads to several other conclusions. First, in the circumstances where (Pt_1 _= Pt_0_) it seems that approximately 18 loci represents an upper bound for the average number of loci needed to be in a susceptible state in order to produce susceptibility to MS. Any more than this and the predicted prevalence will be too low. There are (and will continue to be) solutions at higher values of (**n**), which, as discussed in Additional File 1 (Appendix S1; Section 3), will be increasingly concentrated near the limiting value of (**r **= 0.53). Nevertheless, these solutions are probably spurious as suggested by the Closeness of Fit estimates considering all relatives, which, as shown in Table 13, are best for the solution space in which (11 ≤ **n **≤ 18) and (**r **≥ 2). Moreover, it seems the frequency with which these non-HLA DRB1 loci are in a susceptible state in the general population are less than the frequency of susceptibility at the HLA DRB1 locus because, otherwise, the predicted concordance is too high. The range for the total number of non-HLA DRB1 loci is 35 to 472, although most of the solutions are between 50 and 200 (Tables 4, and 5). Indeed, the optimal solution occurs when 80% of the loci are recessive and at (**x **= 100-107; **n **= 13; and **r **= 4).

**Table 12:** (8) Observed and the optimal predicted concordance rates and prevalence rates for MS under different conditions.

	Observed (Estimated)*	100% Recessive	80% Recessive	100% Dominant	100% Mixed
Number Genes Needed (**n**)	-	14	13	58	58

Frequency of Susceptibility (**r**)	-	2	4	2	2

Total Non-HLA Genes (**x**)	-	58 - 61	100 - 107	246-254	246 - 254

**Relationship**					

Prevalence [2,19]	0.1 - 0.2% (1.5)	0.14 - 0.21%	0.12 - 0.21%	0.00%	0.00%

Non-twin Sibling [5,6]	2.9 - 3.8% (3.0)	3.7 - 4.3%	3.3 - 4.1%.	4.6 - 5.4%	4.5 - 5.3%

Offspring, Conjugal MS**	~10% (10.0)	11.9 - 12.1%	10.3 - 10.8%	13.4%	13.2 - 13.3%

Parent/Child [5]	1.8 - 2.1% (2.0)	1.8 - 2.3%	1.3 - 1.8%	4.6 - 5.4%	4.3 - 5.1%

Second Degree [5]	0.9 - 1.6% (1.0)	0.8 - 1.1%	0.7 - 1.1%	0.1%	0.1%

Third Degree [5]	0.9% (0.9)	0.3 - 0.5%	0.3 - 0.5%	0.00%	0.00%

Closeness of Fit	-	1.8	1.7	22.7	20.6

* The estimates (Targets) used to calculate closeness of fit are shown in parentheses. Closeness of fit was measured as the sum of the squared percent deviations of both the high and the low prediction from the Target. The optimal estimate was taken as the estimate at the values of x, n, and r that gave the closest fit to the observations.

** The concordance rate for the offspring of Conjugal MS is based on the report of Sadovnick et al. [6], in which the recurrence rate in offspring of two parents with MS is reported to be 78% of the monozygotic twin rate (CR_MZ_).

**Table 13:** (9) "Closeness of Fit" Calculations.

	Number of Susceptibility Genes Required (n)						
	
	5	10	11	12	13	14	15
**Frequency of Susceptibility (r)**	**Closeness of Fit (Target ≤4.0)**						

r = 0.25	24,106	29,422	28,838	28,228	29,694	29,258	28,805

r = 0.33	19,148	4,872	5,600	4,166	4,788	5,416	4,150

r = 0.5	2,139	459	555	451	320	378	189

r = 1	789	90	43.9	39.7	18.9	17.7	9.4

r = 2	394	21.4	12.1	6.9	**3.3**	**2.4**	**2.5**

r = 4	337	9.9	4.4	**2.2**	**1.7**	**2.1**	**3.0**

r = 8	286	6.8	**3.1**	**1.9**	**2.3**	**3.4**	4.4

r = 16	262	5.8	**2.8**	**2.4**	**3.3**	4.6	5.8

	**Number of Susceptibility Genes Required (n)**						

	**16**	**17**	**18**	**30**	**40**	**50**	**60**

**Frequency of Susceptibility (r)**	**Closeness of Fit (Target ≤ 4.0)**						

r = 0.25	31,198	30,935	31,545	31,749	32,041	32,713	32,776

r = 0.33	3,137	3,593	4,106	1,459	1,280	885	2,042

r = 0.5	228	114	140	21.4	18.3	18.7	38.0

r = 1	5.9	6.2	4.7	8.5	10.6	11.8	11.6

r = 2	**3.0**	**3.6**	4.2	10.9	13.5	14.9	12.7

r = 4	**4.0**	5.1	6.3	13.2	15.5	16.8	14.9

r = 8	5.5	6.8	8.0	14.7	16.7	17.7	16.2

r = 16	6.8	8.1	9.4	15.6	17.3	18.2	18.3

* (Targets are in bold; optimal fit is underlined)

* Calculated as the sum of the squared percent deviations from published epidemiological observations (E) of the high (H) and low (L) estimates derived from the model for non-twin siblings, parents/children, offspring of conjugal MS couples, and second and third degree relatives of MS probands. For each category, this squared percent deviation is defined as: [(H - E)/E + (L - E)/E]^2^

Naturally, the situation changes when the penetrance ratio (Pt_1_/Pt_0_) is altered. In general, as (Pt_1_) increases (and as C* approaches its limit of 0.79), the number of necessary loci (**n**) increases. For example, at the value of (Pt_1 _= 0.27), the point of optimal fit occurs at (**n **= 19) and (**r **= 16) with a Closeness of Fit estimate of (3.36). If all the alleles are recessive, the optimal fit occurs at (**n **= 22) and (**r **= 4) with a Closeness of Fit of (3.25). In this last circumstance, the solution space is the solution space in which (18 ≤ **n **≤ 26) and (**r **≥ 4). After the limit of (C* = 0.79) is reached, for any increase in this ratio, the predicted concordance increases (rather than decreases) so that it can never approach the observed values for concordance and prevalence (see Additional File 1; Appendix S1; Section 3). Similarly, the number of necessary loci (**n**) also increases either as (h) is increased above the observed value of (h = 0.24) or as (h_m_) is decreased below the observed value of (h_m _= 0.55). By contrast, (**n**) decreases as either (h) is decreased or (h_m_) is increased. The Closeness of Fit estimates under these conditions, however, is generally worse.

As shown in Table 14, when the odds ratio for the HLA DRB1*1501 allele is adjusted to equal its observed value of (OR = 3.3) for conditions (h = 0.24; h_m _= 0.55), the expected odds ratios for recessive non-HLA DRB1 loci (when Pt_0 _= Pt_1_) is approximately (OR ≈ 1.6 - 1.7). By contrast, the expected odds ratio for dominant non-HLA DRB1 loci is (OR ≈ 2.2) under these same conditions (Table 15). These odds ratios are altered both as (Pt_1_), (h), or (h_m_) are changed from their observed values and also when more than one susceptibility allele is assumed to be present at a locus (see Additional File 1; Appendix S1). For example, at the value of (Pt_1 _= 0.27) and with 2 susceptibility alleles at each non-HLA DRB1 locus but with (h) and (h_m_) unchanged, the expected odds ratio for recessive alleles at loci, in which heterozygotes having two different recessive alleles still confers susceptibility, is (OR = 1.3 - 1.4), whereas, for dominant alleles, it is (OR = 1.7 - 1.9).

**Table 14:** (10) The Estimated Prevalence and the Number of Loci (**n**) for susceptible genotypes that include the HLA DRB1*1501 allele.

	Estimated Number of Susceptibility Genes Required (n) for all Loci						
	**11**	**12**	**13**	**14**	**15**	**16**	**17**

**Frequency of Susceptibility (r)**	Estimated Prevalence of HLA DRB1*1501 in an MS Population (unadjusted; OR ≈ 2.21)						

r = 0.25	0.25	0.26	0.24 - 0.26	0.24 - 0.26	0.24 - 0.26	0.25	0.25

r = 0.33	0.32 - 0.37	0.33 - 0.39	0.35	0.32 - 0.36	0.33 - 0.37	0.34 - 0.38	0.34

r = 0.5	0.39 - 0.42	0.38 - 0.44	0.39 - 0.42	0.39 - 0.41	0.39 - 0.42	0.38 - 0.41	0.40 - 0.42

r = 1	0.42 - 0.44	0.41 - 0.43	0.41 - 0.44	0.41 - 0.43	0.41 - 0.43	0.42 - 0.44	0.41 - 0.43

r = 2	0.42 - 0.44	0.41 - 0.44	**0.41 - 0.44**	**0.42 - 0.43**	**0.41 - 0.43**	**0.42 - 0.43**	**0.42 - 0.43**

r = 4	0.42 - 0.44	**0.42 - 0.44**	**0.42 - 0.44**	**0.41 - 0.43**	**0.42 - 0.43**	**0.42 - 0.43**	0.42 - 0.43

r = 8	**0.42 - 0.44**	**0.42 - 0.44**	**0.41 - 0.43**	**0.42 - 0.43**	0.42 - 0.43	0.41 - 0.43	0.42 - 0.43

r = 16	**0.42 - 0.44**	**0.42 - 0.44**	**0.42 - 0.43**	0.42 - 0.43	0.42 - 0.43	0.42 - 0.43	0.41 - 0.43

**Frequency of Susceptibility (r)**	**Odd Ratios for Recessive non-HLA DRB1 Loci (after adjustment of HLA to OR = 3.34)**						

r = 0.25	1.21	1.23	1.05 - 1.23	1.05 - 1.27	1.07 - 1.29	1.07	1.08

r = 0.33	1.57 - 2.03	1.66 - 2.15	1.66 - 2.15	1.51 - 1.82	1.57 - 1.91	1.64 - 2.00	1.70

r = 0.5	1.88 - 2.15	1.79 - 2.30	1.79 - 2.30	1.82 - 2.01	1.91 - 2.12	1.84 - 2.01	1.92 - 2.11

r = 1	1.86 - 2.05	1.82 - 1.98	1.82 - 1.98	1.82 - 1.96	1.85 - 1.98	1.88 - 2.01	1.84 - 1.96

r = 2	1.75 - 1.85	1.73 - 1.85	**1.73 - 1.85**	**1.75 - 1.83**	**1.74 - 1.84**	**1.74 - 1.84**	**1.75 - 1.82**

r = 4	1.65 - 1.78	**1.66 - 1.75**	**1.66 - 1.75**	**1.65 - 1.74**	**1.66 - 1.74**	**1.66 - 1.74**	1.66 - 1.73

r = 8	**1.60 - 1.69**	**1.60 - 1.69**	**1.60 - 1.69**	**1.60 - 1.67**	1.60 - 1.67	1.60 - 1.67	1.61 - 1.66

r = 16	**1.56 - 1.64**	**1.56 - 1.64**	**1.56 - 1.64**	1.56 - 1.63	1.56 - 1.62	1.57 - 1.62	1.56 - 1.62

(Bold and underlined as designated for "Closeness of Fit" calculations in Table 8)

**Table 15:** (11) The estimated Number of Loci (**n**) in for Genotypes including or not including HLA DRB1*1501.

	Estimated Number of Susceptibility Genes Required (n) for all Loci						
	**11**	**12**	**13**	**14**	**15**	**16**	**17**

**Frequency of Susceptibility (r)**	Estimated (n) the for Genotypes including HLA DRB1*1501						

r = 0.25	3	3	3	3	3	3	3

r = 0.33	3 - 10	3 - 11	11 - 11	3 - 13	11 - 14	14 - 15	15

r = 0.5	10 - 11	11 - 12	12 - 13	13 - 14	14 - 15	15 - 16	17

r = 1	11	12	13	14	15	16	17

r = 2	11	12	**13**	**14**	**15**	**16**	**17**

r = 4	11	**12**	**13**	**14**	**15**	**16**	17

r = 8	**11**	**12**	**13**	**14**	15	16	17

r = 16	**11**	**12**	**13**	14	15	16	17

**Frequency of Susceptibility (r)**	Estimated (n) the for Genotypes not including HLA DRB1*1501						

r = 0.25	12	13	14 - 15	15 - 16	16 - 17	18	19

r = 0.33	11 - 12	12 - 13	14	15	15 - 16	16 - 17	18

r = 0.5	11	12	13	14	15	16	17

r = 1	11	12	13	14	15	16	17

14 - 15r = 2	11	12	**13**	**14**	**15**	**16**	17

r = 4	11	**12**	**13**	**14**	**15**	**16**	17

r = 8	**11**	**12**	**13**	**14**	15	16	17

r = 16	**11**	**12**	**13**	14	15	16	17

(Bold and underlined as designated for "Closeness of Fit" calculations in Table 8)

Second, as shown in Table 5 and 6, there are no appropriate solutions when all of the loci are assumed to be "dominant". Thus, the smallest recurrence rate in the case of 100% "dominant" loci (and with Pt_0 _= Pt_1_) is greater than 4.6% and the Closeness of Fit estimate is poor (Table 13). If (Pt_0 _< Pt_1_), this calculated recurrence rate only increases. The same is true for mixed dominance loci (Table 13). In fact, only when a small fraction of the loci are assumed to be "dominant" does the closeness of fit become good. Similarly, mixed dominance loci also provide poor solutions (Table 13), presumably because they effectively function as "dominant" loci at low allelic frequencies. Moreover, it is of note that all of these calculations have been made on the "adjusted" estimates of penetrance (Pt*; see Table 3) based on the observation that dizygotic twins (with their shared intrauterine environment) seem to have a greater penetrance of MS compared to non-twin siblings [11]. If the same calculations were made using an unadjusted estimate of penetrance (Pt), these calculated recurrence rates would be almost twice as high and the Closeness of Fit estimate substantially worse compared to the use of adjusted penetrance estimates.

It seems that the number of loci necessary to be in a susceptible state in order to confer susceptibility to MS is smaller for patients who carry the HLA DRB1*1501 allele compared to susceptible individuals who don't carry this allele (Additional File 1; Appendix S1; Section 4). Using Equations (79) and (80) to estimate this difference, Table 15 shows the estimated number of loci at different combinations of (**n**), (**x**), and (**r**). Notably, within the solution space of (11 ≤ **n **≤ 18) and (**r **≥ 2), even though there is a small difference in the average number of loci required between the two sub-groups, the estimate for (**n**) is exactly the same for both, indicating that this difference is quite small (i.e., less than 1 locus). Even if one makes the assumption that only individuals who are homozygous for the HLA DRB1*1501 allele require fewer loci to be in a susceptible state, the difference in the estimated value of (**n**) between subgroups within this solution space is still only about 1 locus.

## Discussion and conclusions

This paper explores the genetic basis of MS pathogenesis through the lens of a mathematical model of genetic susceptibility and a critical analysis of the currently available epidemiological information about this illness. This is not to downplay the importance of environmental factors in disease pathogenesis. Indeed, these factors were the principal focus of previous work [10]. Rather, the focus of this paper is on the attempt to understand, not the genes that lead to MS susceptibility but, rather, the basis and importance of genetic susceptibility to this illness.

Several results seem particularly noteworthy. In earlier publications, the critical environmental factors have been suggested to be "population wide " exposures [10,22-27]. Intriguingly, a similar conclusion can be reached by a mathematical analysis, in which that these environmental exposures (whatever they are) can be shown to be extremely common events [10]. By contrast, the genetics of MS seems to be of critical importance with regard to disease pathogenesis. Thus, the analysis presented in this manuscript demonstrates that the large majority of individuals who develop MS (possibly all) must have, in part, a genetic basis for their disease. Moreover, to underscore the importance of genetic susceptibility to disease pathogenesis, the mathematical analysis of the present manuscript demonstrates that, under any circumstance, only a tiny fraction of the general population (<2.2%) is genetically susceptible to getting this illness. Finally, the derived model demonstrates that the possibilities for the number of susceptibility loci (and the number of involved loci necessary to confer that susceptibility) are quite limited (Tables 4, 5, 6, 7, 8, 9, 10, and 11). Indeed, it seems that genetic susceptibility is, by far, the most important factor in disease pathogenesis. Thus, whereas environmental factors (while necessary) are very common population-wide events [10,22-27], only a very small fraction of the general population are genetically capable of getting the disease, regardless of what occurs to them during life. This conclusion is not altered at all by the recent report of Baranzini and co-workers [31], which reported that there were no genetic or epigenetic differences between elderly monozygotic twins who were clearly discordant for MS. This finding is anticipated. Even if everyone who developed MS had to be genetically susceptible (and as both MZ twins will be if one has MS), the expected concordance rate in MZ twins is still only 25% in the northern North America and northern Europe. This study only serves to underscores the importance of an environmental contribution to MS pathogenesis - a conclusion that was clearly evident decades prior to this publication [2-11,22-27].

A previous paper also explored a mathematical model of MS Genetics in order to determine both the number of risk alleles required and their allelic frequency [32]. In this paper, the author approached the problem by using observed value for the lifetime risk of MS [P(MS)] and the monozygotic-twin concordance risk (CR_MZ_), together with different numbers of risk alleles with different frequencies, to predict the recurrence risk in both first and second degree relatives of an MS proband. Conceptually, this author's view of MS susceptibility is that MS risk increases either with the number of risk alleles in an additive or a multiplicative manner or as a step function where (in the case of 10 total alleles) the risk for 6 or fewer alleles was 0 and the risk for 7 or more alleles was 0.25 (i.e., the CR_MZ_). His conclusion was that the best fit with existing data was for autosomal dominant models having either a strong interaction between the different loci, in which risk increases rapidly with each additional disease allele or, better yet, a step function [32]. When 10 loci are present, the allelic frequency of the susceptibility alleles was calculated to be (0.15 - 0.31). The author then explored the impact of changing the number of presumed risk alleles (in models using a step function where the step occurred at 100%, 67% and 33% of the number of alleles). In each case, the allelic frequency was adjusted so that the predicted population probability of MS [P(MS)] was always 0.2%. Using this strategy, the author reported that four different models fit the observed recurrence risk data. Thus, dominant models in which 100% of 6 risk alleles were required for susceptibility, dominant models with 9 or more risk alleles and 67% required, dominant models with 15 or more risk alleles and 33% required, and a recessive model in which 100% of 2 or 3 risk alleles were required, all fit the data. Moreover, using this model, the author found no upper limit to the number of alleles possible although, with an increasing number, the allelic frequencies increased toward 1.0 [32].

In many ways this earlier model [32] is a subset of the model proposed here. Thus, following the argument in Section 1, this model would still require there to be a total of (**n**) susceptibility loci to be in a susceptible state in order for an individual to be genetically susceptible. Moreover, the notion of a step function (going suddenly from non-susceptibility to susceptibility once a certain number of risk alleles are present) is common to both schemes. However, in the previous model, this step function was inferred from the "closeness of fit" analysis of the predicted recurrence rates whereas, in the present paper, it is used as a convenience to describe a much more complicated and interactive underlying susceptibility structure (see Section 1). In addition, there is no provision in the previous model for a possible difference in penetrance of different combinations of risk alleles. Neither of these differences, however, is critical and, in fact, they are both likely to be of no importance whatsoever.

Rather, the critical difference between the models is that the previous model is unbounded precisely because it is has not been tied securely to the epidemiological realities of MS and because of its conceptualization of susceptibility seems biologically unlikely. For example, the author concludes that one possibility is a recessive model in which 2 or 3 risk alleles are present and all are required. Clearly, however, such a model is untenable. First, the HLA DRB1*1501 allele is an established risk allele for MS and it is known to be "dominant" in sense that both heterozygous and homozygous states confer susceptibility [12-15]. Consequently, not all risk alleles can be recessive. Second, if a 100% of anything was required, then every patient with MS would be in a susceptible allelic state at the HLA DRB1 locus, a circumstance which is claimed by no one. Thus, the previous model has failed to incorporate the known epidemiological information about the only allele (HLA DRB1*1501), which has been securely linked to MS susceptibility. Third, and most importantly, the cutoffs of 100%, 67% and 33% for the step function are arbitrary. In and of itself, the use of arbitrary cut-points makes little difference and, as the author states (somewhat confusingly), other cut-points were also explored. Rather, it is the use of such cut-points, in the first place, that indicates a fundamental conceptual difference in the nature of MS susceptibility between the two approaches. Thus, in the previous model [32], MS susceptibility is held, not to be the result of an individual possessing a specific combination of susceptibility alleles, but rather, to be the result of possessing a certain fraction of the total number such alleles. Naturally, in such a circumstance, because the total number of possible risk alleles is unbounded (other than by the entire genome), so too is the number of alleles required for MS susceptibility. Naturally, also, the allelic frequency of these putative susceptibility alleles increases as their number increases in order to maintain the population prevalence at 0.2%.

By contrast, in the present paper, the genetic susceptibility to developing MS is conceptualized as occurring when, from amongst a total of (**x **+ 1) susceptibility loci (haplotypes) spread throughout the genome, an individual possesses a specific combination of some of these loci, each of which is in a specific susceptible state. Although, at each locus, there may be more than one "susceptibility" gene and, for any gene, there may be more than one "susceptibility" allele, the net effect of the interaction of these different genes and different alleles at a particular locus is presumed to put this locus into a "susceptible" state or not. Moreover, different specific combinations of different numbers of these susceptibility loci each having specific "susceptibility genotypes" are envisioned to produce susceptibility and the entire set of such "susceptible" genetic combinations is taken to define the subset of individuals in the general population who could potentially get MS in the right environmental circumstances. If an individual does not possess one of these susceptible genetic combinations, then they cannot get MS regardless of what environmental events they experience in their lives. Alternatively, of course, it is possible that only some (but not all) of MS is genetic (in the sense described above) and that some individuals may get this illness through a purely environmental mechanism. Nevertheless, the evidence (such as it exists) suggests that the vast majority (and likely all) cases of MS are the result of a genetically susceptible individual experiencing a sufficient (but complex) environmental exposure, which includes multiple different events occurring at different times during their life [10]. The available evidence also suggests strongly that genetic susceptibility to MS is a rare occurrence. Thus, as noted above, only 2.2% or less of the general population is susceptible to getting MS. Even among individuals who carry the HLA DRB1*1501 allele, the probability of being susceptible to getting MS is still only ~2.6% (Additional File 1; Appendix S1; Section 4).

As discussed in the Introduction, this conceptualization reflects a binary view of genetic susceptibility. This is not, however, a fundamental assumption of the model. Rather, the binary nature of model is a consequence of the concept of susceptibility. For example, if everyone is genetically susceptible, then the influence of genetic factors is to alter the penetrance of the different genotypes. By contrast, if some individual are not susceptible while others are, then susceptibility is binary, not because of the model but because of the nature of susceptibility. Importantly, in the model, even though susceptibility was conceived as binary, this was not forced into the final result. Thus the term P(G) was unconstrained and could have been 100%. The limit of [P(G) ≤ 2.2%] was set by the constraint of epidemiological observations - not by a constraint inherent to the model.

Placed into this context, the model derived in this manuscript provides considerable insight into the nature of the genetic basis for MS. Indeed, the current epidemiological observations of (h = 0.24; h_m _= 0.55; P(MS) = 0.0015; and Pt_0 _= Pt_1 _= 0.25), suggest that the upper limit for the average number of susceptibility loci (**n**) that need to be in a susceptible state for an individual to be susceptible to getting MS is (11 ≤ **n **≤ 18). Moreover, the total number of non-HLA DRB1 loci (**x**) that contribute to susceptibility seems to be between 50 and 200, and that the frequency of susceptibility at these loci is approximately (h/r ≤ 0.12) or (**r **≥ 2). In fact, the genetic configuration that best fits these epidemiological observations, the current prevalence estimates, and the concordance data for non-twin siblings, parents and children, children of conjugal MS couples, second degree relatives, and third degree relatives occurs when 80% of the loci are recessive and at (**x **= 100-107; **n **= 13; and **r **= 4). Indeed, the prevalence and recurrence risks predicted by these particular values match the actual epidemiological observations quite closely (Table 13). It is of note that these predicted recurrence risks have been calculated using a penetrance estimate that has been down-weighted from the identical-twin concordance rate because of the apparently important influence of the shared intra-uterine or early post-natal environment [10]. If an unadjusted penetrance had been used, all of the estimated recurrence rates would have been approximately double and none of models (recessive, dominant, or mixed) would have provided a good fit with the actual epidemiological data. Such a finding, independently, tends to validate the importance of the intra-uterine and/or early post-natal environment in MS pathogenesis.

It also seems likely that either the large majority of the (**x**) susceptibility loci must be "recessive" (in the sense described in the Additional File 1; Appendix S1) or there must be more than one susceptibility gene present at each susceptibility locus and that these genes must combine in such a way that only a small fraction of the possible combinations produce a susceptible state at the locus (Additional File 1; Appendix S1). There are three reasons for this conclusion. First, and most important, the predicted recurrence risk for MS in siblings for a single dominant gene (even one with multiple different susceptibility alleles) seems too high to explain the epidemiological observations (Tables 4, 5 and 11). Second, the optimal fit for the predicted with the observed data occurs when only 20% of the loci are assumed to be "dominant" (Table 8). Third, the observed odds ratios (OR = 1.1 - 1.3) for different candidate genes at non-HLA DRB1 loci in genome-wide association studies [12-15] seems too small to be easily explained by the alterations of the parameters of (h), (h_m_), and (Pt_1_) for "dominant" alleles. In addition, altering these parameters generally results in a Closeness of Fit estimates, which are both too high and worse compared to the estimate using the observed parameter values of (h = 0.24), (h_m _= 0.55), and (Pt_1 _= 0.25). This last piece of evidence, however, may not make a compelling argument because the odds ratio can also be markedly affected by the use of single SNPs to identify alleles. Thus, depending upon the exact nature of the relationship between the state of the DNA at the SNP location and the polymorphic alleles of any particular susceptibility gene, the observed odds ratio (even for dominant alleles) can be dramatically reduced (see Additional File 1; Appendix S1; Section 5).

One difficulty with the use of genome-wide association screens to identify susceptibility loci is that, due to multiple statistical comparisons and random sampling error, all such screens will be quite susceptible to both the false positive and the false negative identification of loci. If the bar for association is set too low, false positives will greatly outnumber false negative identifications. By contrast, if the bar is set too high, false negatives will greatly outnumber false positive identifications. Compounding the difficulties of sorting out false positive and false negative identifications, is the fact that the distinction between a true susceptibility locus and a disease-modifying locus will be problematic. Thus, although only (**x **+ 1) susceptibility loci are present in the entire genome, there may be many other loci that can modify the clinical expression of MS by either by changing the actual penetrance of MS in susceptible individuals or by changing the apparent penetrance, for example, by altering the disease severity or the phenotype of the illness.

Regardless of the mechanism however, on a genome-wide association screen, any locus that has such an effect on penetrance (real or otherwise) will appear to be positively or negatively associated with the illness. For example, if the presence of a particular allele of a particular gene (not involved in MS susceptibility) doubled the penetrance of MS for all susceptible combinations, the odds ratio for an association of this allele with MS would be (2.0) and highly significant, despite the fact that this allele would not be a "susceptibility" allele and the locus that harbored this allele would not be a "susceptibility" locus in the sense defined earlier (i.e., this would be a false association). Moreover, because the model places no constraints on the possible number of these disease-modifying loci, many of the observed associations (even highly significant and/or well replicated ones) may have a substantial probability of representing a false association with the genetic susceptibility to MS. Consequently, because susceptibility loci and disease modifying loci will be identified equally well by genome-wide screens, unraveling the two will not be possible using this approach. One possible method for establishing that an MS-associated allele was a true susceptibility allele (e.g., the HLA DRB1*1501 allele) would be to demonstrate that it doesn't alter the penetrance sufficiently to account for the observed odds ratio on genome-wide screens (e.g., Table 3). For most associations, however, such a method will be difficult both because the available identical-twin data to assess penetrance differences is limited and because the observed odds ratios for candidate genes are typically small [15].

Considering the results of several genome-wide association screens [2,12-15,33,34], it has been relatively easy to identify the HLA DRB1 locus (haplotype) in general, and the 1501 allele in particular, as associated with MS. In addition, the observed odds ratio for an association of this chromosomal region with MS has been much larger (and much more consistent) compared to other potential candidate loci [12-15]. Indeed, the strength and uniqueness of this association has led many investigators to conclude that genetic variation within this chromosomal region is principally responsible for genetic susceptibility to MS [2,12-15]. Consideration of the model proposed here and some of the observations made from it, however, might be taken to raise questions about such a conclusion. First, the HLA DRB1 locus (haplotype) seems to be only one among a hundred or more loci that are involved in MS susceptibility. Second, although the frequency of having at least one copy of the HLA DRB1*1501 allele in the general population is approximately four times the frequency of susceptibility at non-HLA DRB1 loci, the penetrance of susceptible genotypes that include this allele is no different from those that don't (Table 3). Third, although the number of other susceptibility loci that need to be involved is smaller when this allele is present, the actual difference is less than 1 locus (Table 15). In circumstances where a genetically susceptible genotype requires involvement of 11-18 total loci (Table 4), this difference seems negligible. Fourth, almost a half of the genetically susceptible individuals, lack this allele entirely. Moreover, only a small fraction of those individuals who carry this allele (≤ 5.2%) are even susceptible to getting this MS in the first place (Additional File 1; Appendix S1; Section 4). In this context, the apparent predominance of the HLA DRB1*1501 allele in MS pathogenesis seems likely related to three factors (see Additional File 1; Appendix S1; Section 5). First, this allele is one of the uncommon dominant susceptibility alleles and these have greater associated odds ratios than recessive alleles. Second, susceptible genotypes including this allele have a slightly smaller number of involved loci compared to those genotypes without it, a circumstance that will inflate the observed odds ratio for the HLA locus but not for the non-HLA loci. And, third, the use of SNPs to represent the allelic structure of the genome will markedly reduce the observed odds ratio for many (possibly most) true susceptibility non-HLA loci regardless of whether they are dominant or recessive.

This might also help to explain the observation that some of the identified SNPs have relative allelic frequencies (RAFs) for some of the identified susceptibility loci, which are unexpectedly high [13]. For example the interleukin 7 receptor (ILR7) gene using SNP (rs6897932) has an RAF of (0.75), whereas the IL2 receptor alpha (IL2RA) gene using SNPs (rs12722489 and rs2104286) has RAFs of (0.85) and (0.75) respectively. Several phenomenon may account for this apparent paradox. First, if any of the these SNPs tagged more than one allele (see Additional File 1; Appendix S1; Section 5), this would increase the "apparent" allelic frequency of the true susceptibility allele. Indeed, the occurrence of different RAFs for the same locus (as is seen above for IL2RA gene) presumably indicates that the allelic structure is not simple even though, in this case, the difference is quite small. Second, even if the SNP is located in the coding region of a particular gene and is known to cause a functional change in the coded protein by introducing a stop codon or a non-synonymous amino acid substitution, altering splice sites, or changing the binding characteristics of regulatory molecules (e.g. 33, 34), this does not prove either that this functional change is what caused susceptibility or that this gene is involved with susceptibility. Even in this circumstance, the association only identifies the region of the genome wherein susceptibility resides. Third, even in the circumstance where the SNP association has identified the correct gene and causes a functional change in the coded protein, this still falls short of proving causation. It could be that a second alteration in this gene, together with the identified SNP, identifies the true susceptibility allele (see Additional File 1; Appendix S1; Section 5). And fourth, the method of genome-wide association screening is set-up to identify associations with SNPs of high frequency (i.e., major alleles) and to ignore minor alleles.

Indeed, the fact that the genes identified to date (with the exception of the HLA DRB1 locus produce such low ORs [2,12-15,33,34], especially in the circumstance where the genetics of MS is, by far, the most important contributor to disease incidence, makes it seem likely that some, perhaps all, of these physiologic mechanisms are occurring. Clearly, we have a long way to go to understand the specifics of MS susceptibility. Nevertheless, gaining some insight to the number of susceptibility loci involved, the number of loci needed to be in a susceptible state, and the average frequency of susceptibility at these susceptibility loci represents progress.

## Competing interests

The author declares that he has no competing interests.

## Authors' contributions

DSG carried out the mathematical analysis, wrote the Excel program, and wrote the paper. The sole author read and approved the final manuscript.

## Pre-publication history

The pre-publication history for this paper can be accessed here:

http://www.biomedcentral.com/1471-2377/10/101/prepub

## Supplementary Material

Additional file 1 **Appendix S1**. Mathematical derivations used for the development of the model. Click here for file
