# Supplementary material for: The genetic basis of multiple sclerosis: a model for MS susceptibility
Source: BMC Neurol. 2010 Oct 28;10:101. doi: 10.1186/1471-2377-10-101 (PMC2994805; doi:10.1186/1471-2377-10-101)
Supplement: Additional file 1 — Appendix S1. Mathematical derivations used for the development of the model. [file 1471-2377-10-101-S1.PDF]

Appendix S1

1. Use of Average Allelic Frequencies and a Specific Number of Loci……………….2

2. Relationships between **x**, **r**, and **n**…………………………………………..….4

3. Prevalence and Concordance……………………………………………………...7

4. Susceptibility Stratified by HLA DRB1*1501 Status……………………………….12

5. Odds Ratios for Polymorphic Alleles or Multiple Genes at Susceptibility Loci........20

6. Tables……………………………………………………………………………..26

1. Defining the Nature of the Susceptibility Loci

Several possible "susceptible allelic states" are envisioned in the Model. First, as is the case for the HLA DRB1 locus, a "true-susceptibility allele" (or alleles) may be present as a "dominant" trait. This state will be defined as one in which a single copy of the allele (or these alleles), when present, increases the likelihood of the individual being susceptible to getting MS (i.e., these loci confer susceptibility in a dominant fashion). Second, it is possible that one or more true-susceptibility alleles may be present as a "recessive" trait. This state is defined as one in which two susceptibility alleles are necessary to confer susceptibility. Third, it is possible that some "dominant" alleles at certain genetic loci confer protection (rather than susceptibility) on an individual. In the Model, therefore, it will be the absence of this allele (or these alleles) at the specific genetic locus that will be considered to confer susceptibility. This susceptible allelic state is mathematically equivalent to the "recessive" state for a true-susceptibility allele because a susceptible individual will need to have both alleles at these loci be non-protective variants in order to confer susceptibility. Fourth, it is possible for protective alleles to be present as a "recessive" trait. This state is defined as one in which two copies of the allele are necessary to confer protection, and this state is mathematically equivalent to that for a "dominant" true-susceptibility allele. Fifth, it is possible that both recessive and dominant true-susceptibility alleles co-exist at the same locus, in which case there would be a mixture of dominance and the "frequency of susceptibility" would reflect a combination of these two allelic states.

Sixth, it is possible that a single genetic location has both dominant protective and dominant true-susceptibility alleles. If the protection only affects susceptibility arising from other alleles at the same genetic location, then only the penetrance of the true-susceptibility allele (or alleles) will be altered. Thus, if both alleles are present in the same genome, the true-susceptibility allele will be less likely confer susceptibility compared to when the true-susceptibility allele is present by itself. Nevertheless, the presence of this allele should still be associated with an increased likelihood of an individual being susceptible to getting the disease. For example, even if the protective allele completely abrogated the effect of the true-susceptibility allele, and if the protective/true-susceptibility heterozygote accounted for 10% of true-susceptibility-positive individuals, this circumstance would only reduce the apparent

penetrance of the true-susceptibility haplotype by 10%. By contrast, if the protection extended to susceptibility conferred by other genetic loci, the situation is somewhat more complicated. Thus, if there were dominant protective, neutral, and dominant true-susceptibility alleles present, the neutral alleles will be mathematically equivalent to recessive susceptibility alleles and this would be mathematically equivalent to a combination of recessive and dominant true-susceptibility alleles at this location. If there were only protective and true-susceptibility alleles present, the situation would be equivalent to having recessive true-susceptibility alleles at this location.

Finally, it is also possible that certain alleles might interact with each other in other ways and that, potentially, these interactions could be quite complex. However, regardless of their complexity, such interactions, again, should be reflected by a change in the apparent penetrance of specific genotypes, in the requirement for more loci to be in "susceptible allelic states" in order to produce susceptibility, or in a mixture of dominance. As a result, it is sufficient to consider in the Model only the "susceptible allelic states" of dominant, recessive, and mixed dominance outlined above.

2. Relationships between x, r, and n

Let **x**, and **n** be positive integers such that (**x** ≥ **n**) and let (r > 0).

If C ($0 < C \leq C^*$) is the proportion of patients who are both susceptible to MS and HLA DRB1*1501-negative in the population, where [$C^* = C / (0.76) < 1$], and where the expected "frequency of susceptibility" (F) at the non-HLA DRB1 loci is (h/r); then, as in Equation (43) earlier:

$$[(x)(x-1)\cdots(x-n+1)] / [(x+r)(x+r-1)\cdots(x+r-n+1)] = C^* < 1$$

Dividing the numerator and denominator by (r^n), the limit of this expression, as ($r \to \infty$), is:

$$[(x/r)(x/r - 1/r)\cdots(x/r - n/r + 1/r)] / [(x/r + 1)(x/r + 1 - 1/r)\cdots(x/r + 1 - n/r + 1/r)]$$

$$= (x/r)^n / [(x/r) + 1]^n = C^* < 1$$

or: $$\lim_{r\to\infty} (x/r) = (C^*)^{1/n} / [1 - (C^*)^{1/n}] = 1 / [(1/C^*)^{1/n} - 1] \quad (48)$$

Thus, the ratio of (x/r) is a constant (in the limit) for any given **n**.

There are also other constraints. Thus, because the "frequency of susceptibility" can never exceed 100%, it must be the case that:

$$(h/r) \leq 1.0 \quad \text{and, therefore:} \quad (1/r) \leq (1/h) = 4.17$$

In addition, as indicated above, (**x** ≥ **n**) because, otherwise, HLA DRB1*1501 would be necessary for MS susceptibility.

Earlier, in Equation (43), only the circumstance, in which ($Pt_0 = Pt_1$), was considered. Clearly, however, if ($Pt_0 \neq Pt_1$), the observed proportion of individuals in the MS population who are HLA DRB1* 1501-negative (C_{obs}) will differ from the true proportion (C) of susceptible individuals. Thus, more generally, the observed proportion of HLA DRB1* 1501-negative individuals (C_{obs}) will be:

$$C_{obs} = \big[[(C)(Pt_0)] / [(C)(Pt_0)+(1-C)(Pt_1)]\big]$$

After some rearrangement, this becomes:

$$[(Pt_1 / Pt_0)] = [(C) / (1- C)] / [(C_{obs}) / (1- C_{obs})] \qquad (49)$$

In other words, the penetrance ratio (Pt_1 / Pt_0) is equal to the ratio of the true odds that a susceptible individual is HLA DRB1*1501-negative to the observed odds ratio that an individual with MS is HLA DRB1*1501-negative. In this case, the greater the proportionate penetrance of the HLA DRB1*1501 genotype, the greater the actual value of C relative to C_{obs}.

The relationship between (x/r) in the limit and (**n**) in Equation (48) can also be determined. Thus, letting (m > 0) be an arbitrary constant, then the ratio between the limiting value of (x/r) when (**n** = m·t) to that when (**n** = t) is:

$$(x/r)_{mt} / (x/r)_t = [(1/C^*)^{1/t} - 1] / [(1/C^*)^{1/mt} - 1] \qquad (50)$$

where $(x/r)_{mt}$ and $(x/r)_t$ are the limiting values of (x/r) at the two levels of (**n**).
Because both the numerator and the denominator tend to 0 as ($t \to \infty$), the limiting value of:

$$\lim_{t\to\infty} [(1/C^*)^{1/t} - 1] / [(1/C^*)^{1/mt} - 1] = 0 / 0$$

which is indeterminate. However, by l'Hôpital's rule, the limit of the ratio of the derivatives of two functions is equal to the limit of the ratio of the functions themselves under these circumstances, so that:

$$\lim_{t\to\infty} [(1/C^*)^{1/t} - 1] / [(1/C^*)^{1/mt} - 1] = \lim_{t\to\infty} (m)[(C^*)^{(m-1)/t}] = m \qquad (51)$$

Thus, even though, at a particular value of C* (e.g., $C^* = C_0^*$), both (x/r) and (**n**) tend toward to infinity with increasing values of **n**, the relationship between them, in the limit, is linear.

Thus, in the limit:

$$(x/r) = k\mathbf{n} \qquad (52)$$

where k is the slope of the line relating the two variables. Moreover, if we let (C_0^*) represent the value of C* when ($Pt_0 = Pt_1$), and if we let C_1^* be the value of C* at some point where

($Pt_0 \neq Pt_1$), then, for some (**b** > 0) and for the value of C* determined by Equation (43), we can define:

$$C_1^* = (C_0^*)^{1/\mathbf{b}} \qquad (53)$$

where: $\mathbf{b} = [\ln(C_0^*)] / [\ln(C_1^*)]$

Moreover, because: $(C_1^*)^{1/\mathbf{n}} = [(C_0^*)^{1/\mathbf{b}}]^{1/\mathbf{n}} = (C_0^*)^{1/\mathbf{bn}}$

then, by Equation (53):

$$\lim_{n\to\infty} [(1/C_0^*)^{1/\mathbf{n}} - 1] / [(1/C_0^*)^{1/\mathbf{bn}} - 1] = \mathbf{b} \qquad (54)$$

so that (x/r) = k**bn**, where **b** represents the change in slope of the line that follows from Equation (53) based on the movement of C* from C_0^* to C_1^* due to a change in the ratio of (Pt_1 / Pt_0). The slope of the new line is (k**b**), so that in order to determine its value one needs to calculate both (k) and (**b**). However, if, instead of picking C_0^* at (C_{obs} / [0.76]), we pick a reference value such that:

$$C_0^* = (1/e) = 0.368$$

then the slope of the line (k) at C_0^* following from Equation (52) is equal to (1) and, thus, the value of (**b**) represents the slope of the line at any value of C_1^*. Using this as the reference, the slope of the line at ($C_1^* = C_{obs}/(0.76) = 0.59$), in the limit, is:

$$\mathbf{b} = [\ln(C_0^*)] / [\ln(C_1^*)] = \ln(1/e) / \ln(0.59) = (-1) / (-0.52) = 1.9 \qquad (55)$$

3. Prevalence and Concordance

The prevalence (probability) of MS in the general population converges in the limit. Thus, re-stating Equation (45):

$$P(MS) = \left(\sum_{i=0}^{1} [(Pt^*)][(1)!/(i)!\,(1-i)!][(h)^{i}(1-h)^{1-i}]\right) \cdot$$

$$\left[\,1 - \left(\sum_{j=0}^{n-i-1} [(x)!\,/\,(x-j)!\,(j)!]\,[\,(h/r)^{j}\,(1-h/r)^{x-j}]\right)\right]$$

Thus, in Equation (45), for any fixed (i and n), the expression:

$$\sum_{j=0}^{n-i-1} [\,(x)!/(x-j)!(j)!][(h/r)]^{j}[(1-h/r)]^{x-j}$$

$$= \sum_{j=0}^{n-i-1} [\,[x/r - n/r]\cdots[x/r - n/r - j/r + 1/r]\,/\,(j)!]\,[(h)]^{j}\,[(1-h/r)]^{x-j}$$

As ($r \to \infty$), this expression becomes:

$$= \sum_{j=0}^{n-i-1} \left[[(x/r)^{j}\,/\,(j)!][(h)]^{j}\,[(1-(h/r)]^{(x/r)(r)}\right] \tag{56}$$

By Equation (48), the expression $[(x/r)^{j} / (j)!][(h)]^{j}$ is constant for any given n, h, and j.

Moreover, because

$$\lim_{r \to \infty} (1-a/r)^{br} = e^{-ab} \tag{57}$$

the expression $[(1-(h/r)]^{(x/r)(r)}$ is also a constant (in the limit) and is equal to $e^{-(x/r)(h)}$.

Thus, as $(r \to \infty)$, P_{MS} approaches the limit:

$$P(MS) = \left(\sum_{i=0}^{1} [(Pt^*)][(1)!/(i)!\,(1-i)!][(h)^{i}(1-h)^{1-i}]\right) \cdot \left[1 - \left(\sum_{j=0}^{n-i-1} (x/r)^{j} / (j)!][(h)^{j}][e^{-(x/r)(h)}]\right) \right] \qquad (58)$$

which is a finite constant for any fixed **n** and h.

Moreover, in the last expression, in the limit for the summation over (j) is equivalent to partial summations of a Poisson distribution. Thus, for the summation over (j), this becomes:

$$= \sum_{j=0}^{n-i-1} (\lambda^{j} e^{-\lambda}) / (j)!, \text{ where } \lambda = (x/r)(h)$$

Because, in the limit, $(x/r) = b\mathbf{n}$ (from Equation [55] and using the reference $C_0^* = e^{-1}$), the equation for mean of the Poisson distribution (λ) becomes:

$$\lambda = (\mathbf{b}\cdot h)(\mathbf{n}) = (0.456)(\mathbf{n}); \quad \text{at } (C_1^* = 0.59) \text{ and } (h = 0.24) \qquad (59)$$

It is noteworthy, however, that, in the limit, for some constant $(c > 0)$, as $(\mathbf{n} \to \infty)$,

$$\lim_{n \to \infty} \sum_{k=0}^{n} (c\mathbf{n})^{k} e^{-c\mathbf{n}} / (k)! = 0.5 \text{ ; when } (c = 1) \qquad (60)$$

The point $(c = 1)$ represents a divide for these summations. Thus, if $(c < 1)$ each sum (in the limit) will increase monotonically to (1). By contrast, if $(c > 1)$ this sum will decrease monotonically to (0). This has implications for the possible values that (Pt_1/ Pt_0) can take. Thus, because the slope (**b**) increases as the ratio of (Pt_1/ Pt_0) increases, this means the constant term $(c = \mathbf{b}h)$ in Equation (59), which is less than 1 when $(Pt_1 = Pt_0)$, will ultimately exceed 1 as (Pt_1/Pt_0) increases. At the point where this crossover occurs, the anticipated prevalence of MS from Equation (58) will increase with increasing values of (**n**), approaching a limit greater than 0.1 - 0.2% and, thus, will never be compatible with the prevalence in the general population.

This crossover occurs when ($\mathbf{b}\cdot h = 1$) or when ($\mathbf{b} = 4.17$) or, from Equation (54), at ($C_1^* = 0.79$; or: $C_1 = 0.60$).

Based on Equation (50), therefore, ($Pt_1/Pt_0 < 1.8$) and, with an average penetrance of 25% from Equation (44), this would mean that ($Pt_1 \leq 0.32$ and $Pt_0 \geq 0.18$) in monozygotic-twins. These derived limits fit well with the experimental observations from Canada (11) where the derived concordance rates for MS in HLA DRB1*1501-positive and HLA DRB1*1501-negative monozygotic-twin probands are approximately equal (Table 3).

Similarly, the concordance rate for MS in siblings (not identical twins), with the proband being HLA DRB1* 1501-negative, also approaches a limit as ($r \rightarrow \infty$). Thus, for any fixed values of (i, j, k, m, and n), in summation over (p) in Equation (47) and for the conditions in which ($i + j + k + m \leq \mathbf{n} - 1$), in the limit, Equation (47) becomes:

$$P(MS_{H-}) = \left(\sum_{i=0}^{1} [(Pt^*)][(1)!/(i)!\,(1-i)!][(P_{h1})^{i}(1-P_{h1})^{1-i}]\right)\cdot$$
$$\Big[\Big(1 - \sum_{j=0}^{n-i-1} [(n_1)!/(n_1-j)!(j)!][(P_{A1})^{j}(1-P_{A1})^{n1-j}]\Big)\cdot$$
$$\Big(\sum_{k=0}^{n-i-j-1} [(n_2)!/(n_2-k)!(k)!][(P_{A2})^{k}(1-P_{A2})^{n2-k}]\Big)\cdot$$
$$\Big(\sum_{m=0}^{n-i-j-k-1} [(n_3)!/(n_3-m)!(m)!][(P_{A3})^{m}(1-P_{A3})^{n3-m}]\Big)\cdot$$
$$\Big(\sum_{p=0}^{n-i-j-k-m-1} [(x/r)^{p}/(p)!][(h)^{p}][e^{-(x/r)(h)}]\Big)\Big]$$

In this equation, $P_{A1} = [(0.5)(1 + 2a_1 - (a_1)^2)]$ and, because ($a_1 \rightarrow 0$) in the limit, this expression becomes: $P_{A1} = [0.5]$. Similarly, in the limit, $P_{A2} = [0.25]$ and $P_{A3} = [0.5]$. Therefore, by Equations (48) and (50) above, $P(MS_{H-})$ is a constant under these conditions.

Similarly, Equation (46) becomes:

$$P(MS_{H+}) = \left(\sum_{i=0}^{1} [(Pt^*)][(1)!/(i)!\,(1-i)!][(P_H)^i (1-P_H)^{1-i}]\right) \cdot$$

$$\left[\left(1 - \sum_{j=0}^{n-i-1} [(n_1)!/(n_1-j)!(j)!][(P_{A1})^j (1-P_{A1})^{n1-j}]\right) \cdot\right.$$

$$\left(\sum_{k=0}^{n-i-j-1} [(n_2)!/(n_2-k)!(k)!][(P_{A2})^k (1-P_{A2})^{n2-kj}]\right) \cdot$$

$$\left(\sum_{m=0}^{n-i-j-k-1} [(n_3)!/(n_3-m)!(m)!][(P_{A3})^m (1-P_{A3})^{n3-m}]\right) \cdot$$

$$\left.\left(\sum_{p=0}^{n-i-j-k-m-1} [(x/r)^p / (p)!][(h)^p][e^{-(x/r)(h)}]\right)\right]$$

which also a constant under comparable conditions.

In addition, for any fixed values of (i, j, k, and m), in summation over (p) in Equation (47), where [$P_{a1} = P_{a2} = P_{a3} = (h/r)$], and for conditions ($i + j + k + m \leq \mathbf{n} - 1$), the expression:

$$\sum_{p=0}^{n-i-j-k-m-1} [(x-n)!/(x-n-p)!(p)!][(h/r)]^p[(1-h/r)]^{x-p}$$

$$= \sum_{p=0}^{n-i-j-k-m-1} [[x/r - n/r]\cdots[x/r - n/r - p/r + 1/r] / (p)!]\,[(h)]^p\,[(1-h/r)]^{x-p} \qquad (61)$$

Which by Equations (53), (54), and (55) in the limit as ($\mathbf{n}\rightarrow\infty$), becomes:

$$= \sum_{p=0}^{n-i-j-k-m-1} [[n(b - 1/r)]\cdots[n(b - 1/r) - p/r + 1/r] / (p)!]\,[(h)]^p\,[(1-h/r)]^{x-p} \qquad (62)$$

As (**r**) ranges from 1 to 0.24, in Equation (61), the first factor [($\mathbf{x}/\mathbf{r} - \mathbf{n}/\mathbf{r}$)] in each summand will become (0) at the point where ($\mathbf{x} = \mathbf{n}$) and, at this point of transition, the summation itself will

equal (0). However, because the ratio (**x**/**r**) approaches its limit of (**bn**) from above, the actual transition will occur closer to the point (**bn**). Thus, in Equation (62), as (**n**) increases and as (**x**/**r**) approaches (**bn**), this factor becomes equal to [**n**(**b** - 1/**r**)], this transition will occur closer to the point (**b** = 1/**r**) or, by Equation (55), when (**r** = 1/**b** = 1/1.9 = 0.53). Thus, as (**n**) increases (and, therefore as **x** increases), for all values of (**r** ≤ 0.53) the summation will increasingly approach 0 (more quickly for values of **r** closer to 0.24) and, by Equations (46) and (47), the probability of susceptibility for these genotypes will approach 1. Similarly, as the value of (**r**) approaches 0.53 from above, the value of the summation will approach 0 although, even in the limit, it will be greater than 0 for all (**r** > 0.53).

4. Susceptibility Stratified by HLA DRB1*1501 Status

In the general population, susceptible genotypes that include the HLA DRB1*1501 allele, increase the odds of MS developing far out of proportion to the increase that occurs with other susceptible genotypes. For example, in a population of MS patients, the odds of randomly picking HLA DRB1*1501 allele from among all of the HLA DRB1 alleles is much greater than the same odds in the general population. Using the data from UCSF (J Oksenberg, personal communication) for the HLA DRB1*1501 allele (i.e., $h_m = 0.556$ and $a_{hm} = 0.328$), the odds ratio (OR) and is:

$$OR = [(a_{hm}) / (1- a_{hm})] / [(a_h) / (1- a_h)]$$

$$= [(0.328) / (0.672)] / [(0.128) / (0.872)] = 3.3$$

Similarly, calculating (from Table 2) the ratio of the odds for possession of a single copy or a double copy of this allele in the MS population (h_{ms} and h_{md}, respectively) compared to the same odds in the general population (h_s and h_d, respectively) yields:

$$\text{OR (single copy)} = [(h_{ms}) / (1- h_{ms})] / [(h_s) / (1- h_s)]$$

$$= [(0.45) / (0.55)] / [(0.224) / (0.776)] = 2.83$$

$$\text{OR (double copy)} = [(h_{md}) / (1- h_{md})] / [(h_d) / (1- h_d)]$$

$$= [(0.10) / (0.90)] / [(0.016) / (0.984)] = 6.83$$

These numbers give an estimate of the strength of this particular association.

Using the terminology from Section 1, and re-expressing Equation (2) to include the HLA DRB1 locus for the probability of having (**n**) or more susceptible states at the (x+1) susceptibility loci in the general population is:

$$P[y_n] = \left(\sum_{i=0}^{1} [(1)!/(i)!\,(1-i)!][(h)^i (1-h)^{1-i}]\right) \cdot \left(\sum_{k=n-i}^{x} [(x)!\,/\,(x-k)!\,(k)!]\,[\,(h/r)^k\,(1-h/r)^{x-k}]\right)$$

which can be expanded as:

$$P[y_n] = \left(\sum_{i=0}^{1} [(1)!/(i)!\,(1-i)!][(h)^i (1-h)^{1-i}]\right) \cdot \left(\sum_{j=0}^{1} [(x)!\,/\,(x-j)!\,(j)!]\,[\,(h/r)^j\,(1-h/r)^{x-j}]\right) \cdot \left(\sum_{k=n-i-j}^{x-1} [(x)!\,/\,(x-k)!\,(k)!]\,[\,(h/r)^k\,(1-h/r)^{x-k}]\right) \quad (63)$$

As discussed in the development of Equation (9), the probability of genetic susceptibility in the general population (P[n]) is defined as:

$$E(P[y_i]) \approx P[n] = P[y_n] \quad (64)$$

As indicated in Table 1 and by Equations (3) and (44), (Pt*) is defined such that:

$$P(MS) = (Pt^*)(P[y_n]) \quad (65)$$

Using Equations (36) and (65), and letting ($Pt_0 = Pt_1 = Pt^*$), the probability that an individual in the general population is both genetically susceptible to MS and carries the HLA DRB1*1501 allele (P_{hm}) as:

$$P_{hm} = (h_m)(P[n]) \leq (2)(h_m)[P(MS) / Pt^*]$$

Substituting into this equation the epidemiological observations of:

$$P(MS) \approx 0.0015; \quad (Pt^* = 0.134); \quad \text{and:} \quad (h_m = 0.55),$$

then: $P_{hm} \approx (2)(0.55)(0.0112) = 0.0124$

Considering the prevalence of having at least one copy of HLA DRB1*1501 in the general population (h), the likelihood that an individual who carries this allele is actually susceptible to getting MS ($P[S]_{HLA}$) is:

$$P[S]_{HLA} \leq (P_{hm}) / (h) = 0.0124 / 0.24 = 5.2\% \qquad (66)$$

This estimate is higher than the one made previously (10) because, those calculations, used the actual concordance (CR_{MZ}) rather than the adjusted concordance (CR_{IG}), which takes into account the shared intra-uterine and similar post-natal environments of twins. For the same reason, the estimated prevalence of susceptibility in the general population is:

$$P(MS) / Pt^* \leq 2.2\%$$

which is also higher than that estimated earlier (10).

By contrast, letting (P_{nhm}) be the probability that an individual in the general population is both genetically susceptible to MS and does not carry the HLA DRB1*1501 allele, then:

$$P_{nhm} = (1 - h_m)(P_{MS} / Pt^*) = (0.45)(0.0112) = 0.0050$$

and the likelihood of being susceptible to getting MS for an individual who doesn't carry this allele ($P[S]_{HLA-}$) is:

$$P[S]_{HLA-} = (1 - P_{nhm}) / (1 - h) = 0.0050 / 0.76 = 0.7\% \qquad (67)$$

From Equations (66) and (67), it is apparent that individuals who carry the HLA DRB1*1501 allele are (3.94) times as likely to be susceptible to getting MS than those who don't. Importantly, however, this observation is ***not*** only a reflection of the increased likelihood of

susceptibility due to possession of this allele. It is also due, in part, to the fact that, because the HLA DRB1*1501 allele is known to be a true susceptibility allele, possession of this allele provides information about the group who carry it. By contrast, no such information is available for the group who don't carry it. Thus, if the general population were to be stratified on the basis of another true susceptibility allele, the likelihood of susceptibility would also be increased in those that possess this allele.

In order to help disentangle these possibilities, therefore, it will be useful to define two other functions. Thus, in Equation (63), for some integer (t), we will define the functions:

$$P'[n-t] \;=\; \left(\sum_{k=n-t}^{x} [(x)! / (x-k)!\,(k)!]\,[\,(h/r)^{k}\,(1-h/r)^{x-k}]\,\right)$$

and:

$$P''[n-t] \;=\; \left(\sum_{k=n-t}^{x-1} [(x)! / (x-k)!\,(k)!]\,[\,(h/r)^{k}\,(1-h/r)^{x-k}]\,\right)$$

Using these relationships, the HLA DRB1 locus can be expanded out of Equation (63) such that:

$$P[y_n] \;=\; P[n] \;=\; (1-h)(P'[n]) + (h)(P'[n-1]) \qquad (68)$$

and, from Equation (65):

$$P(MS) = (Pt^*)(P[n]) \;=\; (Pt^*)\big[(1-h)(P'[n]) + (h)(P'[n-1])\big] \qquad (69)$$

Because (**n**) is similar to an average number of necessary loci, Equations (68) actually asserts only that the two summands add to the "average" value. Thus, if we define (b) and (c) to be unknown constants we can rewrite Equation (68) to be:

$$P[n] \;=\; (b)(1-h)(P'[n]) \;+\; (c)(h)(P'[n-1]) \qquad (70)$$

Expanding the non-HLA locus in Equation (70) yields:

$$P[n] \;=\; (1-h)(b)\big[(1-h/r)(P''[n]) + (h/r)(P''[n-1])\big]$$
$$+ (h)(c)\big[(1-h/r)\,(P''[n-1]) + (h/r)(P''[n-2])\big] \qquad (71)$$

If we define [P_{HM}] as the probability that a susceptible individual in the general population carries the HLA DRB1*1501 allele, and [F_M] as the probability that a susceptible individual in the general population is in a susceptible allelic state at a specific non-HLA DRB1 locus, then:

$$(1 - h)(b)\big[(1 - h/r)(P''[n]) + (h/r)(P''[n\text{-}1])\big] \quad = \quad (1 - P_{HM}) \qquad (72)$$

$$(h)(c)\big[(1 - h/r)(P''[n\text{-}1]) + ((h/r)(P''[n\text{-}2])\big] \quad = \quad (P_{HM}) \qquad (73)$$

$$(1 - h/r)\big[1 - h)(b)(P''[n]) + (h)(c)(P''[n\text{-}1])\big] \quad = \quad (1 - F_M) \qquad (74)$$

$$(h/r)\big[(1 - h)(b)(P''[n\text{-}1]) + (h)(c)(P''[n\text{-}2])\big] \quad = \quad (F_M) \qquad (75)$$

where, if ($Pt_1 \approx Pt_0$), then ($P_{HM} \approx h_m$ and $F_M \approx F_m$). Using Equation (69) and expanding yields:

$$P(MS) = (Pt^*)\big[(1 - h)(b)\big[(1 - h/r)(P''[n]) + (h/r)(P''[n\text{-}1])\big]$$

$$+ (h)(c)\big[(1 - h/r)(P''[n\text{-}1]) + (h/r)(P''[n\text{-}2])\big]\big] \qquad (76)$$

From Equation (76), it is apparent that one interpretation of these two constants is that they reflect a different penetrance for susceptible genotypes with and with out the HLA DRB1*1501 allele. Therefore, from this perspective:

$$Pt_0 = (Pt^*)(b)$$

and: $$Pt_1 = (Pt^*)(c)$$

Obviously, if ($b = c = 1$), then ($Pt_1 = Pt_0$) and, thus, ($P_{HM} = h_m$ and $F_M = F_m$). However, under conditions where ($Pt_1 \approx Pt_0$), as suggested by Table 2, but where ($b \neq c \neq 1$), the meaning of these constants has a different interpretation. In fact, as shown in Table 9, substituting the known or derived values for (**n**), (**r**), (**x**) and (h), into Equation (63) and using Equation (73) to calculate ($P_{HM} = h_m$) at ($b = c = 1$), gives a figure of ($h_m \approx 0.43$), which underestimates its observed value (i.e., $h_m = 0.55$). Importantly, the degree to which (h_m) is underestimated for specific values of (**n**), (**r**), and (**x**) actually provides a means to estimate the values of the unknown constants (b)

and (c) in different circumstances and, in fact, from Equation (71) it is clear that in order to increase the estimate of (h_m) from (~0.43) to (0.55) requires that ($b < 1.0$) and ($c > 1.0$).

Moreover, because for all integers ($t > 0$):

$$P[y_{n+t}] < P[y_n]$$

and:
$$P[y_{n-t}] > P[y_n]$$

and, because ($b < 1.0$) and ($c > 1.0$), it follows from Equation (70) that, for some pair of integers (t_1) and (t_2):

$$1 - P_{HM} = (1-h)\big[(b)(P'[n])\big] = (1-h)(P'[(n+t_1)]) < (1-h)(P'[n]) \qquad (77)$$

and:
$$P_{HM} = (h)\big[(c)(P'[n-1])\big] = (h)(P'[(n-t_2)-1]) > (h)(P'[n-1]) \qquad (78)$$

From Equation (64) and from the Section 1 of the main paper, (**n**) is the integer that most nearly approximates the equality:

$$P'[n-1] \approx E(P'[y_{i-1}])$$

so that, in Equations (77) and (78), the integers (t_1) and (t_2) are those that most nearly approximate the equalities:

$$P'[(n+t_1)] \approx E(P'[y_{i+t1}])$$

$$P'[(n-t_2)-1] \approx E(P'[(y_{i-t1-1}])$$

Consequently, the fact that ($b < 1.0$) and ($c > 1.0$), and the fact that ($Pt_1 \approx Pt_0$), imply that the susceptibility genotypes which carry the HLA DRB1*1501 allele are require fewer susceptibility loci to be in a susceptible state compared to susceptibility genotypes that don't carry this allele. Moreover, ($P'[n + t_1]$) and ($P'[n - t_2 - 1]$) can be calculated for different integer values for specific combinations of (**n**), (**r**), (**x**) and (h).

As a result, using the best approximation for (t_1) and (t_2) in the following two equations:

$$P'(n + t_1) / P'(n) = b \quad (79)$$

$$P'(n - t_2 - 1) / P'(n - 1) = c \quad (80)$$

provides a method to estimate the difference in the number of required loci for each of these two susceptibility states.

Clearly, the inclusion of the two constant terms in Equation (70) impacts the expected odds ratio for the HLA DRB1*1501 allele in MS, raising it from the expected $(OR \approx 2.2)$ under conditions $(c = b = 1)$ to the observed $(OR = 3.3)$ under conditions where $(c > 1 > b)$. Similarly, the inclusion of these two constants will alter the expected odds ratio for the non-HLA DRB1 loci because the constants (b) and (c) also appear in Equations (74) and (75). For a dominant (a_{1m}) or a recessive (a_{2m}) non-HLA allele in a susceptible population, the anticipated odds ratios are:

$$OR = [(a_{1m}) / (1 - a_{1m})] / [(a_1) / (1 - a_1)]$$

$$OR = [(a_{2m}) / (1 - a_{2m})] / [(a_2) / (1 - a_2)] \quad (81)$$

However, unlike the circumstance for the susceptible genotypes that carry the HLA DRB1*1501 allele, the odds ratio is only slightly altered by inclusion of these constant terms in the case of the non-HLA locus because they tend to offset each other. Thus, approximately half of the genotypes that have the non-HLA locus in a susceptible state will also carry the HLA DRB1*1501 allele and, as indicated by Equations (74) and (75) the multiplier for this locus will be a weighted average of both the (b) and (c) terms.

Another possibility worth considering is the circumstance in which those genotypes homozygous for HLA DRB1*1501 either have greater penetrance than or require fewer susceptibility loci to be in a susceptible state compared to those genotypes with only one or no copies of this allele. Indeed, in the UCSF experience (i.e., $h_m = 0.556$ and $a_{hm} = 0.328$) from Table 2, it is apparent that the homozygous persons in an MS population are increased by a factor of (6.06) whereas heterozygous persons are only increased by a factor of (2.04). The ratio of these two factors is (0.34). If a dominant susceptibility allele has a weighting function for

resulting in MS of (1, 1) for the heterozygous and homozygous states respectively, then the weighting function for a recessive allele is (0, 1). In the case of the HLA DRB1*1501 allele the observed weighting function is (0.34, 1) so that, although this locus confers susceptibility in a dominant fashion, the homozygous state results in MS much more frequently than the heterozygous state. In this sense, then, this locus appears more recessive than dominant. Again there are two possibilities. Either the homozygous state has a greater penetrance than the heterozygous state or it requires fewer loci to be in a "susceptible allelic state" to produce susceptibility. Because there are no data about the relative penetrance of the homozygous and heterozygous states, the issue cannot be settled. Regardless, however, the values of (b) and (c) are unchanged, so that, if this circumstance is not exclusively the result of a penetrance difference, the value of (t_2) in the expression [$P'(n - t_2 - 1)$] in Equation (80) will be increased for the homozygous state and decreased for the heterozygous state. Even, so, this difference in (t_2) is still amounts to no more that 1 or 2 loci.

5. Odds Ratios for Polymorphic Alleles or Multiple Genes at Susceptibility Loci

Polymorphic Alleles

Because a susceptibility locus (i.e., haplotype), typically includes several distinct genes and because individual genes are often quite polymorphic, either or both of these complications may alter the familial recurrence rates in different conditions. For example, the possibility of polymorphic alleles

If there is only one "dominant" susceptibility allele for the single susceptibility gene at a particular susceptibility locus, then the probability that a random individual in the population will be in a "susceptible allelic state" (P_{a1}) at this location is:

$$P_{a1} = 2(a_1) - (a_1)^2 = (h/r)$$

the expected allelic frequency, in this circumstance, will be [$a_1 = 1 - (1 - h/r)^{1/2}$]. If (g) "dominant" alleles were present (a_{1i}; for i = 1 to g; with $E[a_{1i}]$ = "a_1"; where "a_1" is the apparent allelic frequency), this equation would be unchanged, reflecting simply the sum of the frequencies ["a_1" = $(g)(a_1)$] of these different "dominant" alleles. Thus,

$$P_{a1} = 2(a_{11} + a_{12} + \ldots + a_{1g}) - (a_{11} + a_{12} + \ldots + a_{1g})^2$$

$$= 2[(g)(a_1)] - [(g)(a_1)]^2 = (h/r)$$

If there is only one "recessive" susceptibility allele for the single susceptibility gene at a particular susceptibility locus, then the probability that a random individual in the population will be in a "susceptible allelic state" (P_{a2}) at this location will be:

$$P_{a2} = (a_2)^2 = (h/r)$$

Therefore, the expected allelic frequency, in this circumstance, will be [$a_2 = (h/r)^{1/2}$]. If (g) "recessive" alleles were present (a_{2i}; for i = 1 to g; with $E[a_{2i}]$ = "a_2"; where "a_2" is the apparent allelic frequency), this equation would be depend upon whether or not the heterozygous

states $[(a_{2i})(a_{2j});\ i, j = 1 \text{ to } g;\ i \neq j]$ conferred susceptibility. If they did, the sum of allelic frequencies would be equivalent to a single allelic trait with an apparent allelic frequency of $[\text{“}a_2\text{”} = (g)(a_2)]$. By contrast, if the heterozygous states (i.e., a person with two recessive alleles but not both the same allele) did not confer susceptibility, the equation would be altered such that:

$$(a_{21} + a_{22} + \ldots + a_{2g})^2 = [(g)(a_2)]^2 = \sum_{i=1}^{g} (a_{2i})^2 + 2 \sum_{i=1,\, i<j}^{g} (a_{2i})(a_{2j})$$

and, omitting the heterozygote term, would yield:

$$P_{a2} = \sum_{i=1}^{g} (a_{2i})^2 = (g)(a_2)^2 = (h/r)$$

so that the apparent allelic frequency is $[\text{“}a_2\text{”} = (g^{1/2})(a_2)]$. If the apparent allelic frequency (“a_1” and “a_2”) at a non-HLA susceptibility locus is increased in an MS population by a factor of (m) compared to the general population, the Equations (81) can be rewritten for the apparent allelic frequencies as:

$$\begin{aligned} OR &= [(m \cdot \text{“}a_1\text{”})(1 - \text{“}a_1\text{”})] / [(\text{“}a_1\text{”})(1 - \{m \cdot \text{“}a_1\text{”}\})] \\ &= (m) \cdot [(1 - \text{“}a_1\text{”}) / (1 - \{m \cdot \text{“}a_1\text{”}\})] \qquad (82) \\ OR &= (m) \cdot [(1 - \text{“}a_2\text{”}) / (1 - \{m \cdot \text{“}a_2\text{”}\})] \end{aligned}$$

for dominant (“a_1”) and recessive (“a_2”) genes respectively. If there are $(g > 1)$ susceptibility alleles present and, if the heterozygous states confer susceptibility for recessive alleles, these equations become:

$$\begin{aligned} OR &= [(m \cdot \text{“}a_1\text{”}/g) / (1 - \{m \cdot \text{“}a_1\text{”}/g\})] / [(\text{“}a_1\text{”}/g) / (1 - \text{“}a_1\text{”}/g)] \\ &= (m) \cdot [1 - \text{“}a_1\text{”}/g] / [1 - (m \cdot \text{“}a_1\text{”}/g)] \qquad (83) \\ OR &= (m) \cdot [1 - \text{“}a_2\text{”}/g] / [1 - (m \cdot \text{“}a_2\text{”}/g)] \end{aligned}$$

or, if the heterozygous states do not confer susceptibility for recessive alleles, then:

$$OR = (m) \cdot [1 - \text{“}a_1\text{”} / g^{1/2}] \; / \; [1 - (m \cdot \text{“}a_1\text{”} / g^{1/2})]$$

Equation (83) can be rewritten such that:

$$OR = (m) \cdot [(g-1) + (1 - \text{“}a_1\text{”})] \; / \; [(g - 1) + (1 - \{m \cdot \text{“}a_1\text{”}\})]$$

and because ($g > g^{1/2} > 1$), the odds ratios, which are calculated from those Equations that include the factor ($g > 1$), as in Equation (83), are always less than that the odds ratio calculated for the apparent allelic frequency (“a_1” and “a_2”) as in Equation (82).

Importantly, however, although increasing the number of susceptibility alleles and a single susceptibility gene at a particular susceptibility locus affects the calculated “apparent” odds ratio compared to calculations for true alleles, this doesn’t affect the familial recurrence calculations for dominant alleles (i.e., the sibling still either does or doesn’t inherit the dominant allele from the parent who has it. By contrast, for recessive alleles, the situation would be different. For example, the calculated probability (P_{A2}) in non-twin siblings, if the heterozygous state didn’t confer susceptibility, would be:

$$P_{A2} = [0.25][1 + a_2]^2 < [0.25][1 + \text{“}a_2\text{”}]^2$$

Naturally, the predicted prevalence of MS in the general population would not be affected by any of these considerations because the frequency of susceptibility at each non-HLA locus (h/r) is the same regardless of the number of alleles that comprise this susceptibility.

Multiple Genes

It is also possible that a single susceptibility locus might harbor more than one susceptibility gene among the collection of genes in the haplotype. In this case, the terms:

$$(a_{11}, a_{12}, \ldots a_{1g}) \quad \text{and} \quad (a_{21}, a_{22}, \ldots a_{2g})$$

refer to dominant and recessive susceptibility alleles in the (g) different genes at one susceptibility locus. If each allele is dominant (i.e., a single copy confers susceptibility regardless of the state at the other [g - 1] genes), then the apparent allelic frequency (“a_1”) at this locus will still be defined by:

$$P_{a1} = 2(a_{11} + a_{12} + \ldots + a_{1g}) - (a_{11} + a_{12} + \ldots + a_{1g})^2$$

$$= 2[(g)(\text{“}a_1\text{”})] - [(g)(\text{“}a_1\text{”})]^2 = (h/r)$$

Similarly, if each allele is recessive (i.e., two copies of the susceptibility allele in one gene confers susceptibility regardless of the state at the other [g - 1] genes), then the apparent allelic frequency (“a_2”) will still be defined by:

$$P_{a2} = (g)(\text{“}a_2\text{”})^2 = (h/r)$$

However, in the circumstance where several susceptibility genes are present at a single locus, a mixture of dominant and recessive alleles or interactions between the different susceptibility genes, will likely make the situation quite complicated. Nevertheless, as above for polymorphic recessive alleles (where heterozygotes did not confer susceptibility), these interactions and circumstances will reduce the predicted familial recurrence rates for both dominant and recessive alleles, and they will lower the observed odds ratios, but they will not impact the predicted prevalence because, again, the frequency of susceptibility at each non-HLA locus (h/r) is the same regardless of either the number of genes that comprise this susceptibility or their interactions.

Single Nucleotide Polymorphisms (SNPs)

Current genome-wide association scans use SNPs to identify regions of the different chromosomes that are associated with a particular illness. In general (assuming the location of SNPs is random), the DNA at a particular SNP location will typically be in one of only two states. Occasionally, a SNP is known to cause a functional change in the gene (e.g., introducing

a stop codon or a non-synonymous amino acid substitution, altering splice sites, or changing the binding characteristics of regulatory molecules) and it may well be, in these cases, that the SNP uniquely tags the allele of interest (e.g., 33, 34). However, in other cases, if there are more than two alleles for a particular susceptibility gene at a particular susceptibility locus (only some of which produce susceptibility), it is possible that any particular SNP will tag both susceptibility alleles and non-susceptibility alleles. Such a circumstance will affect the observed OR.

As an example, suppose there are 3 alleles of a single susceptibility gene at a particular susceptibility locus. as shown pictorially below.

	susceptibility locus	SNP locus
Allele NS_1	……...T………..……………………G……………..	
Allele NS_2	……...T……..……………………C…………….	
Allele S_3	……...A……..……………………C…………….	

Further suppose that one of these alleles (S_3), with an allelic frequency of (s) produces a susceptible state at this locus whereas the other two alleles (NS_1 and NS_2), each with an allelic frequency of (ns), do not. Suppose also that the DNA at a particular SNP location is in one state on both the S_3 and the NS_2 alleles but in another state on the NS_1 allele and finally, suppose that the allelic frequency of the S_3 allele is increased by a factor of (**m**) in a population of MS patients. In this example, the true OR would be:

$$OR = [(\mathbf{m} \cdot s) / (1 - \{\mathbf{m} \cdot s\})] / [(s) /(1 - s)]$$

Whereas the observed odds ratio would be:

$$OR = [(ns_m + \mathbf{m} \cdot s) / (1 - \{ns_m + \mathbf{m} \cdot s\})] / [(ns + s) /(1 - \{ns + s\})]$$

where ($ns_m = [1 - \mathbf{m} \cdot s] /2$).

In the circumstance of a dominant allele, similar to the HLA DRB1*1501 allele, where:

$(s = 0.128)$, $(\mathbf{m} \cdot s = 0.324)$, $(ns = 0.436)$, and $(ns_m = 0.336)$

The odds ratio will be reduced from (OR = 3.3) to:

$$OR = (0.664 / 0.336) / (0.564 / 0.436) = 1.5$$

For a less common allele (e.g., **r** = 4; and **m** unchanged) where:

$(s = 0.03)$, $(\mathbf{m} \cdot s = 0.08)$, $(ns = 0.485)$, and $(ns_m = 0.460)$

Then the odds ratio will reduced from (OR = 2.8) to:

$$OR = (0.540 / 0.460) / (0.515 / 0.485) = 1.1$$

6. TABLES

Table 1. Model Definitions

a_h	=	allelic frequency of the HLA DRB1*1501 susceptibility allele in the general population (only one copy needed for susceptibility)
a_{hm}	=	allelic frequency of the HLA DRB1*1501 susceptibility allele in the MS population (a_{hm} = 0.328 in UCSF database)
$a_{1,}\ a_{2,}\ a_3$	=	expected allelic frequency of dominant (a_1), recessive (a_2), and mixed (a_3) alleles at the non-HLA DRB1 loci in the general population
$a_{1m,}\ a_{2m,}\ a_{3m}$	=	allelic frequency of dominant (a_{1m}), recessive (a_{2m}), and mixed (a_{3m}) alleles at the non-HLA DRB1 loci in an MS population
F_i, F	=	unknown "frequency of susceptibility" (see text for definition) at the non-HLA loci in the general population (i = 1, 2,…x). [$E(F_i) = F = h/r$)]
F_m	=	"frequency of susceptibility" at a non-HLA locus in an MS population
h	=	known "frequency of susceptibility" at the HLA DRB1 locus in the general population (equal to the probability of having at least 1 copy of this allele) [$h = 2a_h - (a_h)^2 = 0.24$]
h_m	=	known "frequency of susceptibility" at the HLA DRB1 locus in the MS population (equal to the probability of having at least 1 copy of this allele) [in the UCSF dataset; $h_m = 0.55$]
P_{a1}, P_{a2}, P_{a3}	=	probability that a person in the general population has a "susceptible allelic state" (see text for definition) at dominant (P_{a1}), recessive (P_{a2}), and mixed (P_{a3}) non-HLA DRB1 loci. ($P_{a1} = P_{a2} = P_{a3} = F = h/r$)
P_{h1}	=	probability that person with an HLA-negative sibling (not an identical-twin) has at least one copy of the HLA DRB1*1501 allele

P_H	=	probability that an individual with an affected HLA DRB1*1501 positive sibling has at least one copy this gene
P_{A1}, P_{A2}, P_{A3}	=	probability that an individual will inherit a "susceptible allelic state" given that their sibling is known to be in this state (see text for definition) at dominant (P_{a1}), recessive (P_{a2}), and mixed (P_{a3}) non-HLA DRB1 loci.
**x** (x_1, x_2, x_3)	=	number of non-HLA DRB1susceptibility genetic loci involved in MS (dominant loci = x_1; recessive loci = x_2; mixed loci = x_3). [$x_1 + x_2 + x_3 = \mathbf{x}$]
P_{HM}	=	Probability that an individual (from the general population) is both susceptible to getting MS and carries the HLA DRB1*1501 allele. (if $Pt_1 = Pt_0$; then $P_{HM} = h_m$)
P_{AM}	=	Probability that an individual (from the general population) who is both susceptible to getting MS and is in a susceptible state at a specific non-HLA DRB1 locus. (if $Pt_1 = Pt_0$; then $P_{AM} = F_m$)
r	=	ratio of the "frequency of susceptibility" at the HLA DRB1 locus to the average "frequency of susceptibility" at other non-HLA DRB1 loci. [$r = h/F$]
**n** (n_1, n_2, n_3)	=	number of loci in "susceptible allelic states" required for MS to develop (dominant loci = n_1; recessive loci = n_2; mixed loci = n_3). [$n_1 + n_2 + n_3 = \mathbf{n}$]
P[**n**]	=	probability of an individual in the general population possessing at least **n** loci in a "susceptible allelic state"
C	=	proportion of patients, susceptible to MS, who do not have any copies of the HLA DRB1*1501 allele

P[S]	=	probability that an individual in the general population is susceptible to MS This probability is the same as P(G).
Pt	=	average penetrance of MS phenotype in susceptible patients. Also equal to the proband-wise monozygotic-twin concordance rate (CR_{MZ}).
Pt*	=	average penetrance of MS phenotype in susceptible patients, adjusted for non-twin siblings. [Pt* = (Pt) (2.9/5.4)] (See text)
Pt_1	=	average penetrance of MS phenotype in susceptible patients with at least one copy of the HLA DRB1*1501 allele. Also equal to the proband-wise monozygotic-twin concordance rate (Z_{H+}) for this genotype.
Pt_0	=	average penetrance of MS phenotype in susceptible patients without any copies of the HLA DRB1*1501 allele. Also equal to the proband-wise monozygotic-twin concordance rate (Z_{H-}) for this genotype
$P(MS_{H+})$	=	Probability of recurrence (i.e., the recurrence rate) in a family member of an MS proband who has at least one copy of the HLA DRB1*1501 allele.
$P(MS_{H-})$	=	Probability of recurrence (i.e., the recurrence rate) in a family member of an MS proband who lacks the HLA DRB1*1501 allele.
P(MS)	=	prevalence of the MS phenotype in the general population (equated to the life-time probability of getting MS)
P(G)	=	Probability of having any genotype capable of getting MS in response to some environmental exposure
P(E)	=	Probability of receiving any environmental exposure (all factors) sufficient to cause MS in some susceptible individual
CR_{MZ}	=	proband-wise monozygotic-twin concordance rate for MS.

CR_{IG}	=	proband-wise monozygotic-twin concordance rate for MS adjusted for impact of a shared intrauterine environment. [CR_{IG} = (CR_{MZ}) (2.9/5.4)] This variable is the identical to (Pt*) but is used for clarity of the text.
CR_{DZ}	=	proband-wise dizygotic-twin concordance rate for MS.
Z_{H+}	=	proband-wise monozygotic-twin concordance rate for MS when the proband possesses at least one copy of the HLA DRB1*1501 allele.
Z_{H-}	=	proband-wise monozygotic-twin concordance rate for MS when the proband does not possess a copy of the HLA DRB1*1501 allele.
CR_S	=	concordance rate for the MS phenotype in a non-twin sibling (1st degree)
CR_{PC}	=	concordance rate for the MS phenotype in a Parent or Child (1st degree)
CR_{AU}	=	concordance rate for the MS phenotype in an Aunt or Uncle (2nd degree)
CR_{FC}	=	concordance rate for the MS phenotype in a First Cousin (3rd degree)

Table 2. Epidemiological Data Used in the Model[‡]

	**Population**	**Men**	**Women**
Prevalence of MS [P(MS)] *	150	71.4	228.6
MZ twin Concordance (CR_{MZ}) *	25%	6.5%	34.0%
Raw % Susceptible [P(MS)] / CR_{MZ})*	0.6%	1.1%	0.7%
Corrected % Susceptible**	1.1%	2.0%	1.3%
% HLA DRB1*1501 (General Population) *	24%		
% HLA DRB1*1501 (MS Population) *	55%		
Homozygous DRB1*1501 (General Population) [†]	1.6%		
Homozygous DRB1*1501 (MS Population)[†]	10.0%		

‡ For estimated recurrence risks in 1st, 2nd, and 3rd degree relatives; see Table 12. For estimated recurrence risk in HLA DRB1*1501 positive and negative patients; see Table 3.

* From Canadian Data (11), based on prevalence of 150/100,000 population (16) and split into men and women according to (17). HLA data: D Sadovnick (personal communication)

** Percent of the population genetically susceptible to susceptible to MS [P(MS)]/CR_{MZ}] corrected (see text) for the reported (11) difference in concordance risk for DZ twins (5.4%) and non-twin siblings (2.9%)

† UCSF Database: J Oksenberg (personal communication)

Table 3. MS Concordance rates in Monozygotic Twins of HLA DRB1*1501-positive (Z_{H+}) and HLA DRB1*1501-negative (Z_{H-}) Probands *.

	**Monozygotic Twins of MS Probands**		
	**HLA DRB1*1501 Positive**	**HLA DRB1*1501 Negative**	**Totals**
**Concordant for MS (C)**	9	11	20
**Discordant for MS (D)**	31	42	73
**Totals**	40	53	93
**Pair-wise Concordance**†	Z_{H+} = (9/40) = 23%	Z_{H-} = (11/53) = 21%	
**Proband-wise Concordance**††	Z_{H+} = 31%	Z_{H-} = 29%	
**Proband-wise Concordance (Adjusted)** †††	Z_{H+} = 17%	Z_{H-} = 16%	

* Data derived from: Willer et al., 2003 (11)

† Pair-wise rates calculated as (Z = C/(C + D).

†† Proband-wise concordance rates calculated as (Z = 2C/(2C + D) adjusted (21) for the overall probability of doubly ascertaining concordant twin-pairs (54%) in the Willer, et al., 2003 (11) study.

††† See Text, Equation (20)

Table 4 (4a). The total number of non-HLA susceptibility genes (**x**) based on the number of susceptibility genes necessary for MS to develop (**n**), and the frequency of susceptibility at the non-HLA susceptibility locations in the population ($Pt_0 = Pt_1$).

	**Number of Susceptibility Genes Required (n)**						
	**5**	**10**	**11**	**12**	**13**	**14**	**15**
**Frequency of Susceptibility (r)**	**Estimated Total Number of non-HLA Susceptibility Genes (x)**						
r = 0.25	5	11	12	13	14-15	15-16	16-17
r = 0.33	6	12	13-14	14-15	16	17-18	18-19
r = 0.5	7	14-15	16-17	17-19	19-20	21-22	22-23
r = 1	11-12	23-25	25-27	28-30	30-32	33-35	35-37
r = 2	18-22	40-44	45-48	49-53	53-57	58-61	62-66
r = 4	35-42	75-83	83-91	92-99	100-107	108-116	116-124
r = 8	67-82	146-161	162-177	177-193	193-208	209-224	225-240
r = 16	131-162	287-317	318-348	349-379	380-410	411-441	442-472
**Frequency of Susceptibility (r)**	**Estimated Prevalence (Target = 0.1 - 0.2%)**						
r = 0.25	11.5%	12.7%	12.7%	12.4%	12.3 – 13.2%	12.1 – 13.2%	12.0 – 13.1%
r = 0.33	7.5%	5.2%	4.4 – 6.7%	3.7 – 5.9%	5.1%	4.4 – 6.4%	3.8 – 5.7%
r = 0.5	3.5%	1.2 – 2.0%	1.4 – 2.1%	0.9 – 2.2%	1.1 – 1.6%	1.2 – 1.7%	0.8 – 1.2%
r = 1	1.8 – 2.4%	0.6 – 1.0%	0.4 – 0.7%	0.4 – 0.7%	0.28 – 0.48%	0.27 – 0.45%	0.19 – 0.33%
r = 2	1.1 – 2.0%	0.3 – 0.6%	0.28 – 0.43%	0.21 – 0.37%	0.16 – 0.28%	0.14 – 0.21%	0.10 – 0.18%
r = 4	1.0 – 1.9%	0.26 – 0.47%	0.20 – 0.36%	0.16 – 0.27%	0.12 – 0.21%	0.10 – 0.17%	0.07 – 0.13%
r = 8	1.0 – 1.8%	0.24 – 0.42%	0.18 – 0.32%	0.14 – 0.25%	0.11 – 0.19%	0.08 – 0.14%	0.06 – 0.11%
r = 16	0.9 – 1.7%	0.23 – 0.40%	0.17 – 0.30%	0.13 – 0.23%	0.09 – 0.18%	0.08 – 0.13%	0.06 – 0.10%
**limit**	**1.27%**	**0.29%**	**0.22%**	**0.17%**	**0.13%**	**0.10%**	**0.07%**

Table 5 (4b)**.** The total number of non-HLA susceptibility genes (**x**) based on the number of susceptibility genes necessary for MS to develop (**n**), and the frequency of susceptibility at the non-HLA susceptibility locations in the population ($Pt_0 = Pt_1$).

	**Total Number Susceptibility Genes Required (n)**						
	**16**	**17**	**18**	**30**	**40**	**50**	**60**
**Frequency of Susceptibility (r)**	**Estimated Total Number of non-HLA Susceptibility Genes (x)**						
r = 0.25	18	19	20 - 21	33-35	44-46	56-58	67-71
r = 0.33	19-20	21	22 - 23	36-38	49-51	61-64	74-80
r = 0.5	24-25	25 - 26	27 - 28	44-47	60-62	75-78	91-101
r = 1	37-39	40-42	42 - 44	71-75	95-100	120-124	144-166
r = 2	66-70	71-74	75 - 79	125-133	168-176	212-220	255-300
r = 4	124-132	132-140	140 - 148	234-250	316-331	397-413	478-569
r = 8	240-256	256 - 271	272 - 287	453-484	611-642	769-800	926-1109
r = 16	473-503	504 - 535	535 - 566	892-953	1202-1264	1512-1574	1823-2000
**Frequency of Susceptibility (r)**	**Estimated Prevalence (Target = 0.1 - 0.2%)**						
r = 0.25	13.1%	13.1%	13.0 – 13.3%	13.0 – 13.4%	13.1 – 13.4%	13.4%	13.4%
r = 0.33	3.2 – 5.0%	4.4%	3.8 – 5.6%	1.7 – 3.5%	1.6 -3.4%	1.0 – 3.1%	1.0 – 5.4%
r = 0.5	0.9 – 1.3%	0.6 – 1.0%	0.7 – 1.0%	0.10 – 0.35%	0.05 – 0.11%	0.01 – 0.05%	0.00 – 0.20%
r = 1	0.14 – 0.24%	0.14 – 0.23%	0.10 – 0.17%	0.00 – 0.03%	0.00 – 0.00%	0.00 – 0.00%	0.00 – 0.00%
r = 2	0.07 – 0.14%	0.07 – 0.10%	0.05 – 0.09%	0.00 – 0.00%	0.00 – 0.00%	0.00 – 0.00%	0.00 – 0.00%
r = 4	0.06 – 0.10%	0.04 – 0.08%	0.03 – 0.06%	0.00 – 0.00%	0.00 – 0.00%	0.00 – 0.00%	0.00 – 0.00%
r = 8	0.05 – 0.09%	0.04 – 0.07%	0.03 – 0.05%	0.00 – 0.00%	0.00 – 0.00%	0.00 – 0.00%	0.00 – 0.00%
r = 16	0.05 – 0.08%	0.03 – 0.06%	0.03 – 0.05%	0.00 – 0.00%	0.00 – 0.00%	0.00 – 0.00%	0.00 – 0.00%
**limit**	**0.06%**	**0.04%**	**0.03%**	**0.00%**	**0.00%**	**0.00%**	**0.00%**

Table 6 (5a). Predicted concordance rates of MS in siblings of MS probands assuming ($Pt_0 = Pt_1$) and either 100% Dominant genes or 100% Recessive genes (Target = 2.9 – 3.8% is colored in yellow; optimal solution colored in green).

	Number of Susceptibility Genes Required (n)						
	**5**	**10**	**11**	**12**	**13**	**14**	**15**
**Frequency of Susceptibility (r)**	**Predicted Concordance in Non-twin Siblings (100% of Genes Dominant)**						
r = 0.25	12.6%	13.2%	13.2%	13.2%	13.1 – 13.4%	13.1 – 13.4%	13.0 – 13.4%
r = 0.33	10.9%	10.7%	9.8 – 11.4%	10.2%	10.7%	10.3 – 11.6%	9.9 – 11.3%
r = 0.5	8.5%	7.9 – 9.1%	7.7 – 8.9%	6.9 – 9.3%	7.5 – 8.6%	8.0 – 9.0%	7.3 – 8.4%
r = 1	7.4 – 8.2%	6.4 – 7.6%	6.3 – 7.4%	6.6 – 7.6%	6.3 – 7.3%	6.5 – 7.5%	6.2 – 7.2%
r = 2	6.6 – 8.0%	6.4 – 7.5%	6.4 – 7.1%	6.2 – 7.2%	6.1 – 7.0%	6.2 – 6.8%	6.0 – 6.9%
r = 4	6.7 – 7.9%	6.3 – 7.3%	6.1 – 7.1%	6.2 – 7.0%	6.1 – 6.9%	6.0 – 6.9%	6.0 – 6.8%
r = 8	6.6 – 7.9%	6.3 – 7.2%	6.2 – 7.1%	6.1 – 7.0%	6.0 – 6.9%	6.0 – 6.8%	6.0 – 6.7%
r = 16	6.6 – 7.9%	6.3 – 7.2%	6.1 – 7.0%	6.1 – 6.9%	6.0 – 6.8%	6.0 – 6.8%	5.9 – 6.7%
**Frequency of Susceptibility (r)**	**Predicted Concordance in Non-twin Siblings (100% of Genes Recessive)**						
r = 0.25	12.6%	13.2%	13.2%	13.1%	13.1- 13.4%	13.1 – 13.4%	13.2 – 13.4%
r = 0.33	10.8%	10.1%	9.6 – 11.3%	9.1 – 10.9%	10.5%	10.1 – 11.5%	9.7 – 11.2%
r = 0.5	8.1%	6.4 – 7.8%	7.0 – 8.2%	6.2 – 8.6%	6.7 – 7.9%	7.2 – 8.3%	6.5 – 7.6%
r = 1	6.4 – 7.2%	5.2 – 6.3%	4.8 – 5.9%	5.0 – 6.0%	4.6 – 5.6%	4.8 – 5.7%	4.5 – 5.4%
r = 2	5.0 – 6.5%	4.1 – 5.1%	4.1 – 4.8%	3.9 – 4.8%	3.7 – 4.5%	3.7 – 4.3%	3.5 – 4.3%
r = 4	4.8 – 6.0%	3.5 – 4.4%	3.3 – 4.2%	3.3 – 4.0%	3.1 – 3.8%	3.0 – 3.7%	2.8 – 3.5%
r = 8	4.3 – 5.7%	3.1 – 4.0%	3.0 – 3.7%	2.8 – 3.5%	2.6 – 3.3%	2.5 – 3.1%	2.4 – 3.0%
r = 16	4.0 – 5.4%	2.8 – 3.6%	2.7 –3.4%	2.5 – 3.2%	2.3 – 3.0%	2.2 – 2.8%	2.1 – 2.6%

Table 7 (5b). Predicted concordance rates of MS in siblings of MS probands assuming ($Pt_0 = Pt_1$) and either 100% Dominant genes or 100% Recessive genes (Target = 2.9 – 3.8% is colored in yellow).

	Number of Susceptibility Genes Required (n)						
	**16**	**17**	**18**	**30**	**40**	**50**	**60**
**Frequency of Susceptibility (r)**	**Predicted Concordance in Non-twin Siblings (100% of Genes Dominant)**						
r = 0.25	13.4%	13.4%	13.3 – 13.4%	13.4%	13.4%	13.4%	13.4%
r = 0.33	9.5 – 11.0%	10.7%	10.4 – 11.6%	9.4 – 11.6%	10.4 – 12.0%	10.2 – 12.3%	10.9 – 13.2%
r = 0.5	7.8 – 8.8%	7.2 – 8.2%	7.7 – 8.6%	6.2 – 8.6%	6.7 – 8.1%	6.4 – 8.3%	6.8 – 11.4%
r = 1	6.0 – 6.9%	6.2 – 7.1%	6.0 – 6.8%	5.5 – 6.8%	5.1 – 6.5%	5.1 – 6.1%	4.8 – 9.6%
r = 2	6.0 – 6.8%	6.0 – 6.6%	5.9 – 6.7%	5.2 – 6.4%	4.9 – 6.0%	4.8 – 5.7%	4.6 – 9.2%
r = 4	5.9 – 6.7%	5.8 – 6.6%	5.8 – 6.5%	5.1 – 6.3%	5.0 – 5.9%	4.8 – 5.6%	4.6 – 9.0%
r = 8	5.9 – 6.7%	5.9 – 6.6%	5.8 – 6.5%	5.1 – 6.2%	4.9 – 5.9%	4.8 – 5.6%	4.6 – 9.0%
r = 16	5.9 – 6.6%	5.8 – 6.6%	5.8 – 6.5%	5.2 – 6.2%	5.0 – 5.9%	4.8 – 5.6%	4.6 – 6.8%
**Frequency of Susceptibility (r)**	**Predicted Concordance in Non-twin Siblings (100% of Genes Recessive)**						
r = 0.25	13.4%	13.4%	13.3 – 13.4%	13.4%	13.4%	13.4%	13.4%
r = 0.33	9.2 – 10.8%	10.5%	10.1 – 11.4%	9.1 – 11.4%	10.5 – 11.8%	9.8 – 12.1%	10.5 – 13.1%
r = 0.5	7.0 – 8.0%	6.3 – 7.4%	6.8 – 7.8%	5.1 – 7.5%	5.2 – 6.7%	4.9 – 6.8%	5.1 – 10.3%
r = 1	4.2 – 5.0%	4.3 – 5.2%	4.1 – 4.9%	3.2 – 4.3%	2.6 – 3.7%	2.3 – 3.1%	1.9 – 6.2%
r = 2	3.3 – 4.1%	3.3 – 3.9%	3.2 – 3.9%	2.1 – 2.9%	1.6 – 2.2%	1.3 – 1.7%	1.0 – 4.0%
r = 4	2.7 – 3.3%	2.6 – 3.2%	2.5 – 3.0%	1.4 – 2.1%	1.0 – 1.5%	0.7 – 1.1%	0.5 – 2.7%
r = 8	2.3 – 2.8%	2.2 – 2.7%	2.1 – 2.5%	1.1 – 1.6%	0.7 – 1.0%	0.5 – 0.7%	0.3 – 1.9%
r = 16	2.0 – 2.5%	1.9 – 2.3%	1.8 – 2.2%	0.8 – 1.3%	0.5 – 0.8%	0.3 – 0.5%	0.2 – 0.6%

Table 8 (6a)**.** Predicted concordance rates of MS in first degree relatives of MS probands assuming ($Pt_0 = Pt_1$), 20% Dominant genes and 80% Recessive genes. (Targets are colored in yellow; optimal solution colored in green)

	**Number of Susceptibility Genes Required (n)**						
	**5**	**10**	**11**	**12**	**13**	**14**	**15**
**Frequency of Susceptibility (r)**	**Predicted Concordance in Non-twin Siblings (Target = 2.9 – 3.8%)**						
r = 0.25	12.6%	13.2%	13.2%	13.1%	13.1 – 13.4%	13.1 – 13.4%	13.0 – 13.4%
r = 0.33	10.8%	10.1%	9.6 – 11.3%	9.1 – 10.9%	10.5%	10.1 – 11.5%	9.7 – 11.2%
r = 0.5	8.1%	6.5 – 7.9%	7.1 – 8.3%	6.3 – 8.7%	6.8 – 8.0%	7.3 – 8.4%	6.6 – 7.8%
r = 1	6.5 – 7.3%	5.4 – 6.6%	5.1 – 6.2%	5.2 – 6.3%	4.9 – 5.9%	5.0 – 6.0%	4.8 – 5.7%
r = 2	5.2 – 6.7%	4.4 – 5.4%	4.5 – 5.2%	4.3 – 5.2%	4.0 – 4.9%	4.0 – 4.6%	3.9 – 4.7%
r = 4	5.0 – 6.2%	3.9 – 4.8%	3.8 – 4.7%	3.7 – 4.5%	3.5 – 4.2%	3.4 – 4.1%	3.3 – 4.0%
r = 8	4.6 – 5.9%	3.5 – 4.4%	3.5 – 4.3%	3.3 – 4.0%	3.1 – 3.8%	2.9 – 3.6%	2.9 – 3.5%
r = 16	4.3 – 5.6%	3.3 – 4.1%	3.2 – 4.0%	3.0 – 3.7%	2.8 – 3.5%	2.6 – 3.3%	2.6 – 3.2%
**Frequency of Susceptibility (r)**	**Predicted Concordance in Parents/Children (Target = 1.8 – 2.1%)**						
r = 0.25	12.6%	13.2%	13.2%	13.1%	13.1 – 13.4%	13.1 – 13.4%	13.0 – 13.4%
r = 0.33	10.7%	9.9%	9.4 – 11.2%	8.9 – 10.8%	10.4%	9.9 – 11.4%	9.5 – 11.0%
r = 0.5	7.7%	5.9 – 7.3%	6.5 – 7.8%	5.7 – 8.2%	6.2 – 7.4%	6.6 – 7.8%	6.0 – 7.1%
r = 1	5.6 – 6.4%	4.3 – 5.4%	3.9 – 5.0%	4.0 – 5.0%	3.6 – 4.5%	3.7 – 4.6%	3.4 – 4.3%
r = 2	3.7 – 5.3%	2.8 – 3.7%	2.9 – 3.6%	2.6 – 3.4%	2.4 – 3.1%	2.3 – 2.8%	2.2 – 2.8%
r = 4	3.3 – 4.5%	2.1 – 2.8%	2.0 – 2.7%	1.8 – 2.4%	1.6 – 2.1%	1.5 – 2.0%	1.4 – 1.9%
r = 8	2.7 – 3.9%	1.6 – 2.2%	1.5 – 2.1%	1.3 – 1.8%	1.1 – 1.6%	1.0 – 1.4%	1.0 – 1.3%
r = 16	2.3 – 3.5%	1.2 – 1.7%	1.2 – 1.7%	1.0 – 1.4%	0.8 – 1.2%	0.7 – 1.0%	0.7 – 0.9%

Table 9 (6b). Predicted concordance rates of MS in first degree relatives of MS probands assuming ($Pt_0 = Pt_1$), 20% Dominant genes and 80% Recessive genes. (Targets are colored in yellow)

	**Number of Susceptibility Genes Required (n)**						
	**16**	**17**	**18**	**30**	**40**	**50**	**60**
**Frequency of Susceptibility (r)**	**Predicted Concordance in Non-twin Siblings (Target = 2.9 – 3.8%)**						
r = 0.25	13.4%	13.4%	13.3 – 13.4%	13.4%	13.4%	13.4%	13.4%
r = 0.33	9.3 – 10.9%	10.5%	10.2 – 11.4%	9.9 – 11.0%	10.1 – 11.9%	9.9 – 12.1%	10.6 – 13.1%
r = 0.5	7.1 – 8.2%	6.5 – 7.5%	6.9 – 7.9%	5.3 – 7.7%	5.6 – 7.0%	5.2 – 7.1%	5.5 – 10.5%
r = 1	4.5 – 5.4%	4.6 – 5.5%	4.4 – 5.2%	3.5 – 4.7%	3.0 – 4.2%	2.8 – 3.6%	2.4 – 6.9%
r = 2	3.8 – 4.5%	3.8 – 4.3%	3.6 – 4.3%	2.5 – 3.5%	2.0 – 2.7%	1.7 – 2.3%	1.4 – 4.9%
r = 4	3.2 – 3.9%	3.1 – 3.7%	2.9 – 3.6%	1.9 – 2.7%	1.5 – 2.0%	1.2 – 1.6%	0.9 – 3.7%
r = 8	2.8 – 3.5%	2.7 – 3.3%	2.6 – 3.1%	1.5 – 2.2%	1.1 – 1.6%	0.8 – 1.2%	0.6 – 2.9%
r = 16	2.5 – 3.1%	2.4 – 2.9%	2.3 – 2.8%	1.3 – 1.9%	0.9 – 1.3%	0.6 – 0.9%	0.5 – 1.1%
**Frequency of Susceptibility (r)**	**Predicted Concordance in Parents/Children (Target = 1.8 – 2.1%)**						
r = 0.25	13.4%	13.4%	13.3 – 13.4%	13.4%	13.4%	13.4%	13.4%
r = 0.33	9.1 – 10.7%	10.4%	10.0 – 11.3%	8.8 – 11.2%	9.3 – 11.3%	9.6 – 11.9%	10.3 – 13.0%
r = 0.5	6.4 – 7.5%	5.8 – 6.8%	6.2 – 7.2%	4.4 – 6.7%	4.6 – 5.9%	4.1 – 5.9%	4.2 – 9.5%
r = 1	3.2 – 4.0%	3.3 – 4.0%	3.0 – 3.7%	2.0 – 3.0%	1.5 – 2.3%	1.2 – 1.7%	0.9 – 4.1%
r = 2	2.1 – 2.9%	2.0 – 2.4%	1.8 – 2.4%	0.9 – 1.4%	0.6 – 0.9%	0.4 – 0.6%	0.2 – 1.7%
r = 4	1.4 – 1.8%	1.2 – 1.6%	1.1 – 1.5%	0.4 – 0.7%	0.2 – 0.4%	0.1 – 0.2%	0.1 – 0.7%
r = 8	0.9 – 1.3%	0.8 – 1.1%	0.7 – 1.0%	0.2 – 0.4%	0.1 – 0.2%	0.0 – 0.1%	0.0 – 0.3%
r = 16	0.7 – 0.9%	0.6 – 0.8%	0.5 – 0.7%	0.1 – 0.2%	0.0 – 0.1%	0.0%	0.0%

Table 10 (7a)**.** Predicted concordance rates of MS in second and third degree relatives of MS probands assuming ($Pt_0 = Pt_1$), 20% Dominant genes and 80% Recessive genes. (Targets are colored in yellow; optimal solution colored in green)

	**Number of Susceptibility Genes Required (n)**						
	**5**	**10**	**11**	**12**	**13**	**14**	**15**
**Frequency of Susceptibility (r)**	**Predicted Concordance in Second Degree Relatives (Target = 0.9 – 1.6%)**						
r = 0.25	12.1%	13.0%	12.9%	12.8%	12.8 – 13.3%	12.7–13.3%	12.6 – 13.3%
r = 0.33	9.2%	7.6%	6.8 – 9.1%	6.1 – 8.4%	7.8%	7.1 – 9.1%	6.5 – 8.5%
r = 0.5	5.6%	3.2 – 4.4%	3.5 – 4.7%	2.8 – 5.0%	3.1 – 4.1%	3.4 – 4.4%	2.7 – 3.7%
r = 1	3.7 – 4.5%	2.1 – 2.9%	1.8 – 2.5%	1.8 – 2.4%	1.5 – 2.1%	1.5 – 2.0%	1.3 – 1.8%
r = 2	2.5 – 3.9%	1.4 – 2.0%	1.4 – 1.8%	1.2 – 1.7%	1.0 – 1.4%	0.9 – 1.2%	0.8 – 1.2%
r = 4	2.4 – 3.5%	1.1 – 1.7%	1.0 – 1.5%	0.9 – 1.3%	0.8 – 1.1%	0.7 – 1.0%	0.6 – 0.9%
r = 8	2.1 – 3.2%	1.0 – 1.4%	0.9 – 1.3%	0.7 – 1.1%	0.6 – 0.9%	0.5 – 0.8%	0.5 – 0.7%
r = 16	1.9 – 3.0%	0.9 – 1.3%	0.8 – 1.1%	0.6 – 0.9%	0.5 – 0.8%	0.5 – 0.7%	0.4 – 0.6%
**Frequency of Susceptibility (r)**	**Predicted Concordance in Third Degree Relatives (Target = 0.9%)**						
r = 0.25	11.8%	12.9%	12.8%	12.7%	12.5 – 13.3%	12.4 – 13.2%	12.3 – 13.2%
r = 0.33	8.3%	6.3%	5.5 – 7.9%	4.8 – 7.1%	6.4%	5.7 – 7.8%	5.0 – 7.1%
r = 0.5	4.4%	2.0 – 3.0%	2.3 – 3.3%	1.7 – 3.4%	1.9 – 2.7%	2.1 – 2.9%	1.6 – 2.2%
r = 1	2.7 – 3.3%	1.1 – 1.7%	0.9 – 1.4%	0.9 – 1.3%	0.7 – 1.1%	0.7 – 1.0%	0.5 – 0.8%
r = 2	1.7 – 2.8%	0.7 – 1.1%	0.6 – 0.9%	0.5 – 0.8%	0.4 – 0.7%	0.4 – 0.5%	0.3 – 0.5%
r = 4	1.6 – 2.5%	0.5 – 0.9%	0.5 – 0.7%	0.4 – 0.6%	0.3 – 0.5%	0.3 – 0.4%	0.2 – 0.3%
r = 8	1.4 – 2.4%	0.5 – 0.8%	0.4 – 0.6%	0.3 – 0.5%	0.3 – 0.4%	0.2 – 0.3%	0.2 – 0.3%
r = 16	1.3 – 2.2%	0.4 – 0.7%	0.3 – 0.5%	0.3 – 0.5%	0.2 – 0.4%	0.2 – 0.3%	0.1 – 0.2%

Table 11 (7b)**.** Predicted concordance rates of MS in second and third degree relatives of MS probands assuming ($Pt_0 = Pt_1$), 20% Dominant genes and 80% Recessive genes. (Targets are colored in yellow)

	**Number of Susceptibility Genes Required (n)**						
	**16**	**17**	**18**	**30**	**40**	**50**	**60**
**Frequency of Susceptibility (r)**	**Predicted Concordance in Second Degree Relatives (Target = 0.9 – 1.6%)**						
r = 0.25	13.3%	13.2%	13.2 – 13.4%	13.2 – 13.4%	13.3 – 13.4%	13.4%	13.4%
r = 0.33	5.9 – 8.0%	7.4%	6.8 – 8.6%	4.6 – 7.6%	5.0 – 7.7%	4.3 – 7.8%	4.6 – 10.5%
r = 0.5	3.0 – 3.9%	2.4 – 3.3%	2.7 – 3.5%	1.1 – 2.3%	0.9 – 1.4%	0.5 – 1.1%	0.4 – 3.0%
r = 1	1.1 – 1.5%	1.1 – 1.5%	0.9 – 1.3%	0.3 – 0.6%	0.1 – 0.3%	0.1%	0.0 – 0.1%
r = 2	0.7 – 1.0%	0.7 – 0.9%	0.6 – 0.8%	0.1 – 0.3%	0.1%	0.0%	0.0%
r = 4	0.5 – 0.8%	0.5 – 0.7%	0.4 – 0.6%	0.1 – 0.2%	0.0 – 0.1%	0.0%	0.0%
r = 8	0.4 – 0.6%	0.4 – 0.5%	0.3 – 0.5%	0.1%	0.0%	0.0%	0.0%
r = 16	0.4 – 0.5%	0.3 – 0.5%	0.3 – 0.4%	0.0 – 0.1%	0.0%	0.0%	0.0%
**Frequency of Susceptibility (r)**	**Predicted Concordance in Third Degree Relatives (Target = 0.9%)**						
r = 0.25	13.2%	13.1%	13.1 – 13.4%	13.1 – 13.4%	13.2 – 13.4%	13.4%	13.4%
r = 0.33	4.5 – 6.4%	5.8%	5.2 – 7.1%	2.9 – 5.6%	3.1 – 5.4%	2.2 – 5.2%	2.4 – 8.0%
r = 0.5	1.7 – 2.4%	1.3 – 1.9%	1.4 – 2.0%	0.4 – 1.0%	0.2 – 0.4%	0.1 – 0.2%	0.1 – 1.0%
r = 1	0.4 – 0.7%	0.4 – 0.6%	0.3 – 0.5%	0.1%	0.0%	0.0%	0.0%
r = 2	0.3 – 0.4%	0.2 – 0.3%	0.2 – 0.3%	0.0 – 0.1%	0.0%	0.0%	0.0%
r = 4	0.2 – 0.3%	0.1 – 0.2%	0.1 – 0.2%	0.0%	0.0%	0.0%	0.0%
r = 8	0.1 – 0.2%	0.1 – 0.2%	0.1 – 0.2%	0.0%	0.0%	0.0%	0.0%
r = 16	0.1 – 0.2%	0.1 – 0.2%	0.1%	0.0%	0.0%	0.0%	0.0%

Table 12 (8**).** Observed and the optimal predicted concordance rates and prevalence rates for MS under different conditions.

	Observed (Estimated)*	100% Recessive	80% Recessive	100% Dominant	100% Mixed
Number Genes Needed (**n**)	-	14	13	58	58
Frequency of Susceptibility (**r**)	-	2	4	2	2
Total Non-HLA Genes (**x**)	-	58 – 61	100 – 107	246-254	246 - 254
**Relationship**					
Prevalence (2, 19)	0.1 – 0.2% (1.5)	0.14 – 0.21%	0.12 – 0.21%	0.00%	0.00%
Non-twin Sibling (5, 6)	2.9 – 3.8% (3.0)	3.7 – 4.3%	3.3 – 4.1%.	4.6 – 5.4%	4.5 – 5.3%
Offspring, Conjugal MS**	~10% (10.0)	11.9 – 12.1%	10.3 – 10.8%	13.4%	13.2 – 13.3%
Parent/Child (5)	1.8 – 2.1% (2.0)	1.8 – 2.3%	1.3 – 1.8%	4.6 – 5.4%	4.3 – 5.1%
Second Degree (5)	0.9 – 1.6% (1.0)	0.8 – 1.1%	0.7 – 1.1%	0.1%	0.1%
Third Degree (5)	0.9% (0.9)	0.3 – 0.5%	0.3 – 0.5%	0.00%	0.00%
Closeness of Fit	-	1.8	1.7	22.7	20.6

* The estimates (Targets) used to calculate closeness of fit are shown in parentheses. Closeness of fit was measured as the sum of the squared percent deviations of both the high and the low prediction from the Target. The optimal estimate was taken as the estimate at the values of x, n, and r that gave the closest fit to the observations.

** The concordance rate for the offspring of Conjugal MS is based on the report of Sadovnick et al. (6), in which the recurrence rate in offspring of two parents with MS is reported to be 78% of the monozygotic twin rate (CR_{MZ}).

Table 13 (9). "Closeness of Fit" Calculations.* (Targets are colored in yellow; optimal fit colored in green)

	**Number of Susceptibility Genes Required (n)**						
	**5**	**10**	**11**	**12**	**13**	**14**	**15**
**Frequency of Susceptibility (r)**	**Closeness of Fit (Target ≤ 4.0)**						
r = 0.25	24,106	29,422	28,838	28,228	29,694	29,258	28,805
r = 0.33	19,148	4,872	5,600	4,166	4,788	5,416	4,150
r = 0.5	2,139	459	555	451	320	378	189
r = 1	789	90	43.9	39.7	18.9	17.7	9.4
r = 2	394	21.4	12.1	6.9	3.3	2.4	2.5
r = 4	337	9.9	4.4	2.2	1.7	2.1	3.0
r = 8	286	6.8	3.1	1.9	2.3	3.4	4.4
r = 16	262	5.8	2.8	2.4	3.3	4.6	5.8
	**Number of Susceptibility Genes Required (n)**						
	**16**	**17**	**18**	**30**	**40**	**50**	**60**
**Frequency of Susceptibility (r)**	**Closeness of Fit (Target ≤ 4.0)**						
r = 0.25	31,198	30,935	31,545	31,749	32,041	32,713	32,776
r = 0.33	3,137	3,593	4,106	1,459	1,280	885	2,042
r = 0.5	228	114	140	21.4	18.3	18.7	38.0
r = 1	5.9	6.2	4.7	8.5	10.6	11.8	11.6
r = 2	3.0	3.6	4.2	10.9	13.5	14.9	12.7
r = 4	4.0	5.1	6.3	13.2	15.5	16.8	14.9
r = 8	5.5	6.8	8.0	14.7	16.7	17.7	16.2
r = 16	6.8	8.1	9.4	15.6	17.3	18.2	18.3

* Calculated as the sum of the squared percent deviations from published epidemiological observations (E) of the high (H) and low (L) estimates derived from the model for non-twin siblings, parents/children, offspring of conjugal MS couples, and second and third degree relatives of MS probands. For each category, this squared percent deviation is defined as:

$$[(H - E)/E + (L - E)/E]^2$$

Table 14 (10)**.** The Estimated Prevalence and the Number of Loci (**n**) for susceptible genotypes that include the HLA DRB1*1501 allele. (Yellow and green colors as designated for "Closeness of Fit" calculations in Table 8)

	**Estimated Number of Susceptibility Genes Required (n) for all Loci**						
	**11**	**12**	**13**	**14**	**15**	**16**	**17**
**Frequency of Susceptibility (r)**	**Estimated Prevalence HLA DRB1*1501 in an MS Population (unadjusted; OR ≈ 2.21)**						
r = 0.25	0.25	0.26	0.24 – 0.26	0.24 – 0.26	0.24 – 0.26	0.25	0.25
r = 0.33	0.32 – 0.37	0.33 – 0.39	0.35	0.32 – 0.36	0.33 – 0.37	0.34 – 0.38	0.34
r = 0.5	0.39 – 0.42	0.38 – 0.44	0.39 – 0.42	0.39 – 0.41	0.39 – 0.42	0.38 – 0.41	0.40 – 0.42
r = 1	0.42 – 0.44	0.41 – 0.43	0.41 – 0.44	0.41 – 0.43	0.41 – 0.43	0.42 – 0.44	0.41 – 0.43
r = 2	0.42 – 0.44	0.41 – 0.44	0.41 – 0.44	0.42 – 0.43	0.41 – 0.43	0.42 – 0.43	0.42 – 0.43
r = 4	0.42 – 0.44	0.42 – 0.44	0.42 – 0.44	0.41 – 0.43	0.42 – 0.43	0.42 – 0.43	0.42 – 0.43
r = 8	0.42 – 0.44	0.42 – 0.44	0.41 – 0.43	0.42 – 0.43	0.42 – 0.43	0.41 – 0.43	0.42 – 0.43
r = 16	0.42 – 0.44	0.42 – 0.44	0.42 – 0.43	0.42 – 0.43	0.42 – 0.43	0.42 – 0.43	0.41 – 0.43
**Frequency of Susceptibility (r)**	**Odd Ratios for Recessive non-HLA DRB1 Loci (after adjustment of HLA to OR = 3.34)**						
r = 0.25	1.21	1.23	1.05 – 1.23	1.05 – 1.27	1.07 – 1.29	1.07	1.08
r = 0.33	1.57 – 2.03	1.66 – 2.15	1.66 – 2.15	1.51 – 1.82	1.57 – 1.91	1.64 – 2.00	1.70
r = 0.5	1.88 – 2.15	1.79 – 2.30	1.79 – 2.30	1.82 – 2.01	1.91 – 2.12	1.84 – 2.01	1.92 – 2.11
r = 1	1.86 – 2.05	1.82 – 1.98	1.82 – 1.98	1.82 – 1.96	1.85 – 1.98	1.88 – 2.01	1.84 – 1.96
r = 2	1.75 – 1.85	1.73 – 1.85	1.73 – 1.85	1.75 – 1.83	1.74 – 1.84	1.74 – 1.84	1.75 – 1.82
r = 4	1.65 – 1.78	1.66 – 1.75	1.66 – 1.75	1.65 – 1.74	1.66 – 1.74	1.66 – 1.74	1.66 – 1.73
r = 8	1.60 – 1.69	1.60 – 1.69	1.60 – 1.69	1.60 – 1.67	1.60 – 1.67	1.60 – 1.67	1.61 – 1.66
r = 16	1.56 – 1.64	1.56 – 1.64	1.56 – 1.64	1.56 – 1.63	1.56 – 1.62	1.57 – 1.62	1.56 – 1.62

Table 15 (11**).** The estimated Number of Loci (**n**) in for Genotypes including or not including HLA DRB1*1501. (Yellow and green colors as designated for "Closeness of Fit" calculations in Table 8)

	**Estimated Number of Susceptibility Genes Required (n) for all Loci**						
	**11**	**12**	**13**	**14**	**15**	**16**	**17**
**Frequency of Susceptibility (r)**	**Estimated (n) the for Genotypes including HLA DRB1*1501**						
r = 0.25	3	3	3	3	3	3	3
r = 0.33	3 – 10	3 – 11	11 – 11	3 – 13	11 – 14	14 – 15	15
r = 0.5	10 – 11	11 – 12	12 – 13	13 – 14	14 – 15	15 – 16	17
r = 1	11	12	13	14	15	16	17
r = 2	11	12	13	14	15	16	17
r = 4	11	12	13	14	15	16	17
r = 8	11	12	13	14	15	16	17
r = 16	11	12	13	14	15	16	17
**Frequency of Susceptibility (r)**	**Estimated (n) the for Genotypes not including HLA DRB1*1501**						
r = 0.25	12	13	14 – 15	15 – 16	16 – 17	18	19
r = 0.33	11 – 12	12 – 13	14	15	15 – 16	16 – 17	18
r = 0.5	11	12	13	14	15	16	17
r = 1	11	12	13	14	15	16	17
14 – 15r = 2	11	12	13	14	15	16	17
r = 4	11	12	13	14	15	16	17
r = 8	11	12	13	14	15	16	17
r = 16	11	12	13	14	15	16	17
